# Strength training alters the tissue fatty acids profile and slightly improves the thermogenic pathway in the adipose tissue of obese mice

**DOI:** 10.1038/s41598-022-10688-w

**Published:** 2022-04-28

**Authors:** Diego Gomes de Melo, Chadi Pellegrini Anaruma, Kellen Cristina da Cruz Rodrigues, Rodrigo Martins Pereira, Thais Dantis Pereira de Campos, Raphael Santos Canciglieri, Camila Oliveira Ramos, Dennys Esper Cintra, Eduardo Rochete Ropelle, Adelino Sanchez Ramos da Silva, José Rodrigo Pauli, Leandro Pereira de Moura

**Affiliations:** 1 Exercise Cellular Biology Laboratory, University of Campinas, Limeira, São Paulo Brazil; 2 Laboratory of Molecular Biology of Exercise, School of Applied Sciences, University of Campinas, Limeira, São Paulo Brazil; 3 Department of Physical Education, Institute of Biosciences, São Paulo State University (UNESP), Rio Claro, São Paulo Brazil; 4 Laboratory of Nutritional Genomics, School of Applied Sciences, University of Campinas, Limeira, São Paulo Brazil; 5 Postgraduate Program in Rehabilitation and Functional Performance, Ribeirão Preto Medical School, USP, Ribeirão Preto, São Paulo Brazil; 6 School of Physical Education and Sport of Ribeirão Preto, University of São Paulo (USP), Ribeirão Preto, São Paulo Brazil

**Keywords:** Cell signalling, Endocrine system and metabolic diseases, Diabetes, Diabetes complications, Type 2 diabetes, RNA metabolism, Endocrine system and metabolic diseases, Obesity, Cell biology, Molecular biology, Diseases, Endocrinology, Disease prevention, Nutrition

## Abstract

Obesity is a disease characterized by the exacerbated increase of adipose tissue. A possible way to decrease the harmful effects of excessive adipose tissue is to increase the thermogenesis process, to the greater energy expenditure generated by the increase in heat in the body. In adipose tissue, the thermogenesis process is the result of an increase in mitochondrial work, having as substrate H^+^ ions, and which is related to the increased activity of UCP1. Evidence shows that stress is responsible for increasing the greater induction of UCP1 expression via β-adrenergic receptors. It is known that physical exercise is an important implement for sympathetic stimulation promoting communication between norepinephrine/epinephrine with membrane receptors. Thus, the present study investigates the influence of short-term strength training (STST) on fatty acid composition, lipolysis, lipogenesis, and browning processes in the subcutaneous adipose tissue (sWAT) of obese mice. For this, Swiss mice were divided into three groups: lean control, obesity sedentary, and obese strength training (OBexT). Obese animals were fed a high-fat diet for 14 weeks. Trained obese animals were submitted to 7 days of strength exercise. It was demonstrated that STST sessions were able to reduce fasting glycemia. In the sWAT, the STST was able to decrease the levels of the long-chain fatty acids profile, saturated fatty acid, and palmitic fatty acid (C16:0). Moreover, it was showed that STST did not increase protein levels responsible for lipolysis, the ATGL, ABHD5, pPLIN1, and pHSL. On the other hand, the exercise protocol decreased the expression of the lipogenic enzyme SCD1. Finally, our study demonstrated that the STST increased browning process-related genes such as PGC-1α, PRDM16, and UCP1 in the sWAT. Interestingly, all these biomolecular mechanisms have been observed independently of changes in body weight. Therefore, it is concluded that short-term strength exercise can be an effective strategy to initiate morphological changes in sWAT.

## Introduction

Obesity is a pandemic condition affecting different populations worldwide, exceeding one billion people^1,2^. It is a disease characterized mainly by a disruption in insulin signaling in the hypothalamus and peripheral tissues, culminating in exacerbated body fat accumulation by changes in food intake and energy expenditure, which leads to several physiological and molecular disarrangement^3^. The increment of fatty acids in the adipose tissue exacerbates the subclinical inflammatory process associated with saturated fatty acid intake, leading to the decrease of insulin sensitivity and glucose uptake, which can culminate in diseases such as type 2 diabetes^4,5^.

In this sense, increasing the thermogenesis process can be a strategy to reduce the harmful effects of adipose tissue accumulation^6^. This is due to the greater energy expenditure generated by the increase in heat in the body^7,8^. In adipose tissue, the thermogenesis process is the result of an increase in mitochondrial work, having as substrate H^+^ ions, which is processed by uncoupling proteins (UCPs)^7,8^. The process occurs due to increased activity of the uncoupling protein-1 (UCP1), peroxisome proliferator-activated receptor-gamma (PPARγ), peroxisome proliferator-activated receptor gamma-coactivator 1 alpha (PGC-1α), and PR domain containing 16 (PRDM16) on brown adipose tissue (BAT)^9,10^. Therefore, due to pharmacological effects, stress, hypothermia, and physical exercise, the subcutaneous white adipose tissue (sWAT) can undergo modifications, such as increasing the UCP1 and PGC-1α levels and thermogenesis^11–13^. This molecular process is known as the browning of sWAT, and originates a new adipose tissue, the beige adipose tissue^14,15^. Derived from white adipose tissue, beige adipose tissue has similar molecular characteristics to BAT^16^.

Consequently, knowing molecules that can stimulate mainly UCP1 expression becomes important to increase the range of strategies to fight against obesity and diabetes^15,17^. Knowing this, Granneman et al. investigated the effects of β-adrenergic receptor stimulant (CL316,243) in mice C57BL/6, the authors observed that regardless of the increase in UCP1 expression, there was a temporary increase in fatty acid esterification, fatty acid catabolism, and glycerol metabolism after 7 days of intervention characteristic aspects of de novo lipogenesis (DNL)^18^. In contrast, Guilherme and colleagues observed that the absence of FASN (FASNKO) production reduces the differentiation of white adipose tissue and the increase of thermogenesis in FASN^flox/flox^ mice, however, when treated β-adrenergic receptor stimulant (CL316,243) and exposed to a temperature of 22 °C the animals were able to promote greater activation of the sympathetic nerves increasing the activity of de novo lipogenesis in iWAT^19^. In addition, the author also observed the increased differentiation of white adipose into multilocular, the increased protein, and gene expression of UCP1 in iWAT^19,20^. Concomitantly, according to Syrový and colleagues, the activation of the proteins responsible for the *Ucp1* gene transcription reduces the Acetyl-CoA carboxylase (ACC) and Fatty acid synthase (FAS) enzyme activities^16^. Thus, a mechanism that increases UCP1 expression is capable of reducing the ACC protein involved in the hepatic lipogenesis process^21^. Furthermore, the expression of UCP1 and browning stimulus in sWAT promotes the decrease of tumor necrosis factor-alpha (TNF-α), insulin resistance, and regulates mitochondrial dysfunction^22^.

Therefore, knowing non-pharmacological strategies or molecules that stimulate UCP1 increasing is important to increase the basal energy expenditure of obese people^23^. Nonetheless, it is well known that aerobic physical exercise is an essential non-pharmacological stimulus responsible for generating genetic, physiological, and morphological adaptations, such as increased energy expenditure by enhancing the degradation of fatty acids and improving insulin signaling in several tissues^24–26^. However, the effects of strength exercise on the induction of factors related to the increase of UCP1 expression in the sWAT were not explored yet.

Moreover, it has been shown that the browning of sWAT is dependent on PPARγ/PGC-1α/PRDM complex protein activity leading to increased *Ucp1* gene expression both in vivo and in vitro^26^*.* This phenomenon is stimulated by irisin derived from skeletal muscle of chronic animal training (swimming or running exercise) that can increase caloric expenditure and insulin sensitivity^27,28^. However, the effects of strength training on browning of sWAT, without body weight alterations, have not been described in the scientific literature. Therefore, firstly, the present study aims to assess whether 7 days of strength training can stimulate the browning process in subcutaneous adipose tissue. Secondly, to analyze whether there was an alteration in fat tissue profile and its synthesis and degradation in this fat tissue. Importantly, all observations were carried out without interference of body adipose changes.

## Results

### The effects of obesity and STST on physiological parameters

**Figure 1:**
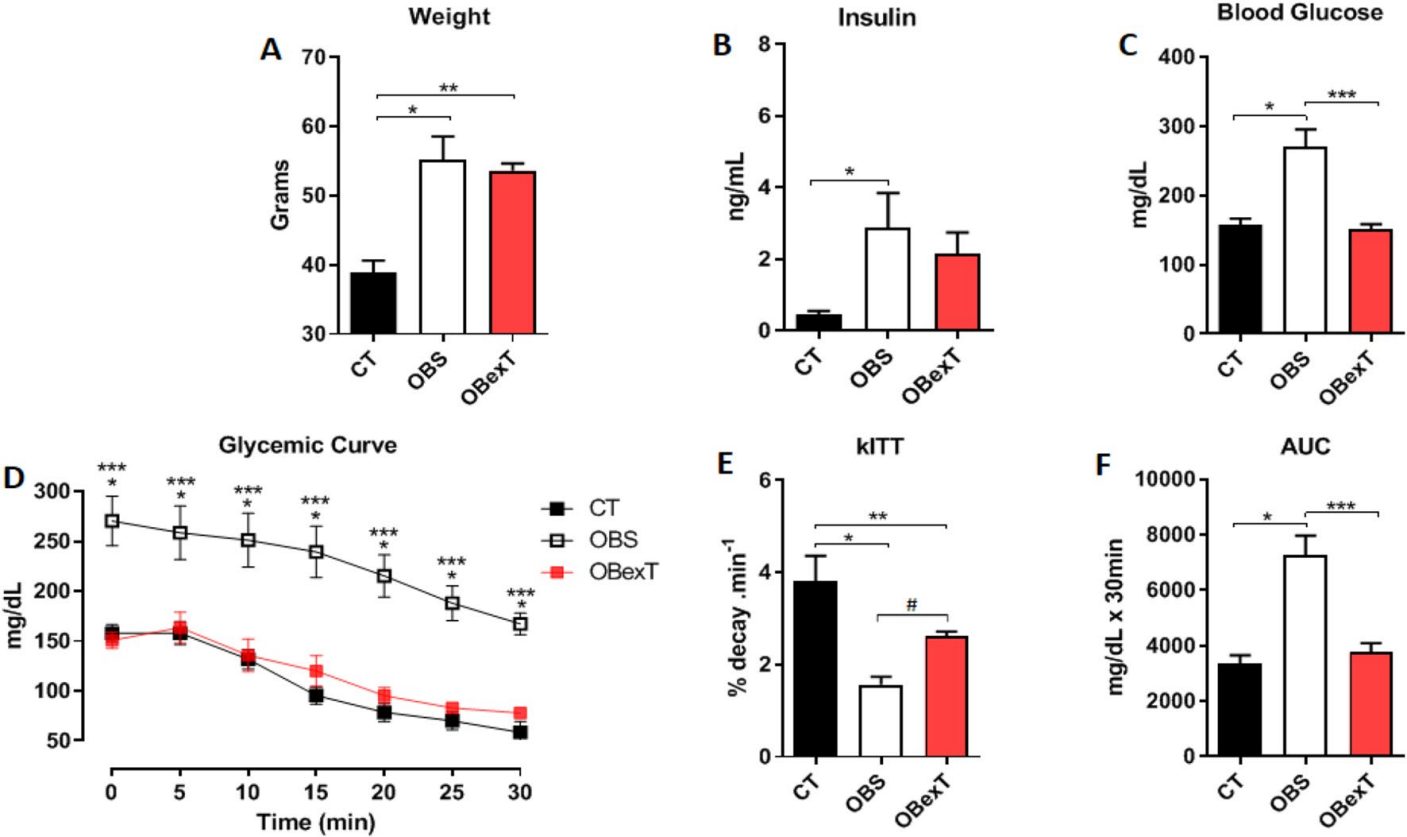
Effects of obesity and strength training on (**A**) Body weight, (**B**) Fasting insulin (8 h), (**C**) Fasting blood glucose (8 h), (**D**) Glycemic curve, (**E**) kITT and (**F**) AUC. The signal of statistical difference are represented as follows *p < 0.05 CT versus OBS, **p < 0.05 CT versus OBexT and ***p < 0.05 OBS versus OBexT, ^#^*p* = 0.054 (n = 4 CT group; n = 5 OBS group; n = 5 OBexT group). Anova one-way statistical analysis was used, (Glycemic curve used Anova two-way) followed by Tukey’s post-test.

Initially, during 14 weeks, the animals were induced to obesity and insulin resistance conditions. After that, the animals of the obese trained group (OBexT) initiated the short-term strength training protocol. After 14 weeks on a high-fat diet, as expected, the obese group demonstrated a series of harmful characteristics resulting from obesity, such as increased body weight (Fig. 1A), fasting hyperinsulinemia (Fig. 1B), hyperglycemia (Fig. 1C), and insulin resistance (Fig. 1D–F). On the other hand, it was shown that this training protocol decreased fasting hyperglycemia (Fig. 1C). The insulin resistance condition was verified by using the glucose decay curve graph. It was possible to observe that the obese group decreased the rate of glucose uptake after insulin administration and that the STST ameliorated this condition (Fig. 1D–F). As expected, there was no difference in body weight between both obese groups at the end of the study.

**Figure 2:**
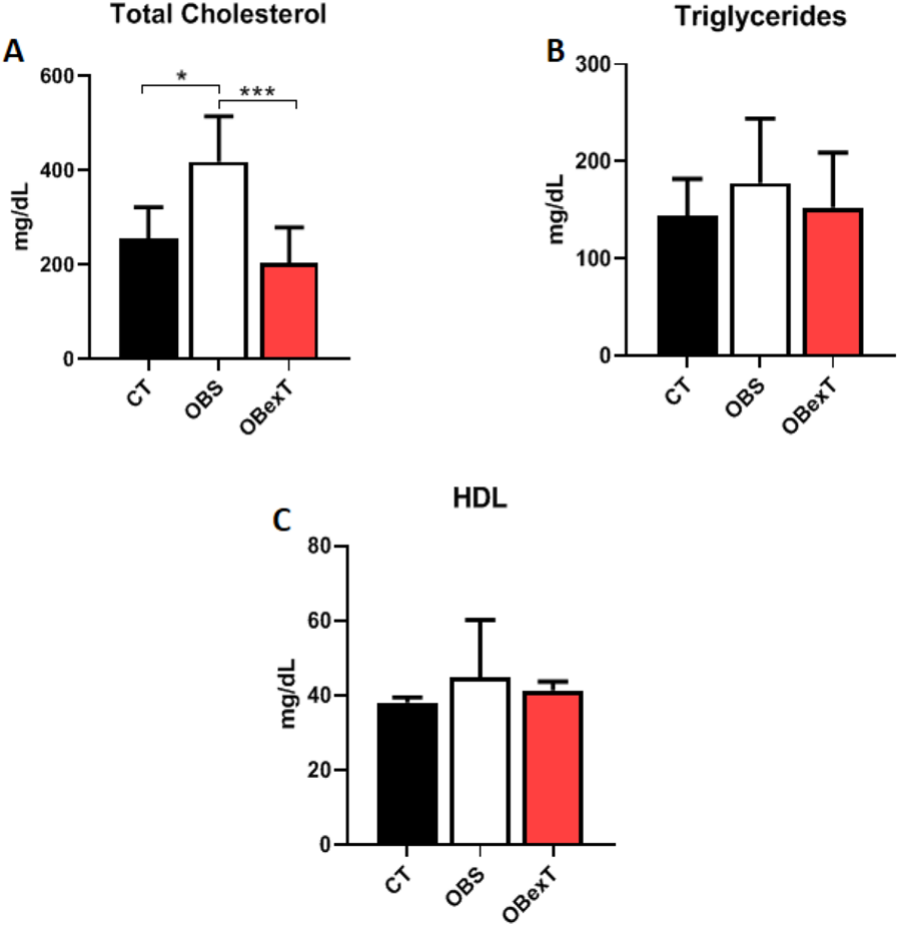
Effects of obesity and strength training on (**A**) Total Cholesterol, (**B**) Triglycerides, (**C**) High-Density Lipoproteins (HDL). The signal of statistical difference are represented as follows *p < 0.05 CT versus OBS, **p < 0.05 CT versus OBexT and ***p < 0.05 OBS versus OBexT (n = 5 CT group; n = 6 OBS group; n = 6 OBexT group). One-way Anova statistical analysis was used, followed by Bonferroni post-test.

Furthermore, it was analyzed the serum lipid profile. Obese animals increased total cholesterol (Fig. 2A) levels when compared to the control group and the exercise protocol decreased these parameters. Moreover, no difference was found in serum triglycerides (Fig. 2B) and HDL (Fig. 2C).

### The effects of obesity and STST on the sWAT fatty acids profile

**Figure 3:**
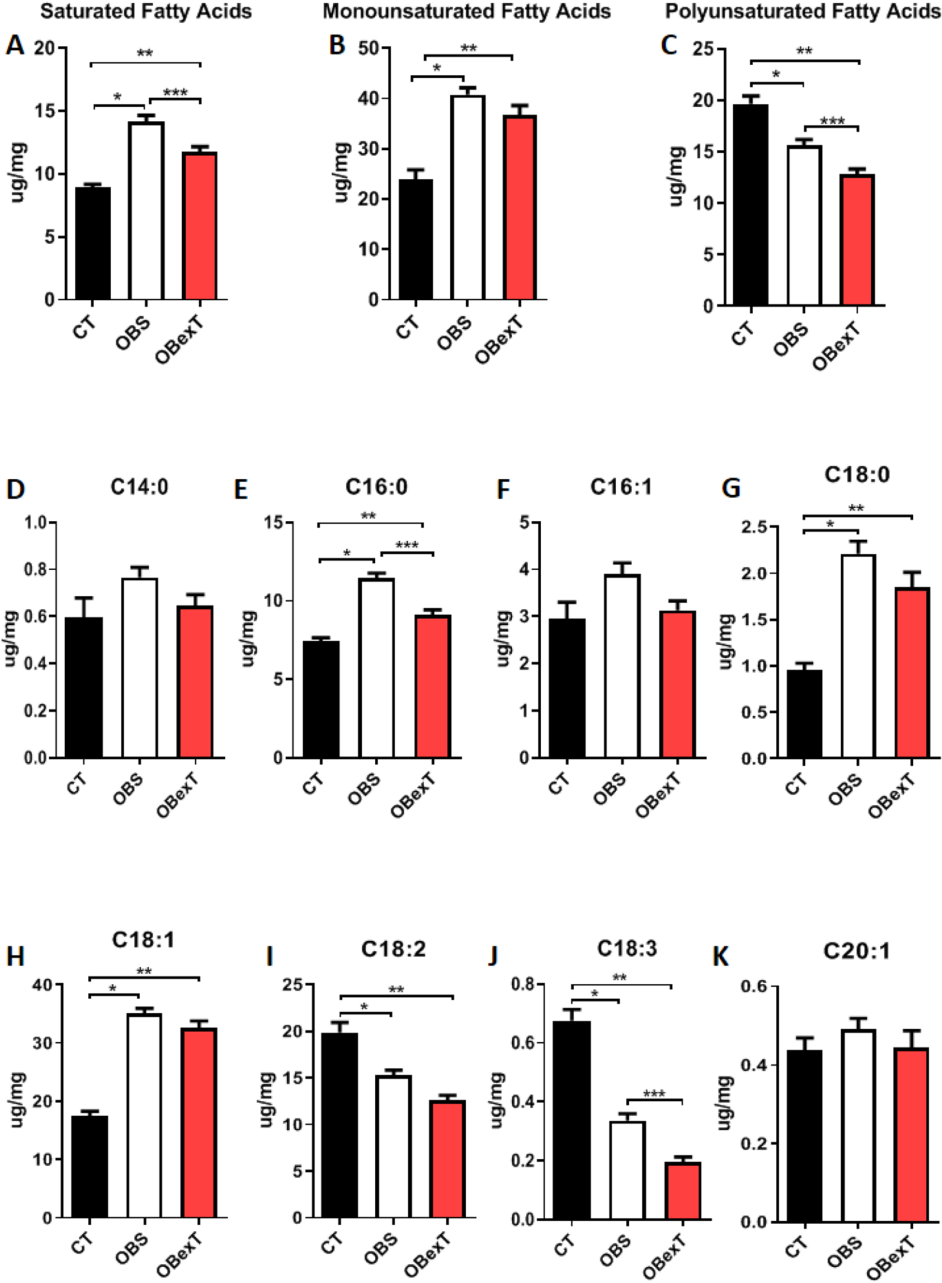
Subcutaneous adipose tissue fatty acids profile. (**A**) Total saturated fatty acids, (**B**) Total monounsaturated fatty acids, (**C**) Total polyunsaturated fatty acids, (D) Myristic fatty acids (C14:0), (**E**) Palmitic fatty acids (C16:0), (**F**) Palmitoleic fatty acids (C16:1), (**G**) Stearic fatty acids (C18:0), (**H**) Oleic fatty acids (C18:1), (**I**) Linoleic fatty acids (C18:2), (**J**) α-Linolenic fatty acids (C18:3) and (**K**) Eicosanoic fatty acids (C20:1). The signal of statistical difference are represented as follows indicated, *p < 0.05 CT versus OBS, **p < 0.05 CT versus OBexT and ***p < 0.05 OBS versus OBexT (n = 5 CT group; n = 6 OBS group; n = 6 OBexT group). One-way Anova statistical analysis was used, followed by Bonferroni post-test.

To assess a more accurate fatty acid profile in the sWAT, it was carried out the gas-chromatography technique coupled to mass-spectrometer to understand the modulation of the lipid profile of sWAT induced by obesity and short-term strength training. It was observed that obesity increased the amount of total saturated and monounsaturated fatty acid, while STST significantly decreased the saturated fatty acids parameters. On the other hand, the total polyunsaturated fatty acids were reduced by obesity and even more by the short-term strength training. When each fatty acid component was stratified, it was possible to observe that obesity increased palmitic (C16:0) and stearic (C18:0) fatty acids, while STST significantly decreased palmitic fatty acids. As expected, due to the high-fat diet, obese animals increased oleic fatty acid (C18:1), and it was not changed by the 7 days of strength training. The omega 6 and omega 3, such as linoleic (C18:2) and alpha-linolenic (C18:3) fatty acids, respectively, were reduced significantly by obesity and even more reduced by the short-term strength training. Other fatty acids, such as mirystic (C14:0), palmitoleic (C16:1), and eicosanoic (C20:1), were not changed by obese and exercise protocol (Fig. 3A–K).

### The effects of obesity and STST on the lipolysis pathway of sWAT

**Figure 4:**
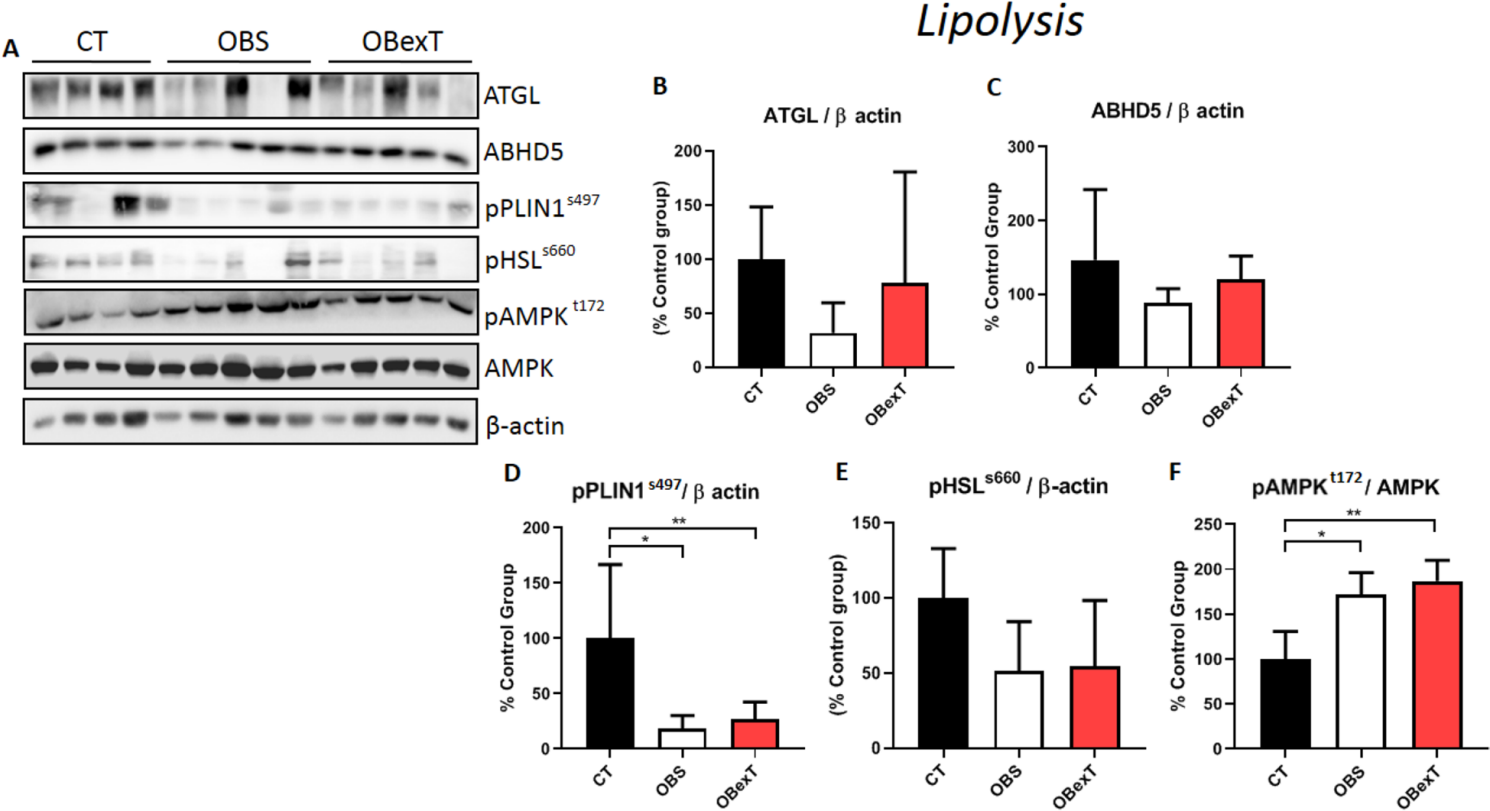
Total protein extract obtained from the subcutaneous adipose tissue was used for Western Blotting experiments. Uncut images can be viewed in the supplementary material. The membranes were cut at the specific height of the target protein. (**A**) The protein content of (**B**) ATGL, (**C**) ABHD5, (**D**) pPLIN1^ser497^, (**E**) pHSL^s660^ and (**F**) pAMPK^t172^. The signal of statistical difference are represented as follows, *p < 0.05 CT versus OBS, **p < 0.05 CT versus OBexT and ***p < 0.05 OBS versus OBexT (n = 4 CT group; n = 5 OBS group; n = 5 OBexT group). One-way Anova statistical analysis was used, followed by Tukey's post-test.

After the experimental period, the subcutaneous adipose tissue was removed to analyze the lipolysis pathways (Fig. 4A). Regarding lipolytic activities, it was possible to observe that both obesity and exercise groups did not alter the ATGL (Fig. 4B) and ABHD5 levels (Fig. 4C). However, when the phosphorylated *Perilipin1* (pPLIN1^ser497^) was evaluated, it was shown that obesity reduced pPLIN1^ser497^ levels and that the seven sessions of exercise were not effective in reversing this parameter (Fig. 4D). Also, when analyzing the pHSL^s660^ protein it was not possible to observe a change in expression levels in both the obese and the exercised group (Fig. 4E). Moreover, when the pAMPK^t172^ was analyzed, it was shown that obesity increased its levels and that the STST was not effective in reversing this parameter (Fig. 4F).

### The effects of obesity and STST on the lipogenesis pathway of sWAT

**Figure 5:**
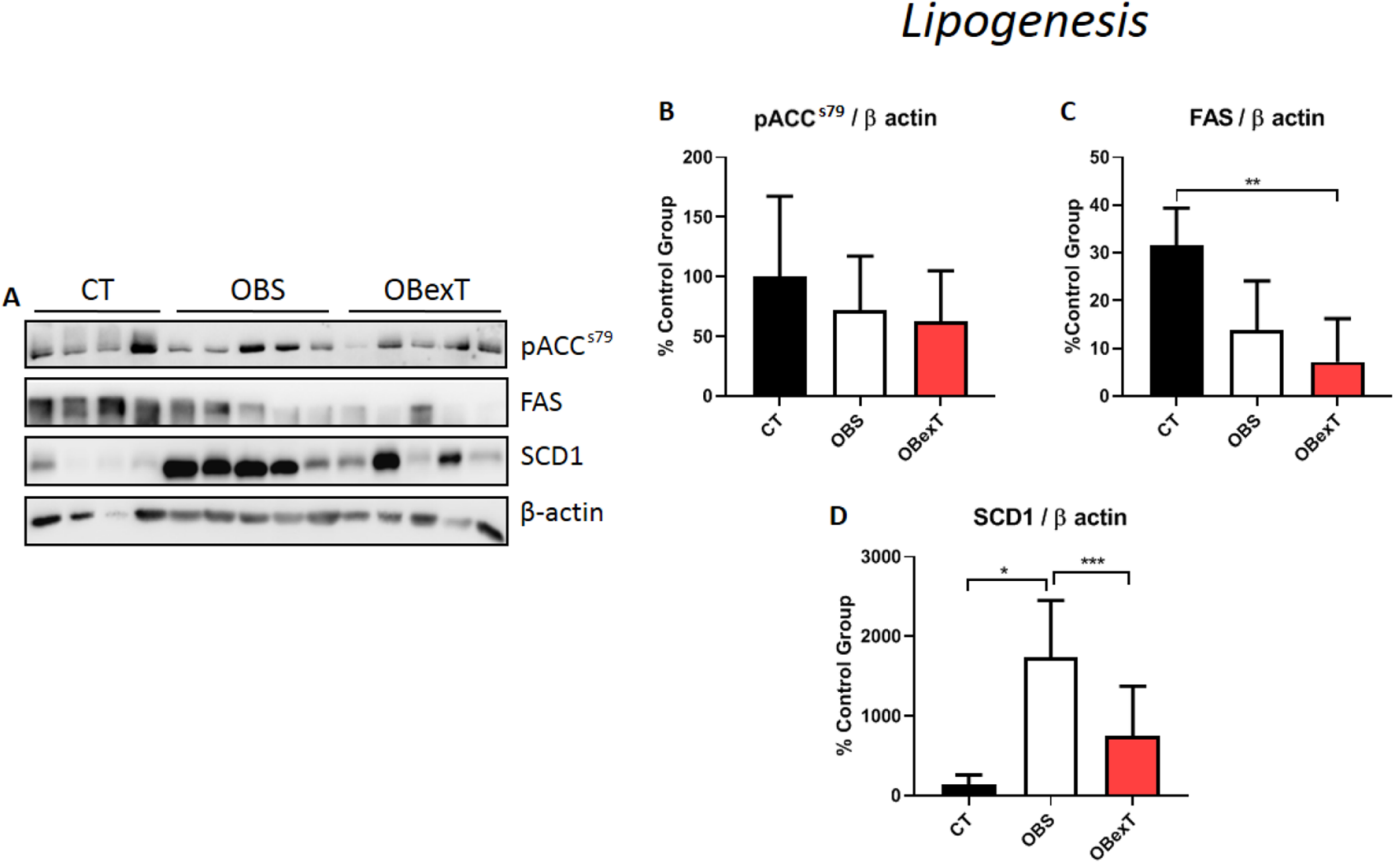
Total protein extract obtained from the sWAT was used for Western Blotting experiments. Uncut images can be viewed in the supplementary material. The membranes were cut at the specific height of the target protein. (**A**) The protein content of (**B**) pACC^ser79^, (**C**) FAS, and (**D**) SCD1. The signal of statistical difference are represented as follows, *p < 0.05 CT versus OBS, **p < 0.05 CT versus OBexT and ***p < 0.05 OBS versus OBexT (n = 4 CT group; n = 5 OBS group; n = 5 OBexT group). One-way Anova statistical analysis was used, followed by Tukey's post-test.

In Fig. 5, it was observed the influence of obesity and strength training on the proteins responsible for lipogenesis in sWAT (Fig. 5A). It was possible to verify that all groups did not show any difference in pACC^s79^ (Fig. 5B). Interestingly, obese trained animals decreased FAS levels when compared to the control group (Fig. 5C). However, when the levels of SCD1 were analyzed, it was observed that the sedentary obese group increased its levels when compared to lean control. Also, it was shown that after seven strength exercise sessions, the levels of SCD1 reached the control level, showing efficient effects of STST in reducing its levels (Fig. D).

### The effects of obesity and STST on browning of sWAT

**Figure 6:**
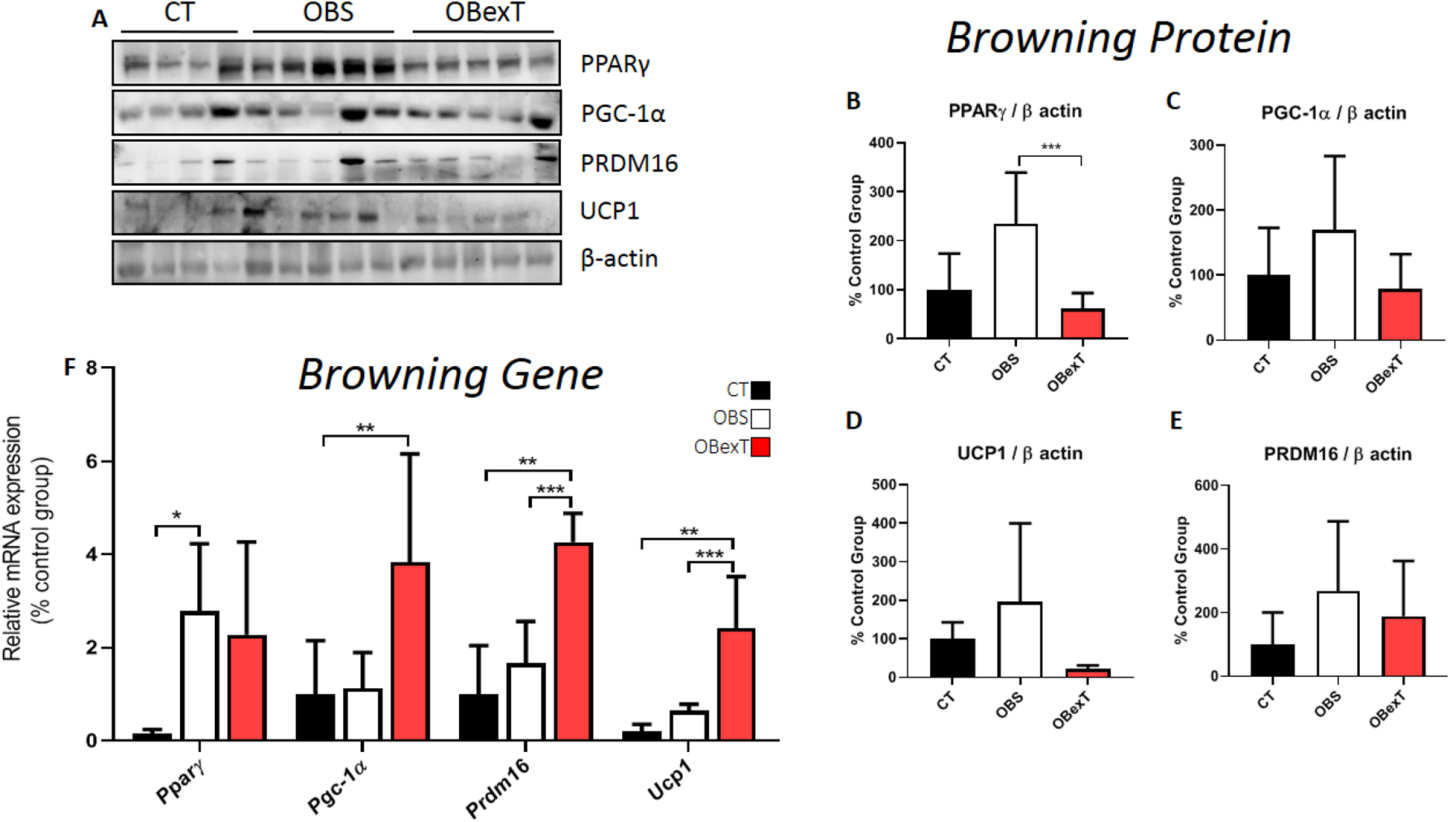
Total protein extract obtained from the subcutaneous adipose tissue was used for Western Blotting experiments. Uncut images can be viewed in the supplementary material. The membranes were cut at the specific height of the target protein. (**A**) The protein content of (**B**) PPARγ, (**C**) PGC-1α, (**D**) PRDM16, and (**E**) UCP1. The bars were relativized by the CT group (100%). In browning pathway, the signal of statistical difference are represented as follows *p < 0.05 CT versus OBS, **p < 0.05 CT versus OBexT and ***p < 0.05 OBS versus OBexT (n = 4 CT group; n = 5 OBS group; n = 5 OBexT group). In (**F**), the effect of strength training protocols on the induction of mRNA expression of genes involved in browning subcutaneous adipose tissue. Representative analysis in control, obese sedentary, and obese training swiss animals. Gene expression analysis of *Pparγ*, *Pgc-1α*, *Prdm16,* and *Ucp1*. In browning gene, the signal of statistical difference are represented as follows *p < 0.05 CT versus OBS, **p < 0.05 CT versus OBexT and ***p < 0.05 OBS versus OBexT (n = 7 CT group; n = 4 OBS group; n = 4 OBexT group). One-way Anova statistical analysis was used, followed by Tukey's post-test.

In addition, it was evaluated the genes and proteins responsible for the browning of sWAT (Fig. 6A). Firstly, it was observed the PPARγ metabolism. It was possible to verify that the sedentary obese group showed a significant increase in this gene compared to the control. However, after short-term strength training, it was not possible to identify any difference in gene levels compared to the obese sedentary group (Fig. 6F). When the total PPARγ was analyzed, it was observed that the obese group increased its levels when compared to the obese trained group, demonstrating the effect of 7 days of training reduced the expression levels (Fig. 6B). Secondly, it was observed the PGC-1*α* metabolism. It was possible to verify that the gene expression of *Pgc-1α* was increased in the obese animals after the STST only when compared to the control group (Fig. 6F). However, it was not possible to identify any difference between all groups in their total protein levels (Fig. 6C). Thirdly, it was possible to observe that the trained animals increased the *Prdm16* gene when compared to the control and obese groups (Fig. 6F). Subsequently, analyzing PRDM16 protein, no difference was found between all groups (Fig. 6D). Ultimately, the sedentary obese group showed no difference in *Ucp1* gene and protein content compared to the control group. However, when the trained animals were observed, this protocol increased the *Ucp1* gene compared to the control and obese groups (Fig. 6F). However, no differences were found in UCP1 protein levels for all groups (Fig. 6E).

## Discussion

The present study firstly aimed to assess whether seven strength training sessions promote sWAT browning. If so, secondly, to observe if this thermogenic adaptation can change lipid profile, increase lipolysis, and reduce the lipogenesis activity pathway in this tissue. At the end of this study, it was possible to observe that 7 days of strength training was able to change the fat tissue profile and moderately decrease the lipogenesis pathway. These findings were associated with the slightly beginning of the browning process in exercised obese animals.

It was identified that 14 weeks of obesity induction was able to increase fat mass, develop insulin resistance, and increase fasting glycemia and insulinemia. Corroborating our findings, Nakandakari and colleagues showed that 4 weeks of HFD in mice induces insulin resistance, body weight gain, and increased fasting glucose^29^. Moreover, it was possible to observe that obese exercised animals reduced fasting blood glucose, the area under the glycemic curve, and total cholesterol. In a study conducted by Pereira et al. fifteen sessions of strength exercise were able to reduce fasting glucose, hepatic triglyceride, and lipogenesis pathway in the liver of the obese mice^30^. For a chronic period, Marson and colleagues demonstrated that chronic aerobic, resistance, or combined training is associated with reduced fasting insulin in overweight or obese children, as mentioned in the present study^31^.

Subsequently, it was analyzed the effects of the high-fat diet on altering the lipid profile of fatty acids of subcutaneous adipose tissue. It sought to understand the composition of fatty acids in sWAT and the potential effect of strength training on altering this lipid profile.

The increment in the total saturated and monounsaturated fatty acid was expected in the sWAT of the groups treated with a high-fat diet, once the main source of lipids in the diet was from pork lard. We followed the American Institute of Nutrition^32^, in planning the modified high-fat diet, in accordance with Cintra et al.^33^. This diet contains ~ 21% of palmitic fatty acid and ~ 40% of oleic fatty acid in its composition. However, when compared to the content of these fatty acids in the commercial food for rodents, palmitic fatty acid reaches only 13 and ~ 27% of oleic fatty acid^33^. Then, these fatty acids are incorporated into adipose tissue depots, proportionally to founded in the diet. Interestingly, in the group performing the physical exercise protocol, the saturated fatty acids profile was changed in the subcutaneous adipose tissue, but not the monounsaturated fat profile, including its representants, palmitoleic and oleic fatty acids. We believe that palmitic fatty acid was driven to the β-oxidation process. This affirmative is corroborated by the partial reduction in the FAS and pACC protein contents but strongly correlated to a decrease in the SCD1 protein. With the absence of the SCD1 protein, the desaturation and elongation processes are compromised. It is certified once more due to the non-changes in the palmitoleic content (the first product after SCD1 action in palmitic fatty acid) or oleic fatty acid content.

The balance between synthesis and oxidation of fatty acids is a dynamic process, and during the exercise practicing, it is poorly understood. Besides, it could variates significantly in accordance with the experimental model adopted or tissue analyzed. For example, Rodrigues et al. (2017) evaluated the effects of voluntary physical exercise and aerobic training for 8 weeks in the composition of the fatty acid profile, in lean rats. They demonstrated that both the voluntary exercise protocol and aerobic training were not effective in reducing the levels of saturated fatty acids (C16:0 and C18:0) even reducing the visceral adiposity. However, both protocols were able to decrease the amount of unsaturated fatty acid (C16:1) in the epididymal adipose tissue^30^. It is known that there are significant differences among rat models (i.e. Fischer, Wistar, Sprague–Dawley) related to lipid metabolism or ability to retain or metabolize/mobilize fatty acids, under or not to HF-diet conditions^34^. Here, Swiss mice were chosen due to being a recognized model to accumulate fat mass and advance to obesity and its comorbidities, mimicking humans^35^. In parallel to our findings, Sutherland and colleagues, analyzing trained men to four months of high volume aerobic training (marathon), observed that the training load was able to reduce the levels of fatty acids palmitic (C16:0) and oleic (C18:1) in the subcutaneous adipose tissue when compared to sedentary individuals^32^. A reasonable explanation about fatty acids mobilization after exercise protocols could be related to the fatty acid desaturases (FADS1/2) or elongases (ELOV5/6) enzymes. While Rodrigues et al. (2017)^31^ observed the increased FADS1 and ELOV5 activity in adipose tissue after exercise training followed by reduced inflammatory markers (TNFα, IL6, and MCP1), Petridou et al., (2005) noticed a different activity of these enzymes in a tissue-specific manner. After exercise training, elongases were significantly higher and desaturases lower in adipose tissues (epididymal and subcutaneous) and muscle, while no differences were found in the liver^36^. The ability of exercise to induce modulation of FADS gene expression needs to be more explored as a very innovative field. Here, the exercise decreased the content of alpha-linolenic fatty acid (C18:3) an omega-3 species. In general, omega-3 fatty acids have been showing a protective effect in several conditions due to their anti-inflammatory properties^37^. However, as shown by Rodrigues et al., if the exercise increases FADS and ELOV enzymes, then it is possible that alpha-linolenic has a reduced content because it has been elonged and desaturated to another omega-3 species such as EPA (eicosapentaenoic acid [C20:5]) and DHA (docosahexaenoic acid [C22:6]). The role of individual fatty acids species modulation is an interesting field that needs more investigation. From a general perspective, we found a reduction of total saturated fatty acids induced by exercise. It could be understood as a desirable profile, such as noticed by Choi and colleagues, which found that the reduction of saturated fatty acids in the sWAT increased the insulin sensitivity in the tissue^1^. The lipolysis of sWAT can be controlled by AMPK. When phosphorylated, AMPK activates two lipases proteins, the hormone-sensitive lipase (HSL) and adipose triglyceride lipase (ATGL)^38^. Knowing this, Thirupathi et al. compared the effects of strength and aerobic training on the pAMPK in white adipose tissue. They showed that both exercise protocols were successful in increasing the activity of AMPK in trained animals when compared to the sedentary group^24^. Corroborating these findings, Higa and colleagues evaluated the effects of chronic aerobic exercise on the pAMPK levels in the visceral adipose tissue of obese mice. They showed that the exercise protocol was efficient in increasing the phosphorylation of AMPK^39^. On the other hand, Kurauti et al. observed that acute aerobic exercise was not effective in improving the AMPK phosphorylation in the subcutaneous white adipose tissue of obese animals^40^. In the present study, obese animals increased pAMPK levels in the subcutaneous adipose tissue, and the short-term training protocol was not sufficient to change this parameter. In our study, such changes may be observed if the training period was longer, as shown by the literature. Another limiting factor for not observing changes in pAMPK after exercise was the time of tissue collection after the exercise session. As demonstrated by Halling, immediately after the exercise session, the pAMPK is elevated in adipose tissue. However, 2 h after, its phosphorylation already reaches baseline values^41^.

After AMPK activation, lipolysis is induced by HSL phosphorylation^42^. In this regard, Wei and colleagues investigate the effects of dose-dependent induction of Isoproterenol (ISOP), ApoA-I, and HDL in 3T3-L1 adipocyte cells, it was observed that the combined incubation of ISOP + ApoA-I, or ISOP + HDL, was able to increase the expression of pHSL in adipocyte cells^43^. However, Lehnig et al*.* induced mice to 3 weeks of voluntary aerobic exercise and evaluated the effects of exercise on pHSL modulation in mesenteric adipose, after the period of physical exercise the authors did not observe an increase in the expression of pHSL in the mesenteric adipose tissue^44^. In contrast, Geng and colleagues induced Klb^f/f^ and Klb^adi^ mice, to aerobic training to evaluate the expression of pHSL in the epididymal adipose, at the end of 4 weeks it was possible to observe a significant increase in the expression of pHSL in eWAT of young mice^45^. However, in the present study, it was not possible to observe changes in the expression of pHSL in response to obesity and after an STST protocol.

Wohlers and colleagues evaluated the effects of acute aerobic exercise on the phosphorylation *Perilipin1* (PLIN1) in the visceral adipose tissue of obese mice. It was observed that the exercised animals did not increase the phosphorylation of PLIN1^46^. On the other hand, Ko et al. showed that after chronic aerobic exercise, the activation of PLIN1, HSL, and ATGL was increased^47^. Corroborating the findings of Ko et al. Americo and colleagues observed that 8 weeks of aerobic exercise was able to significantly reduce the diameter of adipocytes through the modulation of proteins PLIN1 and pHSL in obese animals^48^. In this sense, these findings suggest that the PLIN1 phosphorylation in the subcutaneous adipose tissue seems to be exercise time-dependent^47^. Thus, in the present study, it was observed that obese animals decreased the phosphorylation of PLIN1. Moreover, the short-term protocol was not able to revert this condition in sWAT. In this sense, it seems that the 7 days of STST may not be enough to increase the PLIN phosphorylation in subcutaneous adipose tissue.

Regulation of ATGL is known to be mediated by perilipin, which works by releasing ABHD5 protein for a physical binding with ATGL^49^. Therefore, Stephenson et al*.* evaluated the effects of short-term aerobic training on ATGL expression in epididymal adipose tissue of the old rats, after 6 weeks of physical effort it was possible to observe the increase in the ATGL in the epididymal adipose tissue of the runner’s rats^50^. In contrast, Nielsen and colleagues investigated the effects of short-term exercise on ATGL expression in sWAT in healthy men and concluded that moderate aerobic exercise was not able to promote the alteration of ATGL expression in subcutaneous adipose tissue^51^. However, Mendhan et al*.* observed an increase in ATGL gene expression in subcutaneous adipose tissue after 12 weeks of combined training in young adult women^52^. Interestingly, in the present study, it was not possible to observe changes in ATGL levels in response to obesity and after a strength training protocol.

Studies have demonstrated the influence of exercise on ABHD5 protein in subcutaneous adipose tissue. The ABHD5, when associated with ATGL, results in more significant fatty acid degradation in adipose tissue^53,54^. Stephenson et al. showed that 6 weeks of strength exercise was not enough to change the ABHD5 levels in visceral adipose tissue of obese animals. Moreover, Yao and colleagues showed that 12 weeks of aerobic training was not sufficient to alter the *Cgi-58* gene transcription (ABHD5) in the subcutaneous adipose tissue of overweight humans^55^. Corroborating these results, it was not possible to observe changes in ABHD5 levels in response to obesity and after an STST protocol.

Regarding the lipogenesis pathway, Pereira and colleagues evaluated the effects of short-term strength training (15 days of exercise) on ACC and FAS in the liver tissue of obese mice. They demonstrated that exercised obese animals were able to reduce the levels of fat accumulation and inflammation in the liver, due to the reduction of ACC and FAS activity when compared with the sedentary obese group^30^. Moreover, Chen et al. evaluated the effect of chronic moderate aerobic training on pACC levels in the subcutaneous adipose tissue of obese mice and observed that the exercise protocol was not effective in increasing the levels of pACC^56^. On the other hand, Arnt et al. evaluated patients with metabolic syndrome and submitted them to 16 weeks of exercise (high-intensity training "HIIT" vs. moderate exercise). After that, the white adipose tissue was evaluated, and the HIIT group presented a greater effect in decreasing post-training FAS levels when compared to the moderate training protocol group^57^. In the present study, seven sessions of strength training were not enough to observe a difference for the pACC and FAS proteins in comparison with the sedentary obese group. Based on other studies, it is believed that the time of intervention was not enough to activate these enzymes in the sWAT of obese trained animals^58,59^.

Lee and colleagues demonstrated that the high activity of the SCD1 enzyme in 3T3-L1 cells (adipose cells) is responsible for increasing the fatty acid synthesis process^60^. Pereira et al. showed that 15 days of strength exercise decreased the SCD1 levels in the liver of obese mice^30^. In parallel, Stotzer et al. evaluated the effects of chronic aerobic exercise on subcutaneous and mesenteric adipose tissue of obese rats. They observed that exercised animals decreased *Scd1* gene levels in mesenteric adipose tissue, but with no difference in the subcutaneous adipose tissue^61^. In the present study, it was demonstrated that obese animals increased SCD1 levels in sWAT and that only seven sessions of strength training were sufficient to reverse this parameter.

The browning process of white adipose occurs through the modulation of the PPARγ/PGC-1α/PRDM16 pathway that stimulates the synthesis of UCP1^62^. Reynolds et al*.* evaluated the effects of chronic aerobic exercise on the expression of *Pparγ* mRNA in epididymal adipose tissue of obese animals. The author demonstrated that the exercise protocol was not efficient in increasing the transcription of the *Pparγ* gene when compared with sedentary obese animals^51^. On the other hand, Petridou and colleagues showed that 8 weeks of voluntary running increased the expression of PPARγ in the subcutaneous adipose tissue of lean rats. In the present study, it was possible to observe that the sedentary obese group increased the levels of the *Pparγ* gene compared to the control group, but that the STST protocol was not effective in changing its levels in relation to the obese sedentary. However, when the total protein content of PPARγ was evaluated, obese groups increased their levels, and short-term strength training was responsible for decreasing the protein expression of PPARγ in the sWAT.

Stephenson and collaborators demonstrated that 6 weeks of aerobic exercise was not efficient in increasing the protein content of PGC-1α in sWAT of ovariectomized rats when compared to sedentary^50^. Norheim et al.^13^ analyzing healthy and overweight men submitted to 12 weeks of combined training (strength + endurance training), found no difference in the expression of the *Pgc-1α* gene in the subcutaneous adipose tissue. However, in the present study, it was possible to observe that short-term strength training was able to induce an increase in *Pgc-1α* gene transcription compared to the control group. However, it was not observed changes in the protein expression for PGC-1α for all groups.

The PRDM16 is the protein responsible for regulating the transcription of UCP1^64^. Lee et al.^65^ demonstrated that the PRDM16 collaborates with the browning of WAT in adipocyte cell culture (3T3-L1). Stanford and colleagues demonstrated that 11 days of aerobic running was able to increase the gene levels of *Prdm16* in the white adipose tissue of obese mice^23^. Still, Khalafi et al. evaluated the effects of 12 weeks of HIIT in obese male rats on PRDM16 expression. They demonstrated that the HIIT exercise was efficient in increasing the protein content of PRDM16 in subcutaneous adipose tissue^66^. In the present study, it was shown that STST was able to increase gene levels of *Prdm16* when compared to sedentary obese mice. However, seven strength training sessions were not able to increase the protein expression of PRM16. Probably, the time of intervention with training was not enough to find a difference in the protein content of PRM16 between the obese groups.

Finally, UCP1 is transcribed by the PPARγ/PGC-1α/PRMD16 complex^62^. It is a transmembrane protein located between the inner membrane and the mitochondrial matrix, responsible for modulating the proton gradient by removing H^+^ ions from the intramembranous space to the matrix and generating energy in the form of heat^67^. Schaalan and colleagues showed that 6 weeks of swimming training increased the expression of the *Ucp1* gene in the inguinal adipose tissue of obese rats^68^. On the other hand, after submitted obese mice to HIIT, Davis et al. did not observe any UCP1 modulation in the epididymal and retroperitoneal adipose tissue^69^. However, in the sWAT, Diaz and colleagues showed that 12 weeks of aerobic training increased *Ucp1* gene expression in the subcutaneous adipose tissue of overweight and obese humans (women and men)^70^. It was demonstrated that strength exercise was able to increase the *Ucp1* gene levels compared to the sedentary obese group. Interestingly, we did not observe any difference between the groups analyzing the UCP1 total protein content in sWAT. Our findings do not present a relationship between gene transcription and its protein expression. Thus, it is believed that the time of the strength training was not effective in promoting the necessary gene mRNA translation. Furthermore, the effects of strength training are still not well described in the literature as influencing changes in the sWAT genotype.

Therefore, we can conclude that the seven sessions of strength exercise were able to collaborate with the glucose homeostasis in obese mice. Also, this protocol decreased the levels of saturated, polyunsaturated, palmitic fatty acids and α-Linolenic fatty acids in sWAT. The 7 days strength exercise protocol did not change the lipolysis and slightly reduced the lipogenic pathways. Finally, our study demonstrated that the STST increased browning processes-related genes such as PGC-1α, PRDM16, and UCP1 in the sWAT. Interestingly, all these physiological and biomolecular mechanisms have been observed independently of changes in body weight. Thus, it is concluded that 7 days of strength training can be an effective strategy to initiate molecular changes and the browning process in sWAT.

## Methods

### Experimental animals

In the present study, it was used 8-week-old swiss mice from the UNICAMP Central Animals Facility (CEMIB). Animal experiments were carried out under Brazilian legislation on the scientific use of animals (Law No. 11,794, October 8, 2008) and were accepted by the Animal Ethics Committee (CEUA) of Biological Sciences, State University of Campinas, UNICAMP-Campinas-SP, No. 4773-1. In addition, our study followed the norms proposed by the ARRIVE guidelines. The animals were individually maintained in polyethylene cages in an enriched environment (PVC tubes were sawed into the medium yielding a 10 × 10 cm base and 5 cm high shelter) under controlled cycle conditions (12/12 h) with free access to water and conventional food, the light was switched on from 06:00 am to 06:00 pm, the temperature was controlled at 22 ± 2 °C, the relative humidity was 45–55%, and the noises were below of 85 decibels. Initially, the animals were divided into two groups, the chow diet (CT) and the high-fat diet (HFD). The animals from HFD were fed a high-fat diet (HFD, 60% of the calorie derived from lipids^33^) for 14 weeks, that were subdivided into two groups: obese sedentary (OBS) and obese strength training (OBexT).

### Seven days of training adaptation

Before starting the training protocol, the animals were adapted to exercise and its apparatus. The procedures were performed for 5 consecutive days. Before the first attempt to climb with the empty conic tube used to carry the load, the animal was kept inside the chamber at the top of the ladder for 60 s. In the first attempt of climbing, the animal was positioned on the stairs 15 cm away from the entrance of the chamber. For the second attempt, the animal was placed 25 cm away from the chamber. By the third attempt, the animal was placed at the base of the ladder, 70 cm away from the chamber. When the animal reached the chamber, an 60 s resting period was given. Attempts from the bottom of the ladder continued until the animal successfully completed three attempts without any stimulus.

### Maximal voluntary carrying capacity determination—MVCC

To control exercise intensity, the animals performed the test to determine the maximum voluntary carrying capacity (MVCC), proposed for rats^25,71^, and standardized for mice^30^. It consisted of an incremental test aiming to identify the maximum individual load with which the animal can perform a series of climbs of 70 cm. After the adaptation protocol, the animals rested for 1 day before starting the test. During the trial, the animals left the base of the ladder, and the attempt was considered successful when they reached the minimum distance proposed of 70 cm.

The initial series of the exercise were performed with 75% of the animal’s body weight overload. If the animal reached the desired height, increments of 5 g were added to the tube within each new attempt to climb until the animal could not complete the entire course, being considered exhaustion. In each successful attempt, the animal was removed from the ladder and placed in an individual cage resting for 5 min until the next attempt. The maximum load of the last successful attempt was considered the MVCC and was used to prescribe the individual loads during the experiment.

### Exercise protocol

**Figure 7:**
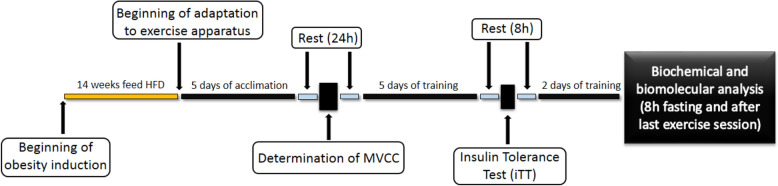
Experimental design. Schematic representation of the duration of the experiment.

Twenty-four h after the MVCC determination, the strength training protocol was initiated. The exercise sessions consisted of 20 climbing series, with an overload of 70% of the MVCC and with a rest interval of 60–90 s between sets. After completing a series, the animal was removed from the ladder and placed in an individual cage during rest time. The animals were exercised for 5 consecutive days. Then, 8 h after their last exercise session, they performed the insulin tolerance test and more 8 h of rest. After that, they performed more 2 days of exercise. Lastly, 8 h after their last exercise session, they were euthanized for tissue sample harvest and biomolecular analyses (Fig. 7).

### Intraperitoneal insulin tolerance test (ITT)

After a fasting period of 8 h and after the 5th physical exercise session, the animals were submitted to ITT to estimate glucose uptake capacity. Before starting the test, baseline blood glucose was assessed. Then the insulin (1U/kg body weight) was injected intraperitoneally (i.p.), and blood samples were collected at 5, 10, 15, 20, 25, and 30 min from the tail to determine blood glucose. Glucose levels were determined using a glucometer (Accu-Chek®; Roche Diagnostics, Jaguaré-SP, Brazil). Results were evaluated by determining areas under capillary glucose curves (AUC) by the trapezoidal method^72^ using Microsoft Excel®(Washington, USA).

### Adipose tissue extraction

After the ITT, all animals were submitted to two additional strength exercise session, and 8 h after the 7th exercise session, they were anesthetized via i.p. injection of chloral hydrate (300 mg/kg, ketamine, Parke-Davis, Ann Arbor, MI) and xylazine (30 mg/kg, Rompun, Leverkusen, Germany). After this, the corneal reflexes were verified and assured. After that, the blood was collected, and the subcutaneous adipose tissue was rapidly removed and snap-frozen in liquid nitrogen and stored at − 80 °C until analysis. During the experiments, the total number of animals analyzed varies according to the physical and material capacity of each equipment/ technique. The tissue was homogenized in extraction buffer (1% Triton-X 100, 100 mM Tris (pH 7.4), 100 mM sodium pyrophosphate, 100 mM sodium fluoride, 10 mM EDTA, 10 mM sodium vanadate, 2 mM PMSF and 0.1 mg of aprotinin/mL) at 4 °C with a TissueLyser II (QUIAGEN®, Hilden, Germany) operated at maximum speed for 240 s.

### Western blotting

The lysates were centrifuged (Eppendorf 5804R) at 12.851 g at 4 °C for 45 min to remove insoluble material, and the supernatant was used for the assay. The protein content was determined according to the bicinchoninic acid method^73^. The samples were applied to a polyacrylamide gel for separation by SDS-PAGE and transferred to nitrocellulose membranes. The membranes were cut at the specific height of the target protein. The membranes were blocked with 5% dry milk at room temperature for 1 h and then incubated with primary antibodies against the protein of interest ([Cell® (Danvers, MA, USA) UCP1#14,670, PPARγ#2435, SCD1#2794 s, β-actin#3700, pACCs79#3661, pHSLs660#4126, ATGL#2138], [Vala Sciences® (San Diego, CA, USA) pPLIN1s497#4855], [Abcam® (Cambridge, LON, UK) PRDM16#ab106410], [Santa Cruz® (Dallas, TX, USA) PGC-1α#sc13067, pAMPKt172#sc33524, AMPK#sc25792, FAS#sc48357], [Proteintech® (Rosemont, IL, USA) ABHD5#12,201–1-ap]). After that, a specific secondary antibody was used, according to the primary antibody. The specific bands were labeled by chemiluminescence, and visualization was performed by photo documentation system in G:box (Syngene, Bangalore, IND). The bands were quantified using the software UN-SCAN-IT gel 6.1 (Orem, UT, USA).

### Spectrophotometry

Blood serum was obtained through the collection with a collecting tube after the animal was decapitated, and the levels of triglycerides, cholesterol, glucose, and HDL were determined according to the manufacturer's instructions using a commercial kit (Laborlab, Guarulhos, São Paulo, Brasil) and using (Biotek Gen5™, Winooski, VT, USA) LDL determination by manufacturer's instructions method and Friedewald formula^74^.

### Quantitative real-Time PCR

Total RNA was isolated using TRIzol reagent (Invitrogen, Grand Island, NY, USA). An amount of 2 µg total RNA was used as a template for cDNA synthesis using the SuperScript® III First Chain Synthesis System (Invitrogen, Carlsbad, CA, USA). Real-time PCR reactions were performed using 150 ng cDNA, 300 nM primers (Exxtend®, Paulínia-SP, Brazil), and SYBR® Select Master Mix (Applied Biosystem, Warrington, UK). Thermocycling parameters were: 10 min at 95 °C, followed by 40 cycles of 15 s at 95 °C, 30 s at 60 °C, and 30 s at 72 °C. The relative expression of mRNAs was determined after normalization with *Ywhaz* using the ΔΔCt method. Each primer set was designed to recognize unique regions of gene sequences, according to Table 1.

**Table 1:** This table represents the browning pathway gene sequence used for qPCR analysis.

	Forward	Reverse
*Pparg*	5′GTACTGTCGGTTTCAGAAGTGCC3′	5′ATCTCCGCCAACAGCTTCTCCT3′
*Pgc-1(alpha)*	5′GAATCAAGCCACTACAGACACCG3′	5′CATCCCTCTTGAGCCTTTCGTG3′
*Prdm16*	5′ATCCACAGCACGGTGAAGCCAT3′	5′ACATCTGCCCACAGTCCTTGCA3′
*Ucp1*	5′GCTTTGCCTCACTCAGGATTGG3′	5′CCAATGAACACTGCCACACCTC3′
*Ywhaz*	5′GAACTCCCCAGAGAAAGCCT3′	5′CCGATGTCCACAATGTCAAGT3′

### Adipose tissue lipid profile analyses

Lipids from subcutaneous adipose tissue were extracted following the proposed by Folch et al.^75^. About 50 mg of tissue was added to 1 mL of chloroform: methanol (2:1 v/v). The samples were homogenized in the TissueLyser (QIAGEN® Hilden, Germany), centrifuged at 13.000 × *g* for 2 min, and then the supernatant was collected. The esterification of the lipid fraction was performed according to the method adopted by Shirai et al.^76^. The fatty acids methyl esters were analyzed with a gas chromatograph-mass spectrometer (Shimadzu® GCMS-QP2010 Ultra, Nakagyo-ku, Kyoto, Japan) and a fused-silica capillary Stabilwax column (Restek Corporation, Bellefonte, PA, USA) with dimensions of 30 m × 0.25 mm internal diameter coated with a 0.25-μm thick layer of polyethylene glycol. High-grade pure helium (He) was used as the carrier gas with a constant flow rate of 1.0 mL/min. Using an automatic injector (AOC-20i), sample volumes of 1 μL were injected at 250 °C using a 20: 1 split ratio. The injector temperature was maintained at 250 °C, while the oven heating schedule started at 80 °C, with heating speed programmed in a ramp from 5 °C/min to 175 °C, followed by a heating rate of 3 °C /min until 230 °C, where the temperature was maintained for 20 min^77^. Mass conditions were as follows: ionization voltage, 70 eV; ion source temperature, 200 °C; full scan mode in the 35–500 mass range with 0.2 s/scan velocity.

### Statistical analysis

Before all the analysis, the Gaussian distribution of the data was assessed by the Shapiro–Wilk normality test. The *Anova one-way* with Bonferroni multiple comparison tests was performed for total cholesterol, triglycerides, HDL, and adipose tissue profile. *Anova one-way* with Tukey’s multiple comparison test was performed for body weight, fasting insulin, fasting blood glucose, RT qPCR, and Western Blot data analysis. *Anova two-way* with Tukey’s multiple comparison test was performed to compare ipITT glycemic curve time points. All results were expressed as mean ± standard deviation (S.D.) and the statistical significance level adopted was *p* < 0.05 for all analyses. The statistical analysis and the construction of the graphs were performed using the software GraphPad Prism7® (San Diego, CA, USA).

## Supplementary Information

Supplementary Information 1.

Supplementary Information 2.
