# Supplementary material for: Strength training alters the tissue fatty acids profile and slightly improves the thermogenic pathway in the adipose tissue of obese mice
Source: Sci Rep. 2022 Apr 28;12:6913. doi: 10.1038/s41598-022-10688-w (PMC9050661; doi:10.1038/s41598-022-10688-w)
Supplement: Supplementary file 1 — Supplementary Information 1. [file 41598_2022_10688_MOESM1_ESM.pdf]

Strength training alters the tissue fatty acids profile and slightly improves the thermogenic pathway in the adipose tissue of obese mice

Diego Gomes de Melo; Chadi Pellegrini Anaruma; Kellen Cristina da Cruz Rodrigues; Rodrigo Martins Pereira; Thais Dantis Pereira de Campos; Raphael Santos Canciglieri; Camila Oliveira Ramos; Dennys Esper Cintra; Eduardo Rochete Ropelle; Adelino Sanchez Ramos da Silva; José Rodrigo Pauli; Leandro Pereira de Moura*.

Additional Material

Strength training alters the tissue fatty acids profile and slightly improves the thermogenic pathway in the adipose tissue of obese mice

The additional material corresponds to the RT-PCR analysis of the genes involved in the browning process of the subcutaneous adipose tissue. The data that were removed correspond to outliers that were identified after the statistical test (when using the ROUT method or when using the GRUBBS analysis).

BEFORE ANALISYS

Group A	Group B	Group C
Data Set-A	*Pparγ*	Data Set-C
0.20	2.64	1.92
0.07	1.67	2.14
0.13	4.87	4.93
0.14	1.94	0.10
6.11		
0.07		
0.30		

AFTER ANALISYS

	Group A	Group B	Group C
	Data Set-A	*Pparγ*	Data Set-C
1	0.20	2.64	1.92
2	0.07	1.67	2.14
3	0.13	4.87	4.93
4	0.14	1.94	0.10
5	*6.11**		
6	0.07		
7	0.30		

Cleaned data | Outliers | Summary

	A	B	C
		Pparγ	
1	0.200	2.640	1.920
2	0.070	1.670	2.140
3	0.130	4.870	4.930
4	0.140	1.940	0.100
5			
6	0.070		
7	0.300		

Cleaned data | Outliers | Summary

		A	B
			Pparγ
1	#5	6.110	

Cleaned data | Outliers | Summary

Identify outliers Summary	A	B	C
		Pparγ	
1 Method			
2 ROUT (Q = 1%)			
3			
4 Number of points			
5 # Y values analyzed	7	4	4
6 Outliers	1	0	0

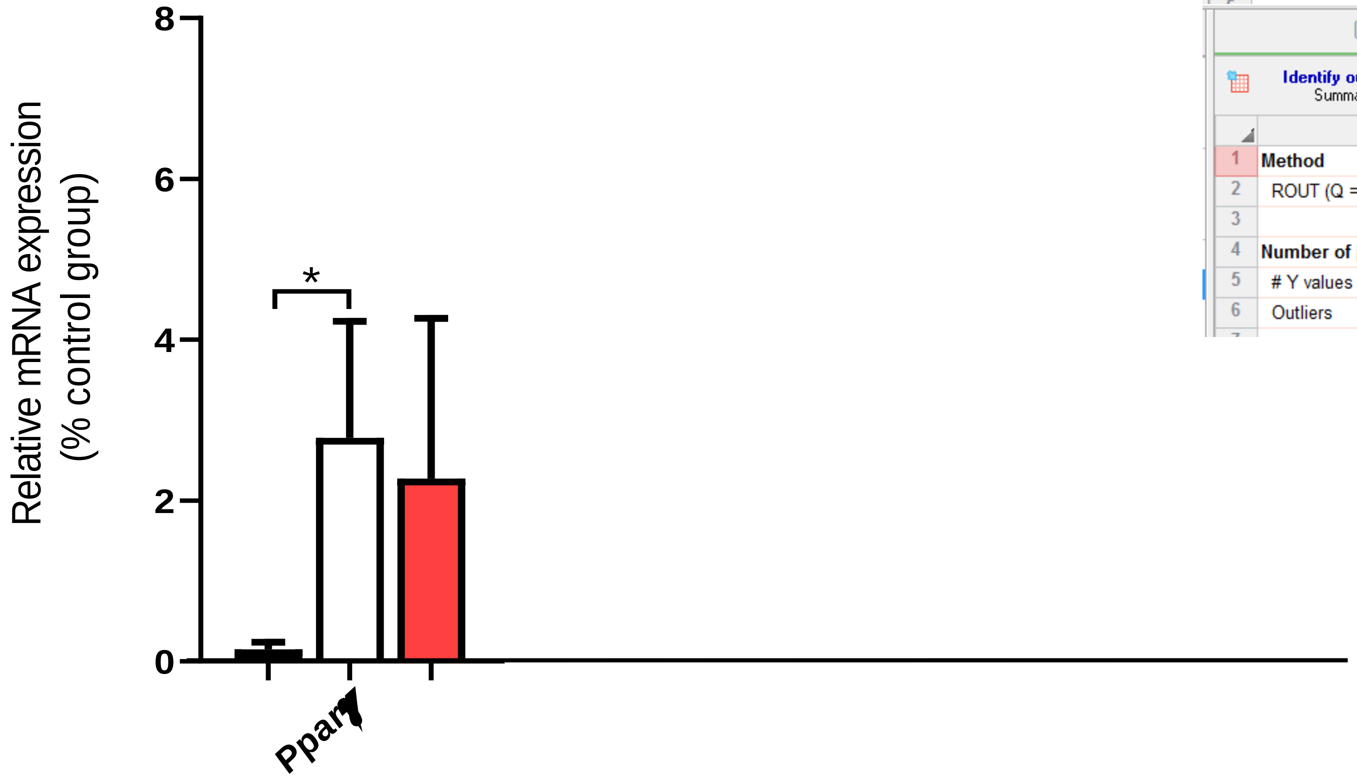

IDENTIFY OUTLIERS OF PPARgamma

BEFORE ANALISYS

	Group E	Group F	Group G
	Data Set-E	*Pgc-1α*	Data Set-G
1	0.38	0.70	0.70
2	0.21	1.87	5.89
3	3.13	1.66	3.48
4	2.06	0.25	5.27
5	0.39		
6	0.73		
7	0.10		

AFTER ANALISYS

	Group E	Group F	Group G
	Data Set-E	*Pgc-1α*	Data Set-G
1	0.38	0.70	0.70
2	0.21	1.87	5.89
3	3.13	1.66	3.48
4	2.06	0.25	5.27
5	0.39		
6	0.73		
7	0.10		

Cleaned data | Outliers | Summary

	A	B	C
		Pgc-1α	
1	0.380	0.700	0.700
2	0.210	1.870	5.890
3	3.130	1.660	3.480
4	2.060	0.250	5.270
5	0.390		
6	0.730		
7	0.100		

Cleaned data | Outliers | Summary

	A	B	C
		Pgc-1α	
1			
2			
3			
4			
5			
6			
7			

Cleaned data | Outliers | Summary

Identify outliers Summary

		A	B	C
			Pgc-1α	
1	**Method**			
2	ROUT (Q = 1%)			
3				
4	**Number of points**			
5	# Y values analyzed	7	4	4
6	Outliers	0	0	0

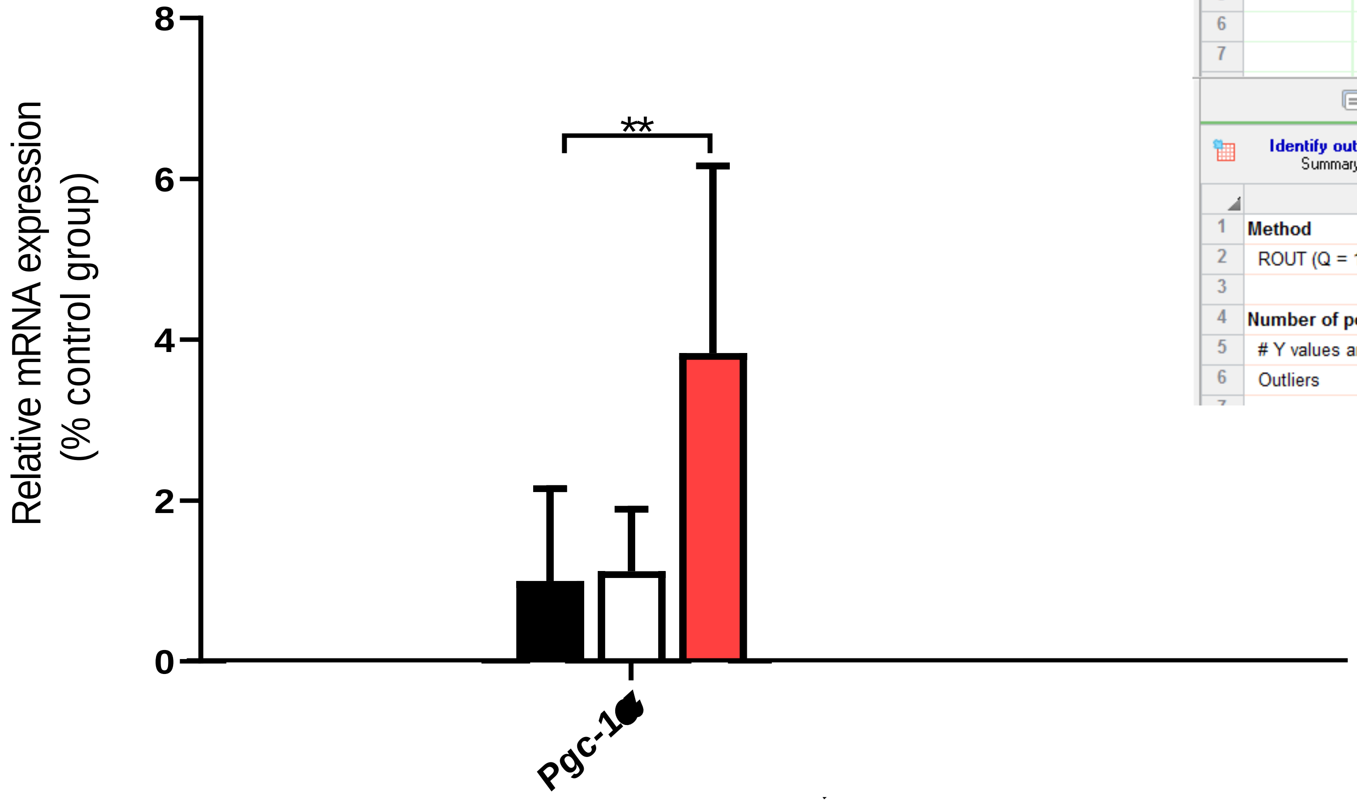

IDENTIFY OUTLIERS OF PGC-1alpha

BEFORE ANALISYS

	Group I	Group J	Group K
	Data Set-I	*Prdm16*	Data Set-K
1	0.33	1.44	4.26
2	2.72	2.65	5.14
3	2.07	11.78	3.81
4	0.12	0.89	3.85
5	1.25		
6	0.28		
7	0.24		
8			

AFTER ANALISYS

	Group I	Group J	Group K
	Data Set-I	*Prdm16*	Data Set-K
1	0.33	1.44	4.26
2	2.72	2.65	5.14
3	2.07	*11.78**	3.81
4	0.12	0.89	3.85
5	1.25		
6	0.28		
7	0.24		
8			

Cleaned data | Outliers | Summary

	A	B	C
		Prdm16	
1	0.330	1.440	4.260
2	2.720	2.650	5.140
3	2.070		3.810
4	0.120	0.890	3.850
5	1.250		
6	0.280		
7	0.240		
8			

Cleaned data | Outliers | Summary

		A	B	C
			Prdm16	
1	#3		11.780	
2				
3				
4				
5				
6				
7				

Cleaned data | Outliers | Summary

Identify outliers Summary	A	B	C
		Prdm16	
1 **Method**			
2 Grubbs (Alpha = 0.05)	G = 1.653	G = 1.484	G = 1.416
3			
4 **Number of points**			
5 # Y values analyzed	7	4	4
6 Outliers	0	1	0
7			

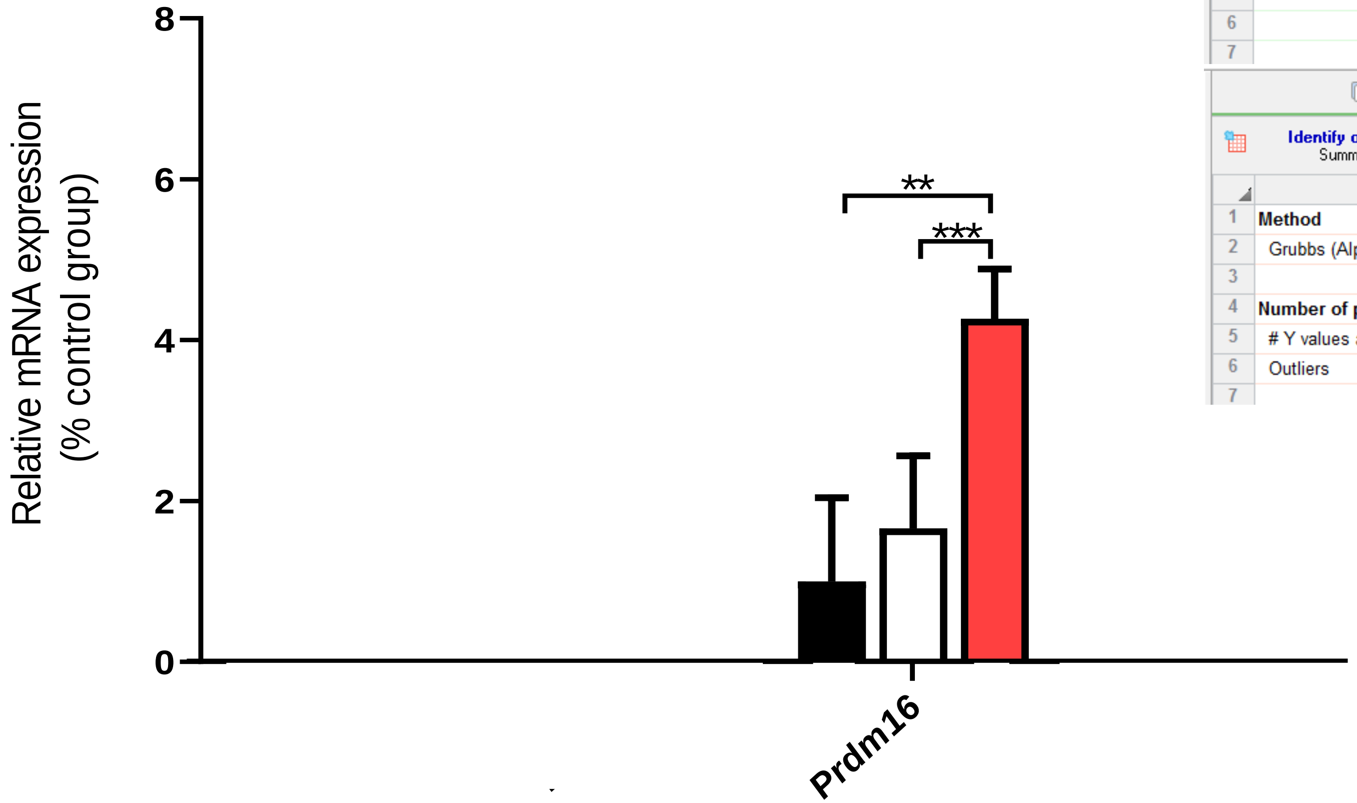

IDENTIFY OUTLIERS OF PRDM16

BEFORE ANALISYS

	Group M	Group N	Group O
	Data Set-M	*Ucp1*	Data Set-O
1	2.71	0.61	1.38
2	0.05	0.64	3.58
3	3.31	0.49	2.29
4	0.01	0.83	46.90
5	0.33		
6	0.28		
7	0.31		
8			

AFTER ANALISYS 1

	Group M	Group N	Group O
	Data Set-M	*Ucp1*	Data Set-O
1	2.71	0.61	1.38
2	0.05	0.64	3.58
3	*3.31**	0.49	2.29
4	0.01	0.83	*46.90**
5	0.33		
6	0.28		
7	0.31		
8			

Cleaned data | Outliers | Summary

	A	B	C
		Ucp1	
1	2.710	0.610	1.380
2	0.050	0.640	3.580
3		0.490	2.290
4	0.010	0.830	
5	0.330		
6	0.280		
7	0.310		
8			

Cleaned data | Outliers | Summary

		A	B	C
			Ucp1	
1	#3	3.310		
2	#4			46.900
3				
4				
5				
6				
7				
8				

Cleaned data | Outliers | Summary

	Identify outliers Summary	A	B	C
			Ucp1	
1	**Method**			
2	ROUT (Q = 1%)			
3				
4	**Number of points**			
5	# Y values analyzed	7	4	4
6	Outliers	1	0	1
7				
8				

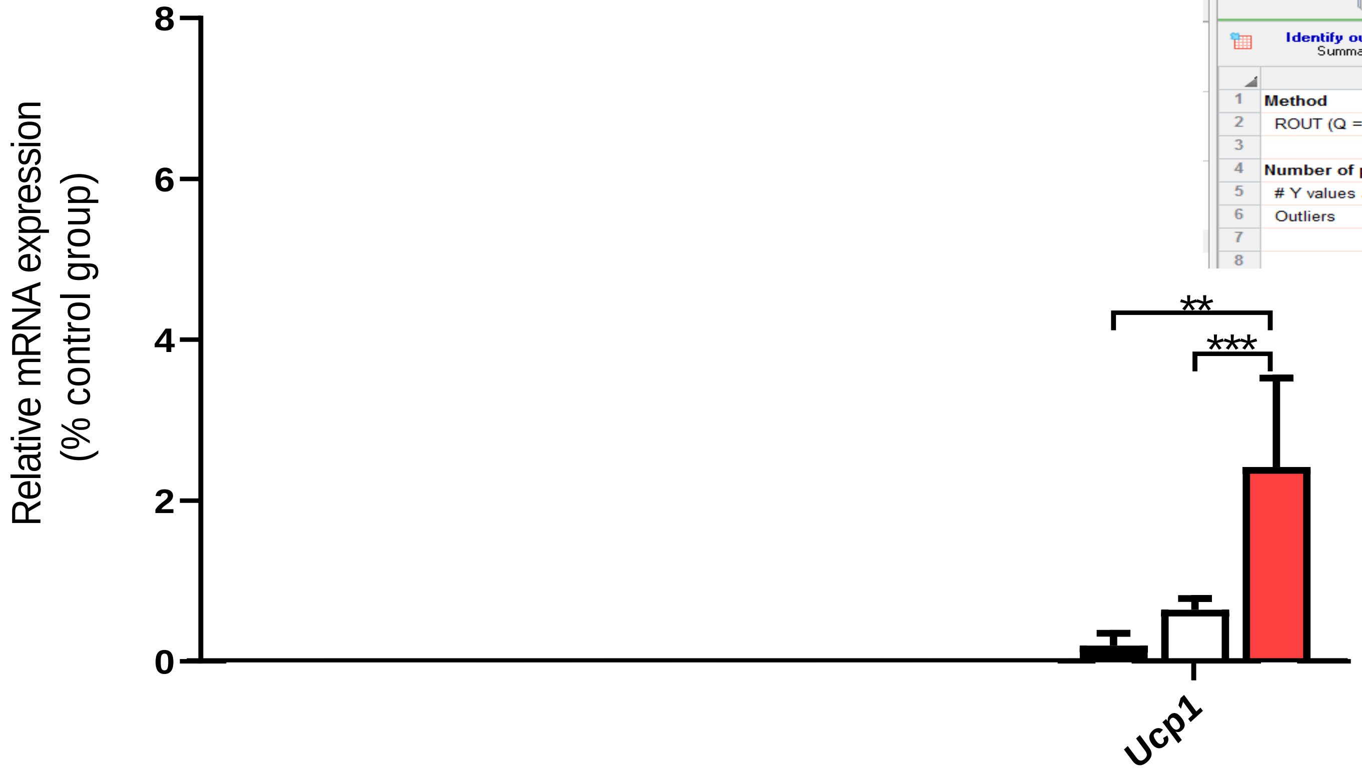

IDENTIFY OUTLIERS OF UCP1

BEFORE ANALISYS

	Group M	Group N	Group O
	Data Set-M	*Ucp1*	Data Set-O
1	2.71	0.61	1.38
2	0.05	0.64	3.58
3	3.31	0.49	2.29
4	0.01	0.83	46.90
5	0.33		
6	0.28		
7	0.31		
8			

AFTER ANALISYS 2

	Group M	Group N	Group O
	Data Set-M	*Ucp1*	Data Set-O
1	*2.71**	0.61	1.38
2	0.05	0.64	3.58
3	*3.31**	0.49	2.29
4	0.01	0.83	*46.90**
5	0.33		
6	0.28		
7	0.31		
8			

Cleaned data | Outliers | Summary

	A	B	C
		Ucp1	
1		0.610	1.380
2	0.050	0.640	3.580
3		0.490	2.290
4	0.010	0.830	
5	0.330		
6	0.280		
7	0.310		
8			

Cleaned data | Outliers | Summary

		A	B
			Ucp1
1	#1	2.710	
2			
3			
4			
5			
6			
7			
8			

Cleaned data | Outliers | Summary

	Identify outliers Summary	A	B	C
			Ucp1	
1	**Method**			
2	ROUT (Q = 1%)			
3				
4	**Number of points**			
5	# Y values analyzed	6	4	3
6	Outliers	1	0	0
7				
8				

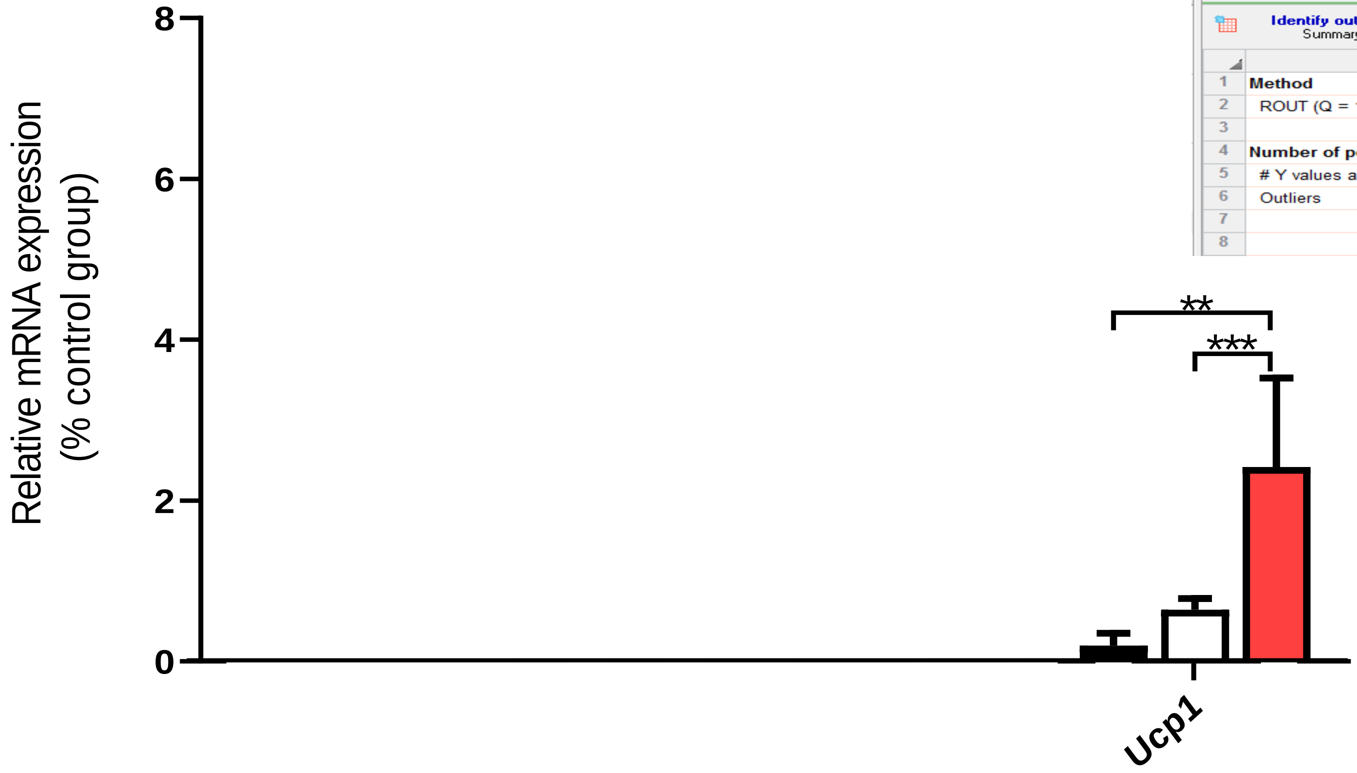

IDENTIFY OUTLIERS OF UCP1
