# Supplementary material for: Strength training alters the tissue fatty acids profile and slightly improves the thermogenic pathway in the adipose tissue of obese mice
Source: Sci Rep. 2022 Apr 28;12:6913. doi: 10.1038/s41598-022-10688-w (PMC9050661; doi:10.1038/s41598-022-10688-w)
Supplement: Supplementary file 2 — Supplementary Information 2. [file 41598_2022_10688_MOESM2_ESM.pdf]

Strength training alters the tissue fatty acids profile and slightly improves the thermogenic pathway in the adipose tissue of obese mice

Diego Gomes de Melo; Chadi Pellegrini Anaruma; Kellen Cristina da Cruz Rodrigues; Rodrigo Martins Pereira; Thais Dantis Pereira de Campos; Raphael Santos Canciglieri; Camila Oliveira Ramos; Dennys Esper Cintra; Eduardo Rochete Ropelle; Adelino Sanchez Ramos da Silva; José Rodrigo Pauli; Leandro Pereira de Moura*.

Supplementary Material

Fig. 1A

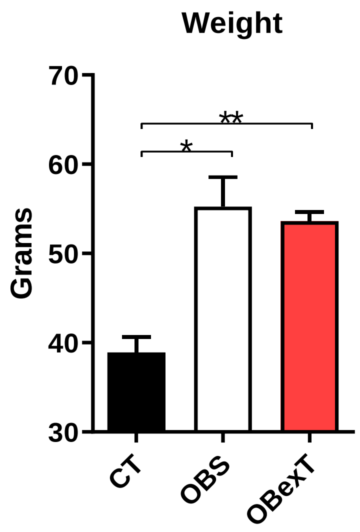

CT	OBS	OBexT
37.1	47.7	56.7
36.6	53.6	50.5
43.9	49.8	52.8
38.1	59.0	55.1
	66.1	52.9

Tukey's multiple comparisons test	Mean Diff.	95.00% CI of diff.	Significant?	Summary	Adjusted P Value	
CT vs. OBS	-16.32	-25.42 to -7.207	Yes	**	0.0014	A-B
CT vs. OBexT	-14.68	-23.78 to -5.567	Yes	**	0.0030	A-C
OBS vs. OBexT	1.640	-6.947 to 10.23	No	ns	0.8653	B-C

Test details	Mean 1	Mean 2	Mean Diff.	SE of diff.	n1	n2	q	DF
CT vs. OBS	38.93	55.24	-16.32	3.372	4	5	6.842	11
CT vs. OBexT	38.93	53.60	-14.68	3.372	4	5	6.154	11
OBS vs. OBexT	55.24	53.60	1.640	3.179	5	5	0.7295	11

Fig. 1B

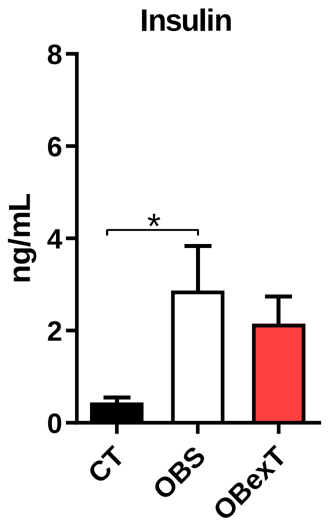

CT	OBS	OBexT
0.77321	0.80476	0.95813
0.73841	0.49831	4.48792
0.15697	2.56716	3.26488
0.32984	6.81233	0.82163
0.22986	2.20164	2.00792
0.44753	4.32450	1.36124

Tukey's multiple comparisor	Mean Diff.	95.00% CI of diff.	Significant?	Summary	Adjusted P Value	
CT vs. OBS	-2.422	-4.839 to -0.005315	Yes	*	0.0495	A-B
CT vs. OBexT	-1.704	-4.121 to 0.7125	No	ns	0.1932	A-C
OBS vs. OBexT	0.7178	-1.699 to 3.135	No	ns	0.7256	B-C

Test details	Mean 1	Mean 2	Mean Diff.	SE of diff.	n1	n2	q	DF
CT vs. OBS	0.4460	2.868	-2.422	0.9305	6	6	3.681	15
CT vs. OBexT	0.4460	2.150	-1.704	0.9305	6	6	2.590	15
OBS vs. OBexT	2.868	2.150	0.7178	0.9305	6	6	1.091	15

Fig. 1C

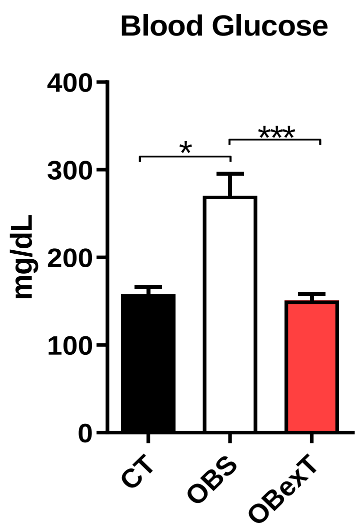

CT	OBS	OBexT
152.0	333.0	125.0
179.0	313.0	154.0
163.0	282.0	157.0
138.0	212.0	144.0
	212.0	174.0

Tukey's multiple comparisons test	Mean Diff.	95.00% CI of diff.	Significant?	Summary	Adjusted P Value	
CT vs. OBS	-112.4	-179.1 to -45.74	Yes	**	0.0022	A-B
CT vs. OBexT	7.200	-59.46 to 73.86	No	ns	0.9544	A-C
OBS vs. OBexT	119.6	56.75 to 182.4	Yes	***	0.0009	B-C

Test details	Mean 1	Mean 2	Mean Diff.	SE of diff.	n1	n2	q	DF
CT vs. OBS	158.0	270.4	-112.4	24.68	4	5	6.441	11
CT vs. OBexT	158.0	150.8	7.200	24.68	4	5	0.4126	11
OBS vs. OBexT	270.4	150.8	119.6	23.27	5	5	7.269	11

Fig. 1D

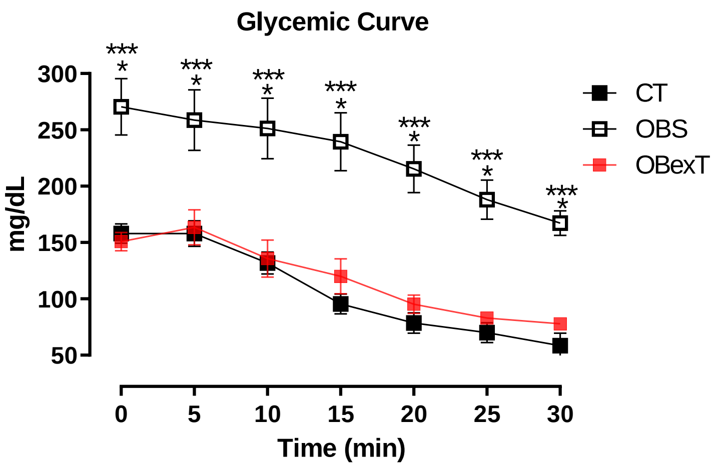

Min	CT					OBS					OBexT				
X	A:Y1	A:Y2	A:Y3	A:Y4	A:Y5	B:Y1	B:Y2	B:Y3	B:Y4	B:Y5	C:Y1	C:Y2	C:Y3	C:Y4	C:Y5
0	152	179	163	138		333	313	282	212	212	125	154	157	144	174
5	167	163	177	125		344	282	261	187	219	120	152	196	148	202
10	139	130	152	106		327	288	258	183	200	96	102	167	137	177
15	110	100	102	70		305	279	251	167	195	80	92	143	122	163
20	91	81	90	52		266	261	203	158	189	67	90	108	98	113
25	86	66	81	47		241	206	180	135	178	66	84	96	78	91
30	85	52	65	32		195	189	158	135	159	62	80	88	72	87

Tukey's multiple comparisons test	Mean Diff.	95.00% CI of diff.	Significant?	Summary	Adjusted P Value
Row 1					
CT vs. OBS	-112.4	-199.6 to -25.16	Yes	*	0.0196
CT vs. OBexT	7.200	-28.07 to 42.47	No	ns	0.8205
OBS vs. OBexT	119.6	32.32 to 206.9	Yes	*	0.0156
Row 2					
CT vs. OBS	-100.6	-193.8 to -7.420	Yes	*	0.0377
CT vs. OBexT	-5.600	-62.58 to 51.38	No	ns	0.9546
OBS vs. OBexT	95.00	1.212 to 188.8	Yes	*	0.0476
Row 3					
CT vs. OBS	-119.5	-212.4 to -26.55	Yes	*	0.0196
CT vs. OBexT	-4.050	-61.89 to 53.79	No	ns	0.9756
OBS vs. OBexT	115.4	21.41 to 209.4	Yes	*	0.0209
Row 4					
CT vs. OBS	-143.9	-233.0 to -54.84	Yes	**	0.0078
CT vs. OBexT	-24.50	-78.60 to 29.60	No	ns	0.4065
OBS vs. OBexT	119.4	29.60 to 209.2	Yes	*	0.0145
Row 5					
CT vs. OBS	-136.9	-209.3 to -64.46	Yes	**	0.0034
CT vs. OBexT	-16.70	-53.22 to 19.82	No	ns	0.4082
OBS vs. OBexT	120.2	47.93 to 192.5	Yes	**	0.0065
Row 6					
CT vs. OBS	-118.0	-178.7 to -57.35	Yes	**	0.0026
CT vs. OBexT	-13.00	-46.13 to 20.13	No	ns	0.4664
OBS vs. OBexT	105.0	44.33 to 165.7	Yes	**	0.0062
Row 7					
CT vs. OBS	-108.7	-155.2 to -62.25	Yes	***	0.0006
CT vs. OBexT	-19.30	-61.93 to 23.33	No	ns	0.3468
OBS vs. OBexT	89.40	51.39 to 127.4	Yes	**	0.0011

Fig. 1E

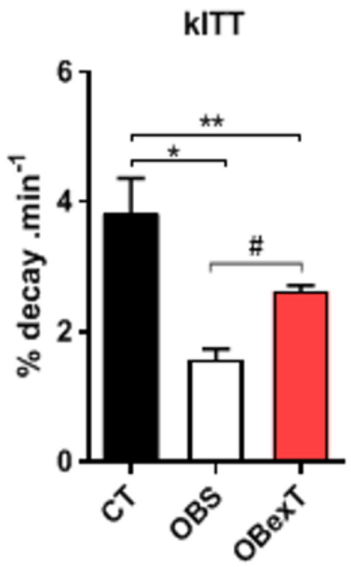

CT	OBS	OBexT
2.496139466	1.802558646	2.613476879
4.278358911	1.599907431	2.340048922
3.461114856	1.943440595	2.571516067
5.037914996	1.537495204	2.639615631
	0.952994948	2.945014170

Tukey's multiple comparisons test	Mean Diff.	95.00% CI of diff.	Significant?	Summary	Adjusted P Value	
CT vs. OBS	2.251	1.113 to 3.389	Yes	***	0.0006	A-B
CT vs. OBexT	1.196	0.05871 to 2.334	Yes	*	0.0394	A-C
OBS vs. OBexT	-1.055	-2.127 to 0.01801	No	ns	0.0540	B-C

Test details	Mean 1	Mean 2	Mean Diff.	SE of diff.	n1	n2	q	DF
CT vs. OBS	3.818	1.567	2.251	0.4213	4	5	7.557	11
CT vs. OBexT	3.818	2.622	1.196	0.4213	4	5	4.017	11
OBS vs. OBexT	1.567	2.622	-1.055	0.3972	5	5	3.755	11

Fig. 1F

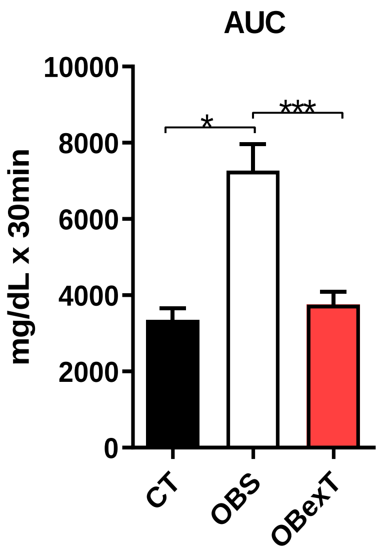

CT	OBS	OBexT
3770.00	9222.50	2767.50
3407.50	8307.50	3385.00
3742.50	7260.00	4382.50
2505.00	5355.00	3635.00
	6230.00	4600.00

Tukey's multiple comparisons test	Mean Diff.	95.00% CI of diff.	Significant?	Summary	Adjusted P Value	
CT vs. OBS	-3919	-5882 to -1955	Yes	***	0.0006	A-B
CT vs. OBexT	-397.8	-2361 to 1566	No	ns	0.8500	A-C
OBS vs. OBexT	3521	1670 to 5372	Yes	***	0.0009	B-C

Test details	Mean 1	Mean 2	Mean Diff.	SE of diff.	n1	n2	q	DF
CT vs. OBS	3356	7275	-3919	727.0	4	5	7.623	11
CT vs. OBexT	3356	3754	-397.8	727.0	4	5	0.7737	11
OBS vs. OBexT	7275	3754	3521	685.4	5	5	7.265	11

Fig. 2A

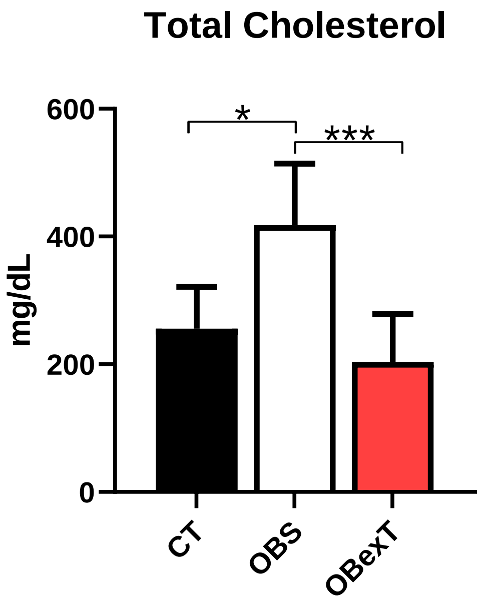

CT	OBS	OBexT
171.43	507.936508	266.666667
339.68	363.492063	133.333333
211.11	560.317460	161.904762
286.51	352.380952	174.603175
269.84	315.873016	161.904762
	407.936508	325.396825

Bonferroni's multiple comparisons test	Mean Diff.	95.00% CI of diff.	Significant?	Summary	Adjusted P Value			
CT vs. OBS	-162.3	-295.3 to -29.23	Yes	*	0.0153	A-B		
CT vs. OBexT	51.75	-81.30 to 184.8	No	ns	0.9252	A-C		
OBS vs. OBexT	214.0	87.16 to 340.9	Yes	**	0.0013	B-C		
Test details	Mean 1	Mean 2	Mean Diff.	SE of diff.	n1	n2	t	DF
CT vs. OBS	255.7	418.0	-162.3	48.96	5	6	3.315	14
CT vs. OBexT	255.7	204.0	51.75	48.96	5	6	1.057	14
OBS vs. OBexT	418.0	204.0	214.0	46.68	6	6	4.585	14

Fig. 2B

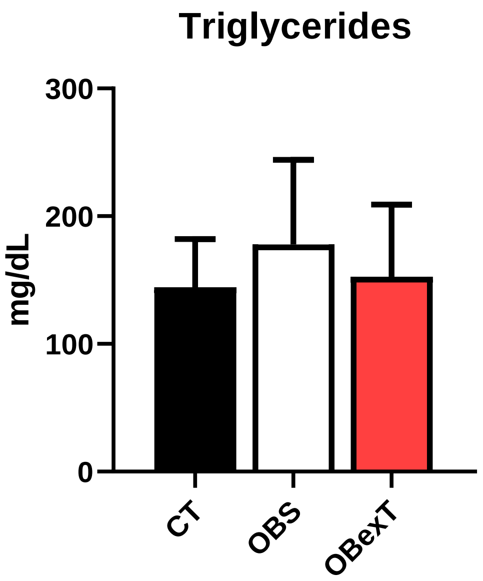

CT	OBS	OBexT
121.212121	115.1515	136.3636
125.757576	124.2424	107.5758
130.303030	115.1515	116.6667
133.333333	253.0303	110.6061
211.363636	218.1818	200.7576
	240.9091	243.9394

Bonferroni's multiple comparisons test	Mean Diff.	95.00% CI of diff.	Significant?	Summary	Adjusted P Value			
CT vs. OBS	-33.38	-125.3 to 58.55	No	ns	>0.9999	E-F		
CT vs. OBexT	-8.258	-100.2 to 83.67	No	ns	>0.9999	E-G		
OBS vs. OBexT	25.13	-62.53 to 112.8	No	ns	>0.9999	F-G		
Test details	Mean 1	Mean 2	Mean Diff.	SE of diff.	n1	n2	t	DF
CT vs. OBS	144.4	177.8	-33.38	33.83	5	6	0.9869	14
CT vs. OBexT	144.4	152.7	-8.258	33.83	5	6	0.2441	14
OBS vs. OBexT	177.8	152.7	25.13	32.25	6	6	0.7790	14

Fig. 2C

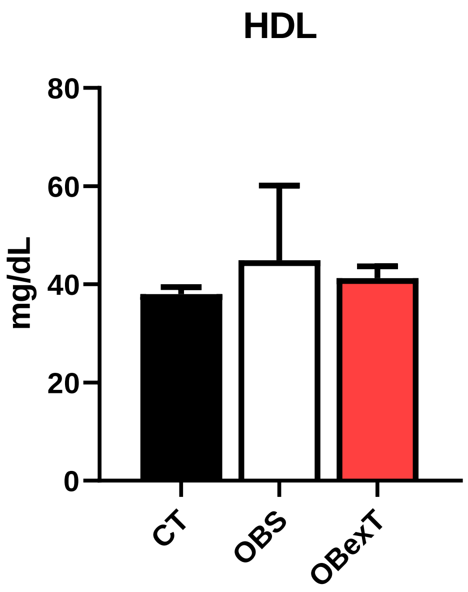

CT	OBS	OBexT
36.889	37.333	40.000
37.778	40.444	40.889
36.889	37.778	41.778
40.444	37.778	45.778
38.000	40.444	40.444
	75.778	38.889

Bonferroni's multiple comparisons test	Mean Diff.	95.00% CI of diff.	Significant?	Summary	Adjusted P Value			
CT vs. OBS	-6.926	-22.09 to 8.240	No	ns	0.7049	M-N		
CT vs. OBexT	-3.296	-18.46 to 11.87	No	ns	>0.9999	M-O		
OBS vs. OBexT	3.630	-10.83 to 18.09	No	ns	>0.9999	N-O		
**Test details**	**Mean 1**	**Mean 2**	**Mean Diff.**	**SE of diff.**	**n1**	**n2**	**t**	**DF**
CT vs. OBS	38.00	44.93	-6.926	5.580	5	6	1.241	14
CT vs. OBexT	38.00	41.30	-3.296	5.580	5	6	0.5907	14
OBS vs. OBexT	44.93	41.30	3.630	5.321	6	6	0.6821	14

Fig. 3A

Saturated Fatty Acids

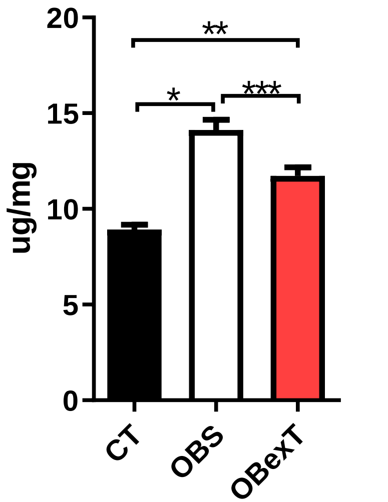

CT	OBS	OBexT
8.56	12.26	11.11
8.61	14.63	12.06
8.42	15.99	10.34
9.21	14.24	12.00
9.80	14.63	13.08
	12.96	9.63

Bonferroni's multiple comparisons test	Mean Diff.	95.00% CI of diff.	Significant?	Summary				
CT vs. OBS	-5.198	-7.071 to -3.326	Yes	****		A-B		
CT vs. OBexT	-2.450	-4.322 to -0.5776	Yes	**		A-C		
OBS vs. OBexT	2.748	0.9630 to 4.534	Yes	**		B-C		
**Test details**	**Mean 1**	**Mean 2**	**Mean Diff.**	**SE of diff.**	**n1**	**n2**	**t**	**DF**
CT vs. OBS	8.920	14.12	-5.198	0.6890	5	6	7.545	14
CT vs. OBexT	8.920	11.37	-2.450	0.6890	5	6	3.556	14
OBS vs. OBexT	14.12	11.37	2.748	0.6569	6	6	4.184	14

Fig. 3B

Monounsaturated Fatty Acids

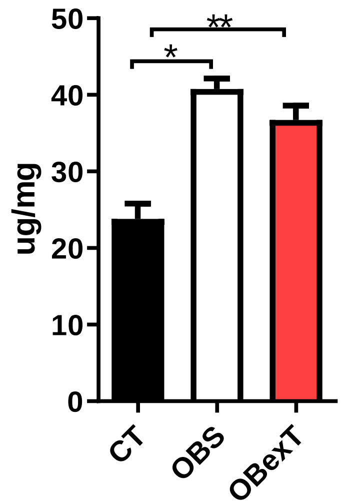

CT	OBS	OBexT
17.81	35.26	35.86
24.62	44.15	40.53
21.06	42.72	38.97
21.82	41.17	34.26
25.49	43.06	41.55
	38.23	29.32

Bonferroni's multiple comparisons test	Mean Diff.	95.00% CI of diff.	Significant?	Summary				
CT vs. OBS	-18.61	-24.81 to -12.40	Yes	****		A-B		
CT vs. OBexT	-14.59	-20.80 to -8.379	Yes	****		A-C		
OBS vs. OBexT	4.017	-1.903 to 9.937	No	ns		B-C		
**Test details**	**Mean 1**	**Mean 2**	**Mean Diff.**	**SE of diff.**	**n1**	**n2**	**t**	**DF**
CT vs. OBS	22.16	40.77	-18.61	2.285	5	6	8.144	14
CT vs. OBexT	22.16	36.75	-14.59	2.285	5	6	6.386	14
OBS vs. OBexT	40.77	36.75	4.017	2.178	6	6	1.844	14

Fig. 3C

Polyunsaturated Fatty Acids

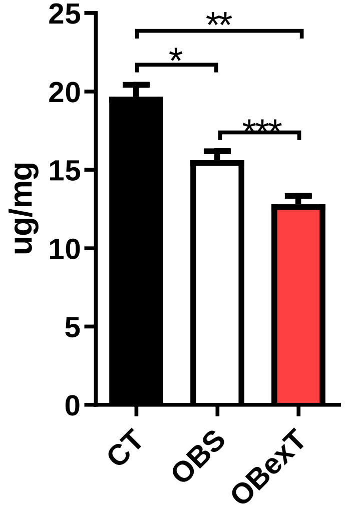

CT	OBS	OBexT
17.79	13.39	12.06
21.78	17.54	13.88
18.66	15.37	13.03
18.93	15.55	11.63
21.17	16.64	14.65
	15.23	11.61

Bonferroni's multiple comparisons test	Mean Diff.	95.00% CI of diff.	Significant?	Summary				
CT vs. OBS	4.046	1.649 to 6.443	Yes	**		A-B		
CT vs. OBexT	6.856	4.459 to 9.253	Yes	****		A-C		
OBS vs. OBexT	2.810	0.5247 to 5.095	Yes	*		B-C		
**Test details**	**Mean 1**	**Mean 2**	**Mean Diff.**	**SE of diff.**	**n1**	**n2**	**t**	**DF**
CT vs. OBS	19.67	15.62	4.046	0.8819	5	6	4.588	14
CT vs. OBexT	19.67	12.81	6.856	0.8819	5	6	7.774	14
OBS vs. OBexT	15.62	12.81	2.810	0.8409	6	6	3.342	14

Fig. 3D

C14:0

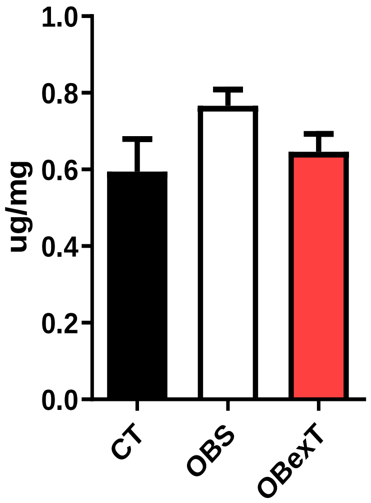

CT	OBS	OBexT
0.96	0.64	0.47
0.44	0.81	0.66
0.47	0.85	0.59
0.69	0.65	0.79
0.60	0.89	0.74
	0.76	0.63

Bonferroni's multiple comparisons test	Mean Diff.	95.00% CI of diff.	Significant?	Summary				
CT vs. OBS	-0.1347	-0.3727 to 0.1034	No	ns		A-B		
CT vs. OBexT	-0.01467	-0.2527 to 0.2234	No	ns		A-C		
OBS vs. OBexT	0.1200	-0.1070 to 0.3470	No	ns		B-C		
**Test details**	**Mean 1**	**Mean 2**	**Mean Diff.**	**SE of diff.**	**n1**	**n2**	**t**	**DF**
CT vs. OBS	0.6320	0.7667	-0.1347	0.08759	5	6	1.537	14
CT vs. OBexT	0.6320	0.6467	-0.01467	0.08759	5	6	0.1674	14
OBS vs. OBexT	0.7667	0.6467	0.1200	0.08352	6	6	1.437	14

Fig. 3E

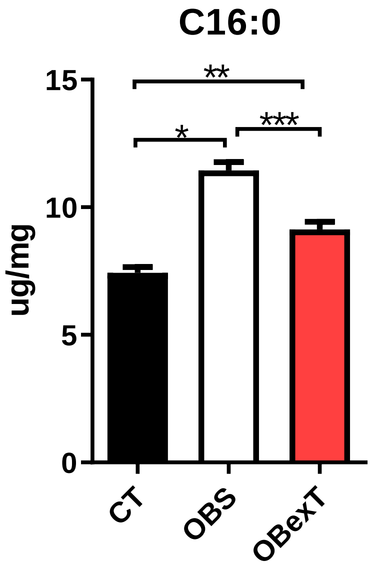

CT	OBS	OBexT
6.90	9.62	8.75
7.31	11.71	9.28
7.16	12.37	8.41
7.59	11.35	9.03
8.22	11.41	10.17
	10.39	7.59

Bonferroni's multiple comparisons test	Mean Diff.	95.00% CI of diff.	Significant?	Summary				
CT vs. OBS	-3.706	-5.068 to -2.343	Yes	****		A-B		
CT vs. OBexT	-1.436	-2.798 to -0.07337	Yes	*		A-C		
OBS vs. OBexT	2.270	0.9711 to 3.569	Yes	***		B-C		
**Test details**	**Mean 1**	**Mean 2**	**Mean Diff.**	**SE of diff.**	**n1**	**n2**	**t**	**DF**
CT vs. OBS	7.436	11.14	-3.706	0.5013	5	6	7.393	14
CT vs. OBexT	7.436	8.872	-1.436	0.5013	5	6	2.864	14
OBS vs. OBexT	11.14	8.872	2.270	0.4779	6	6	4.750	14

Fig. 3F

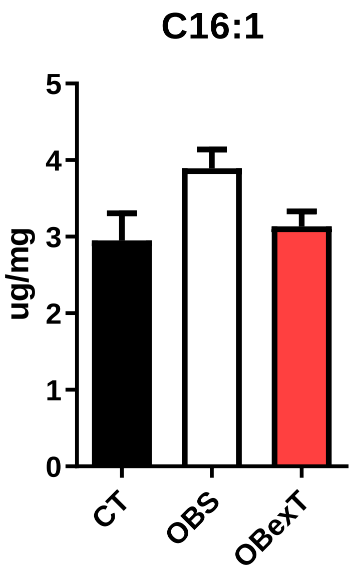

CT	OBS	OBexT
3.97	2.89	2.67
1.49	4.75	3.08
3.31	4.03	3.99
2.62	3.77	2.82
3.50	3.90	3.39
	4.02	2.85

Bonferroni's multiple comparisons test	Mean Diff.	95.00% CI of diff.	Significant?	Summary				
CT vs. OBS	-0.9153	-2.053 to 0.2227	No	ns		A-B		
CT vs. OBexT	-0.1553	-1.293 to 0.9827	No	ns		A-C		
OBS vs. OBexT	0.7600	-0.3251 to 1.845	No	ns		B-C		
**Test details**	**Mean 1**	**Mean 2**	**Mean Diff.**	**SE of diff.**	**n1**	**n2**	**t**	**DF**
CT vs. OBS	2.978	3.893	-0.9153	0.4188	5	6	2.186	14
CT vs. OBexT	2.978	3.133	-0.1553	0.4188	5	6	0.3709	14
OBS vs. OBexT	3.893	3.133	0.7600	0.3993	6	6	1.903	14

Fig. 3G

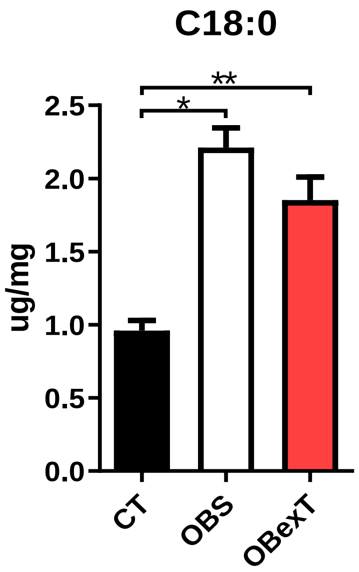

CT	OBS	OBexT
1.21	2.01	1.89
0.83	2.10	2.12
0.84	2.77	1.35
0.94	2.24	2.18
0.98	2.33	2.17
	1.82	1.41

Bonferroni's multiple comparisons test	Mean Diff.	95.00% CI of diff.	Significant?	Summary				
CT vs. OBS	-1.252	-1.764 to -0.7393	Yes	****		A-B		
CT vs. OBexT	-0.8933	-1.406 to -0.3810	Yes	***		A-C		
OBS vs. OBexT	0.3583	-0.1302 to 0.8468	No	ns		B-C		
**Test details**	**Mean 1**	**Mean 2**	**Mean Diff.**	**SE of diff.**	**n1**	**n2**	**t**	**DF**
CT vs. OBS	0.9600	2.212	-1.252	0.1885	5	6	6.639	14
CT vs. OBexT	0.9600	1.853	-0.8933	0.1885	5	6	4.739	14
OBS vs. OBexT	2.212	1.853	0.3583	0.1797	6	6	1.994	14

Fig. 3H

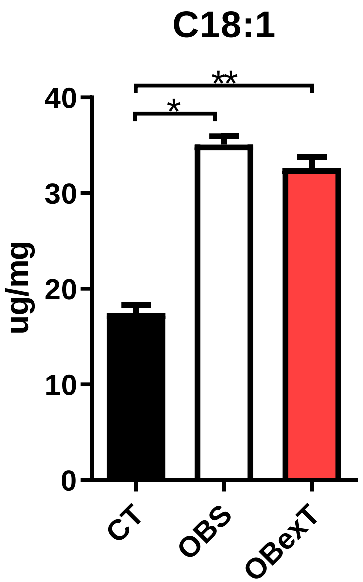

CT	OBS	OBexT
14.79	30.08	30.95
19.06	36.52	34.70
16.62	35.98	32.48
17.07	34.78	29.23
19.66	36.28	35.64
	31.80	24.66

Bonferroni's multiple comparisons test	Mean Diff.	95.00% CI of diff.	Significant?	Summary				
CT vs. OBS	-16.80	-21.85 to -11.75	Yes	****		A-B		
CT vs. OBexT	-13.84	-18.88 to -8.791	Yes	****		A-C		
OBS vs. OBexT	2.963	-1.848 to 7.774	No	ns		B-C		
**Test details**	**Mean 1**	**Mean 2**	**Mean Diff.**	**SE of diff.**	**n1**	**n2**	**t**	**DF**
CT vs. OBS	17.44	34.24	-16.80	1.857	5	6	9.049	14
CT vs. OBexT	17.44	31.28	-13.84	1.857	5	6	7.453	14
OBS vs. OBexT	34.24	31.28	2.963	1.770	6	6	1.674	14

Fig. 3I

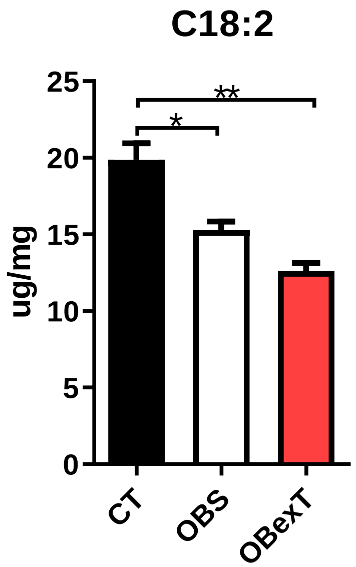

CT	OBS	OBexT
24.28	13.13	11.90
20.98	17.12	13.73
18.03	15.02	12.83
18.20	15.24	11.40
20.52	16.30	14.45
	14.87	11.36

Bonferroni's multiple comparisons test	Mean Diff.	95.00% CI of diff.	Significant?	Summary		
CT vs. OBS	5.122	2.226 to 8.018	Yes	***		A-B
CT vs. OBexT	7.790	4.894 to 10.69	Yes	****		A-C
OBS vs. OBexT	2.668	-0.09329 to 5.430	No	ns		B-C

Test details	Mean 1	Mean 2	Mean Diff.	SE of diff.	n1	n2	t	DF
CT vs. OBS	20.40	15.28	5.122	1.066	5	6	4.806	14
CT vs. OBexT	20.40	12.61	7.790	1.066	5	6	7.310	14
OBS vs. OBexT	15.28	12.61	2.668	1.016	6	6	2.626	14

Fig. 3J

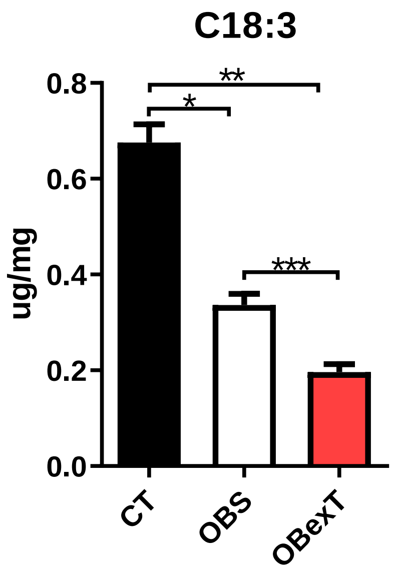

CT	OBS	OBexT
0.58	0.25	0.15
0.79	0.42	0.15
0.63	0.35	0.20
0.73	0.31	0.23
0.65	0.34	0.20
	0.35	0.25

Bonferroni's multiple comparisons test	Mean Diff.	95.00% CI of diff.	Significant?	Summary		
CT vs. OBS	0.3393	0.2392 to 0.4394	Yes	****		A-B
CT vs. OBexT	0.4793	0.3792 to 0.5794	Yes	****		A-C
OBS vs. OBexT	0.1400	0.04457 to 0.2354	Yes	**		B-C

Test details	Mean 1	Mean 2	Mean Diff.	SE of diff.	n1	n2	t	DF
CT vs. OBS	0.6760	0.3367	0.3393	0.03683	5	6	9.214	14
CT vs. OBexT	0.6760	0.1967	0.4793	0.03683	5	6	13.02	14
OBS vs. OBexT	0.3367	0.1967	0.1400	0.03511	6	6	3.987	14

Fig. 3K

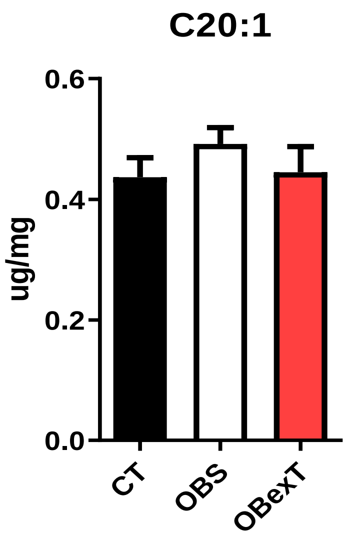

CT	OBS	OBexT
0.48	0.44	0.43
0.40	0.54	0.61
0.46	0.45	0.43
0.35	0.49	0.50
0.37	0.60	0.40
	0.43	0.30

Bonferroni's multiple comparisons test	Mean Diff.	95.00% CI of diff.	Significant?	Summary				
CT vs. OBS	-0.07967	-0.2107 to 0.05133	No	ns		A-B		
CT vs. OBexT	-0.03300	-0.1640 to 0.09800	No	ns		A-C		
OBS vs. OBexT	0.04667	-0.07824 to 0.1716	No	ns		B-C		
**Test details**	**Mean 1**	**Mean 2**	**Mean Diff.**	**SE of diff.**	**n1**	**n2**	**t**	**DF**
CT vs. OBS	0.4120	0.4917	-0.07967	0.04820	5	6	1.653	14
CT vs. OBexT	0.4120	0.4450	-0.03300	0.04820	5	6	0.6846	14
OBS vs. OBexT	0.4917	0.4450	0.04667	0.04596	6	6	1.015	14

Fig. 4A and B

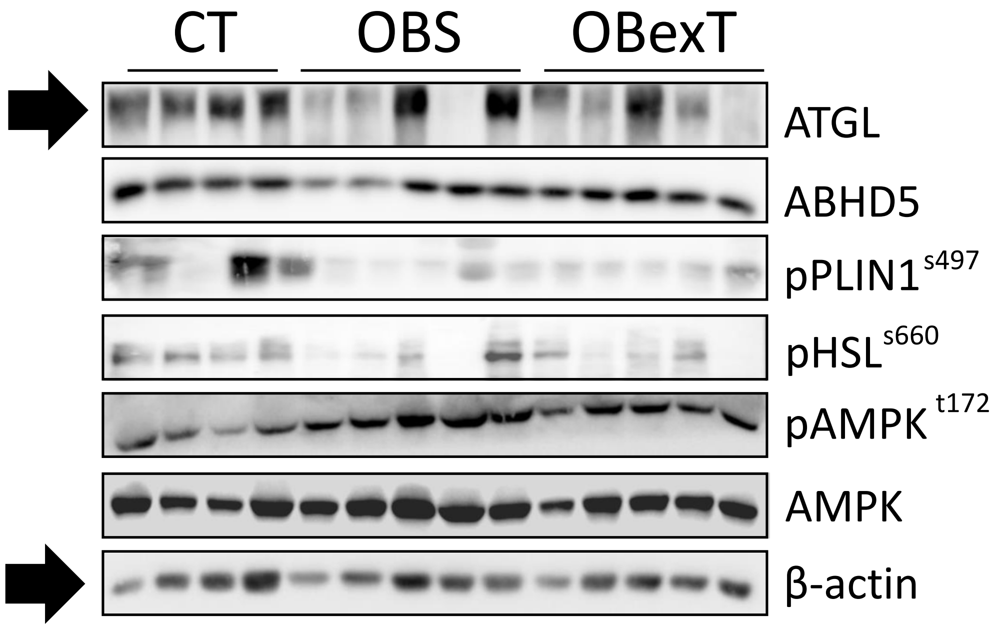

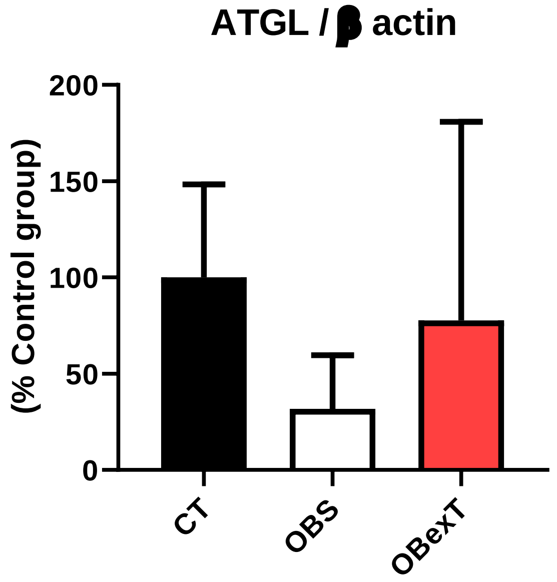

CT	OBS	OBexT
168.657	76.818	49.914
96.966	26.265	10.153
59.015	0.011	254.391
75.362	29.220	73.864
	26.695	0.000

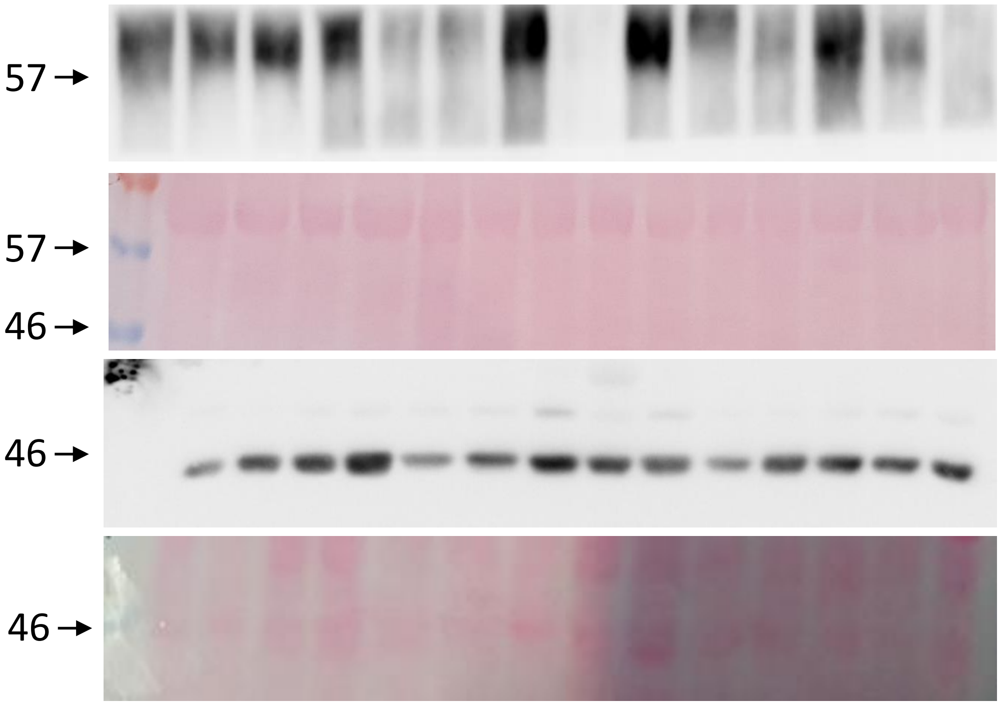

ATGL

Original ATGL

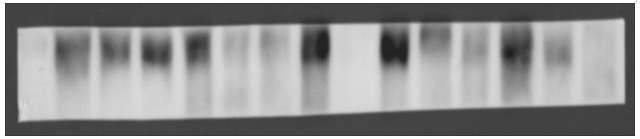

β-ACTIN

Original β-ACTIN

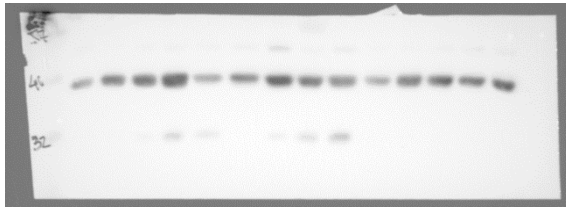

Tukey's multiple comparisons test	Mean Diff.	95.00% CI of diff.	Significant?	Summary	Adjusted P Value			
CT vs. OBS	68.20	-57.23 to 193.6	No	ns	0.3422	A-B		
CT vs. OBexT	22.34	-103.1 to 147.8	No	ns	0.8816	A-C		
OBS vs. OBexT	-45.86	-164.1 to 72.39	No	ns	0.5641	B-C		
Test details	Mean 1	Mean 2	Mean Diff.	SE of diff.	n1	n2	q	DF
CT vs. OBS	100.0	31.80	68.20	46.44	4	5	2.077	11
CT vs. OBexT	100.0	77.66	22.34	46.44	4	5	0.6802	11
OBS vs. OBexT	31.80	77.66	-45.86	43.78	5	5	1.481	11

Fig. 4A and C

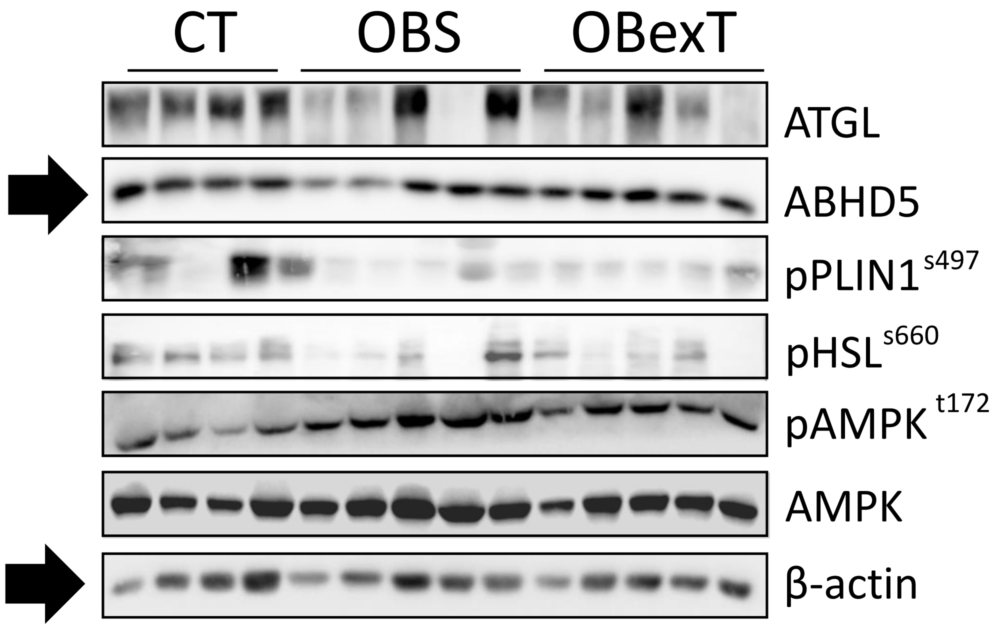

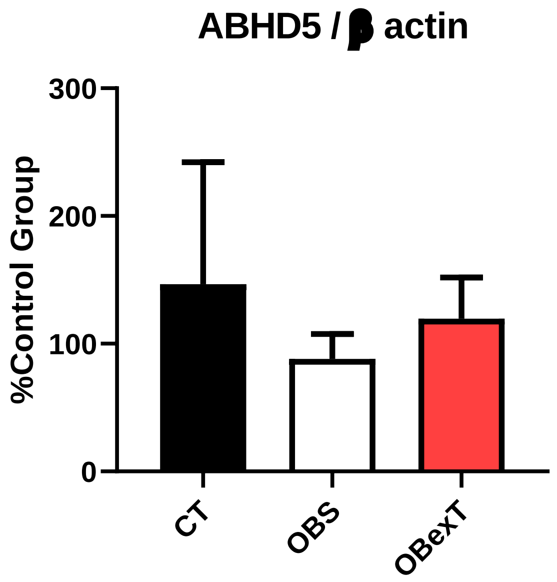

CT	OBS	OBexT
286.414	97.921	175.489
127.103	63.484	107.052
97.016	71.115	113.194
75.881	100.662	110.837
	107.156	91.971

Tukey's multiple comparisons test	Mean Diff.	95.00% CI of diff.	Significant?	Summary	Adjusted P Value			
CT vs. OBS	58.54	-40.79 to 157.9	No	ns	0.2896	AC-AD		
CT vs. OBexT	26.89	-72.43 to 126.2	No	ns	0.7506	AC-AE		
OBS vs. OBexT	-31.64	-125.3 to 62.01	No	ns	0.6439	AD-AE		
Test details	Mean 1	Mean 2	Mean Diff.	SE of diff.	n1	n2	q	DF
CT vs. OBS	146.6	88.07	58.54	36.78	4	5	2.251	11
CT vs. OBexT	146.6	119.7	26.89	36.78	4	5	1.034	11
OBS vs. OBexT	88.07	119.7	-31.64	34.67	5	5	1.291	11

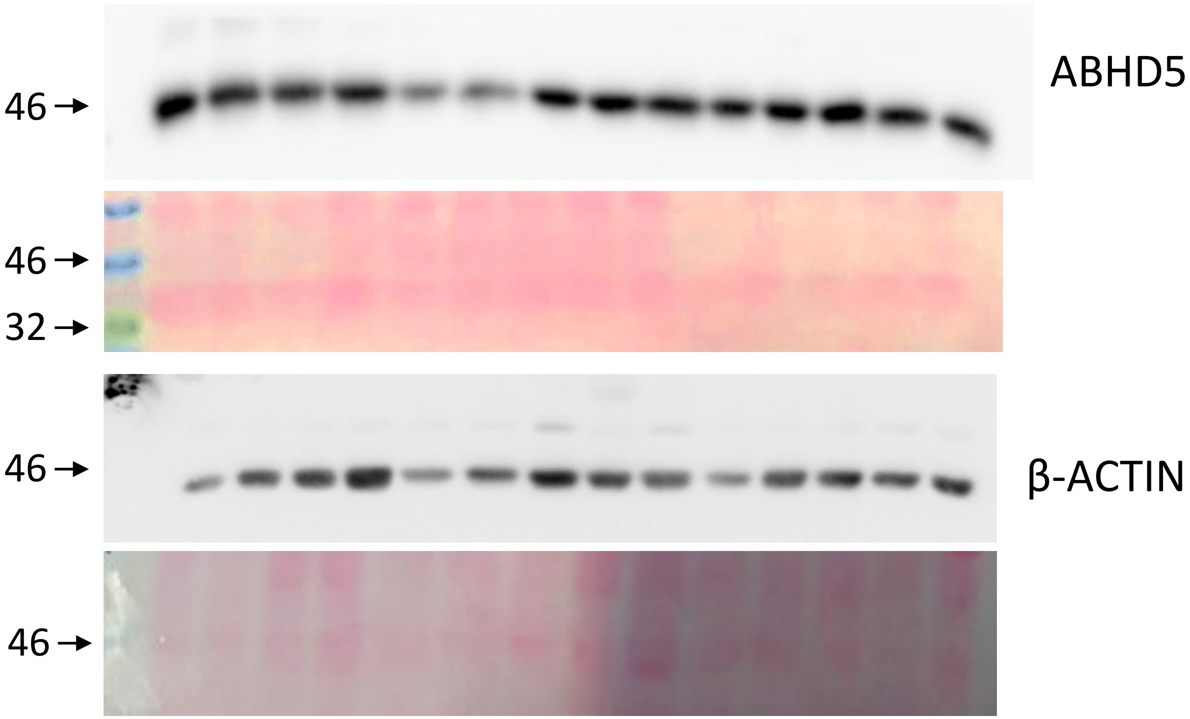

Original ABHD5

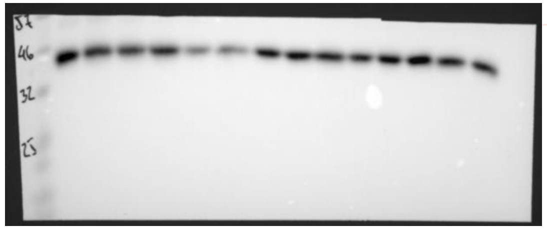

Fig. 4A and D

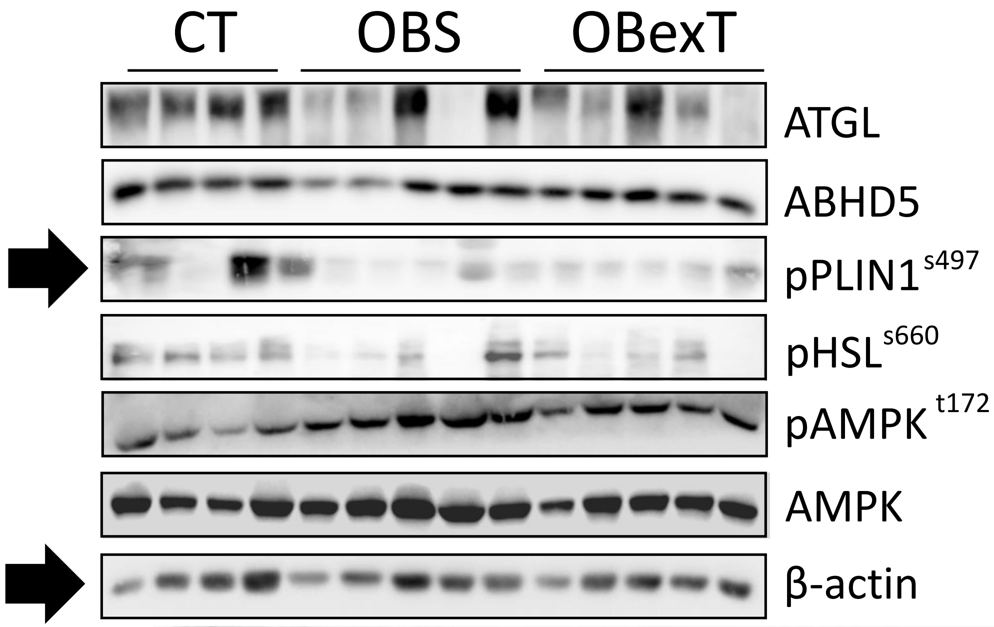

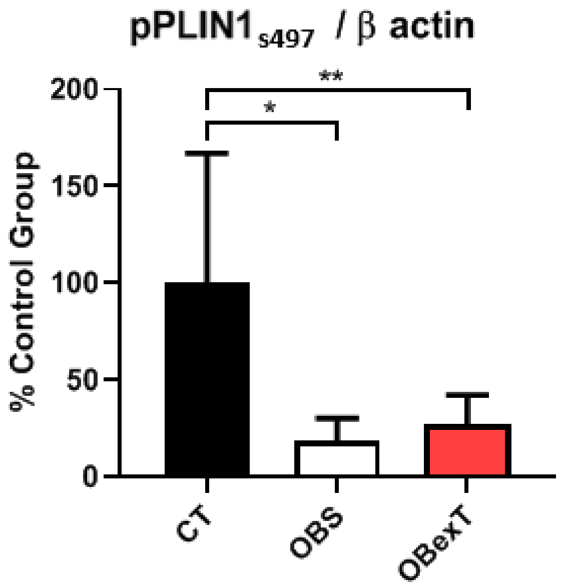

CT	OBS	OBexT
154.017	23.320	36.292
27.239	8.311	15.089
159.723	7.577	12.076
59.021	35.277	24.623
	19.047	48.496

Tukey's multiple comparisons test	Mean Diff.	95.00% CI of diff.	Significant?	Summary	Adjusted P Value			
CT vs. OBS	81.29	14.60 to 148.0	Yes	*	0.0181	AG-AH		
CT vs. OBexT	72.68	5.994 to 139.4	Yes	*	0.0330	AG-AI		
OBS vs. OBexT	-8.609	-71.49 to 54.27	No	ns	0.9279	AH-AI		
Test details	Mean 1	Mean 2	Mean Diff.	SE of diff.	n1	n2	q	DF
CT vs. OBS	100.0	18.71	81.29	24.69	4	5	4.656	11
CT vs. OBexT	100.0	27.32	72.68	24.69	4	5	4.163	11
OBS vs. OBexT	18.71	27.32	-8.609	23.28	5	5	0.5230	11

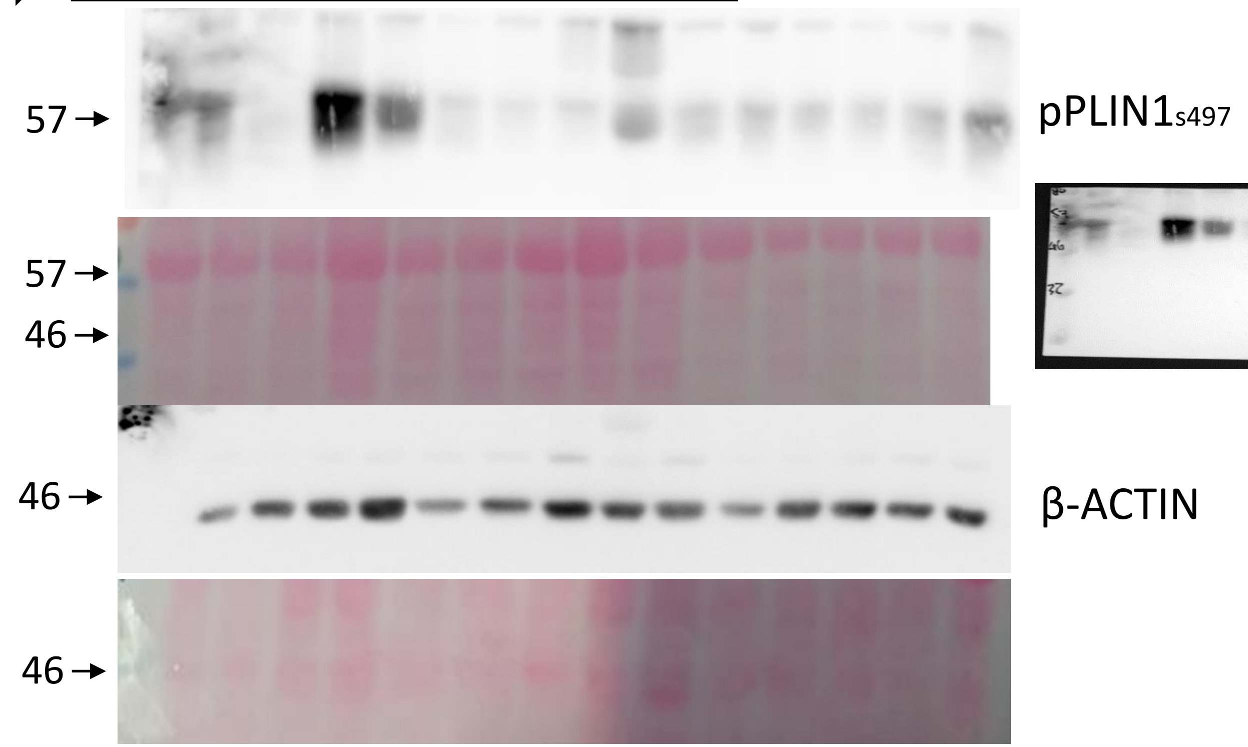

Original pPLIN1s497

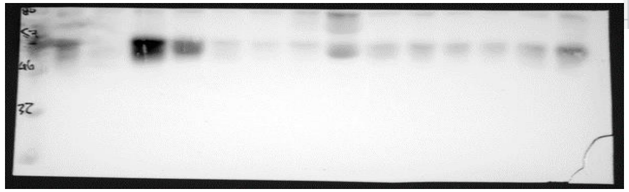

Fig. 4A and E

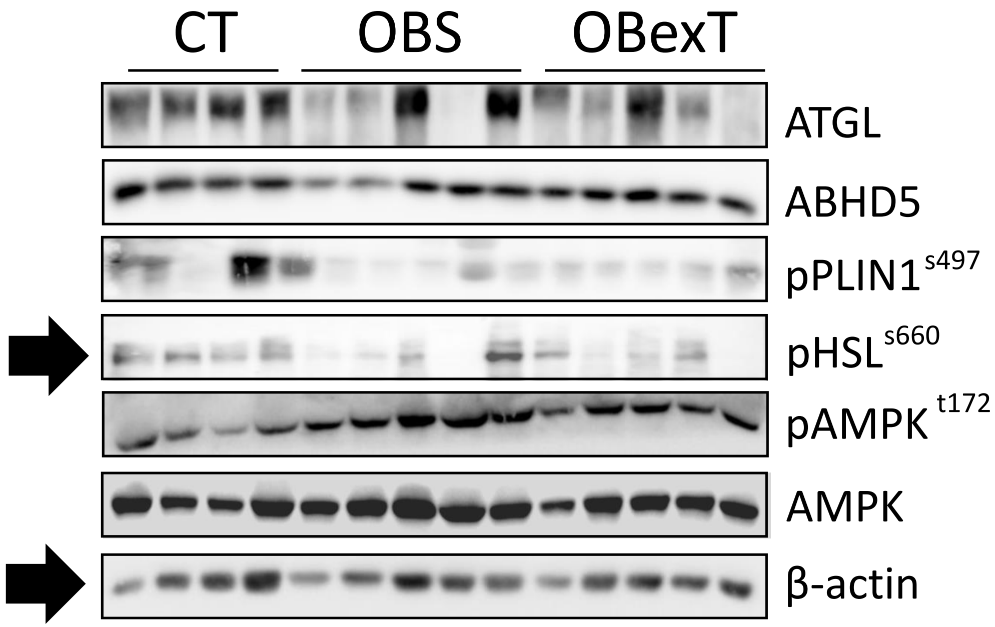

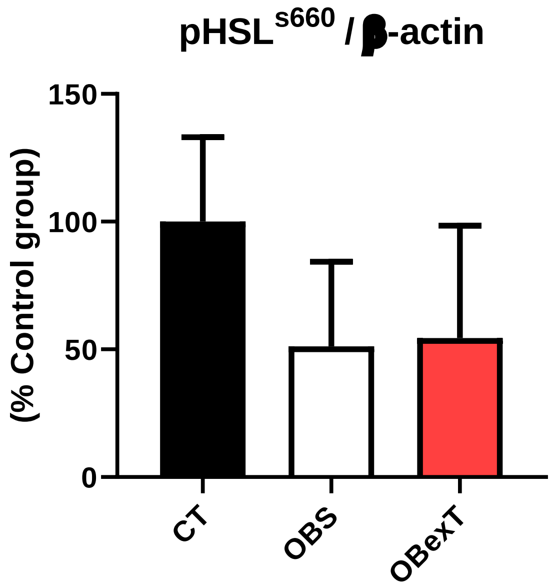

CT	OBS	OBexT
120.165	75.730	29.087
135.266	35.280	43.094
79.378	30.060	129.401
65.191	96.175	52.923
	18.911	17.758

Tukey's multiple comparisons test	Mean Diff.	95.00% CI of diff.	Significant?	Summary	Adjusted P Value			
CT vs. OBS	48.77	-19.01 to 116.5	No	ns	0.1727	A-B		
CT vs. OBexT	45.55	-22.23 to 113.3	No	ns	0.2099	A-C		
OBS vs. OBexT	-3.221	-67.12 to 60.68	No	ns	0.9898	B-C		
**Test details**	**Mean 1**	**Mean 2**	**Mean Diff.**	**SE of diff.**	**n1**	**n2**	**q**	**DF**
CT vs. OBS	100.0	51.23	48.77	25.09	4	5	2.748	11
CT vs. OBexT	100.0	54.45	45.55	25.09	4	5	2.567	11
OBS vs. OBexT	51.23	54.45	-3.221	23.66	5	5	0.1926	11

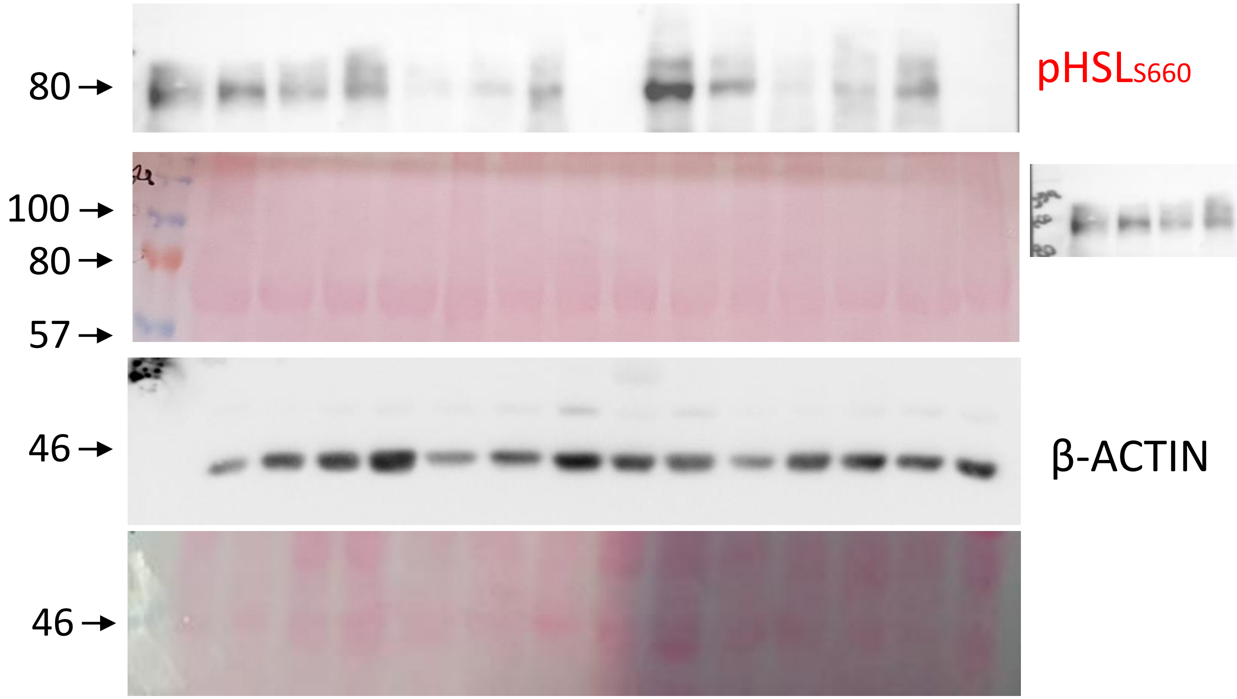

Original pHSLs660

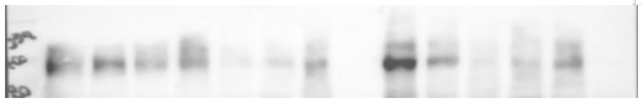

Fig. 4A and F

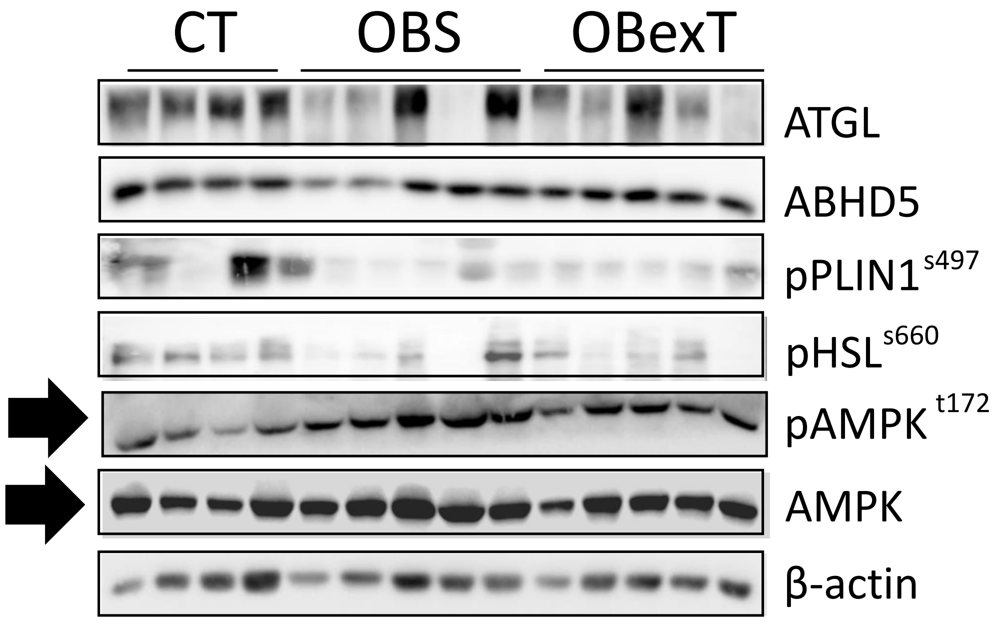

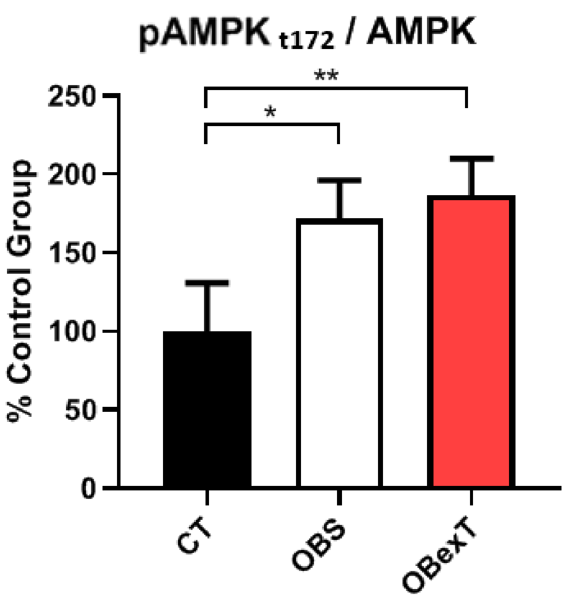

CT	OBS	OBexT
117.204	167.272	213.282
125.924	141.702	195.062
56.939	162.231	200.113
99.933	178.849	158.322
	208.327	167.074

Tukey's multiple comparisons test	Mean Diff.	95.00% CI of diff.	Significant?	Summary	Adjusted P Value			
CT vs. OBS	-54.86	-89.86 to -19.86	Yes	**	0.0037	AK-AL		
CT vs. OBexT	-68.48	-103.5 to -33.47	Yes	***	0.0007	AK-AM		
OBS vs. OBexT	-13.62	-46.62 to 19.38	No	ns	0.5254	AL-AM		
Test details	Mean 1	Mean 2	Mean Diff.	SE of diff.	n1	n2	q	DF
CT vs. OBS	100.0	154.9	-54.86	12.96	4	5	5.987	11
CT vs. OBexT	100.0	168.5	-68.48	12.96	4	5	7.473	11
OBS vs. OBexT	154.9	168.5	-13.62	12.22	5	5	1.576	11

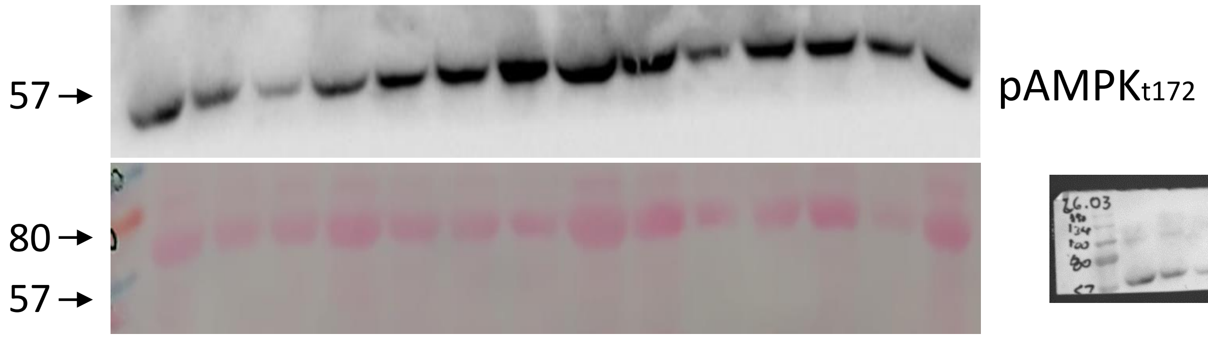

Original pAMPKt172

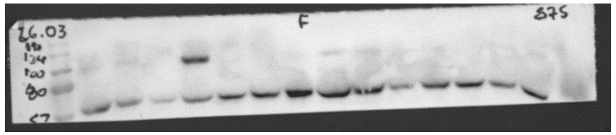

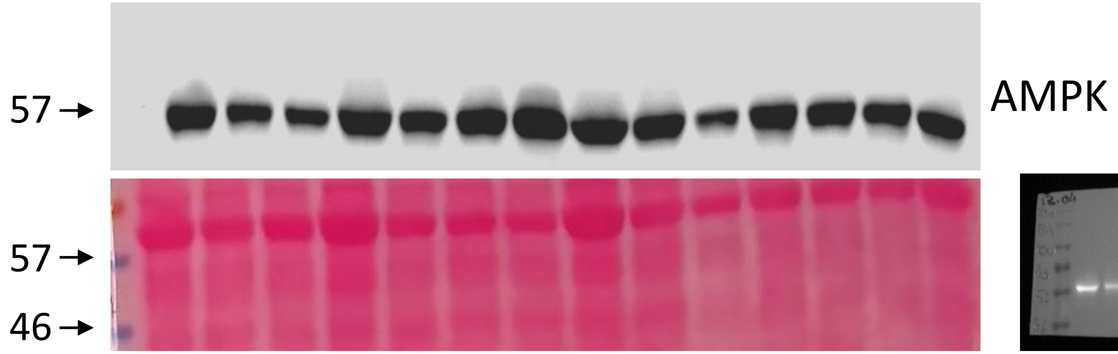

Original AMPK

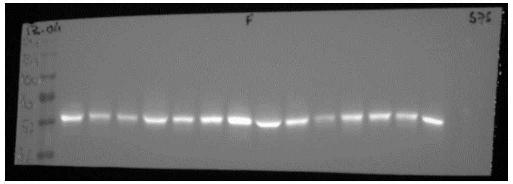

Fig. 5A and B

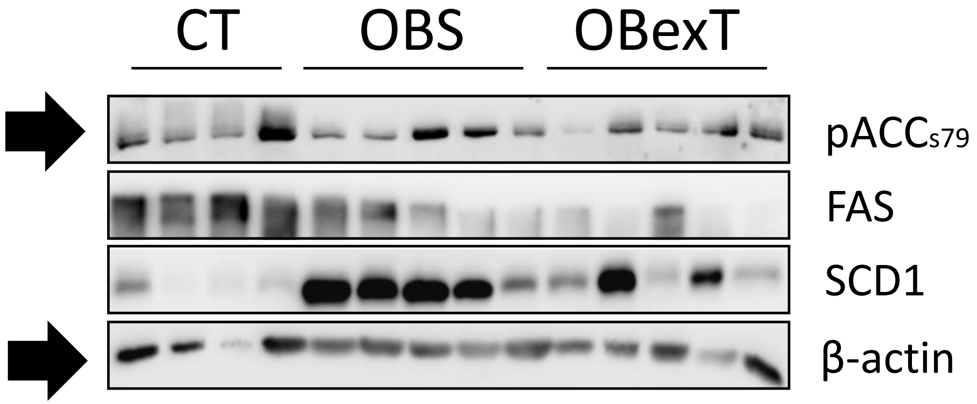

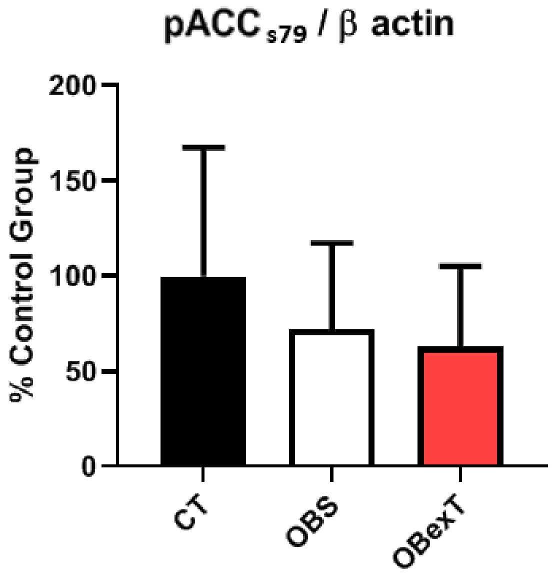

CT	OBS	OBexT
51.686	43.432	15.943
51.221	32.865	88.325
194.377	119.242	31.602
102.715	123.419	120.740
	41.439	56.501

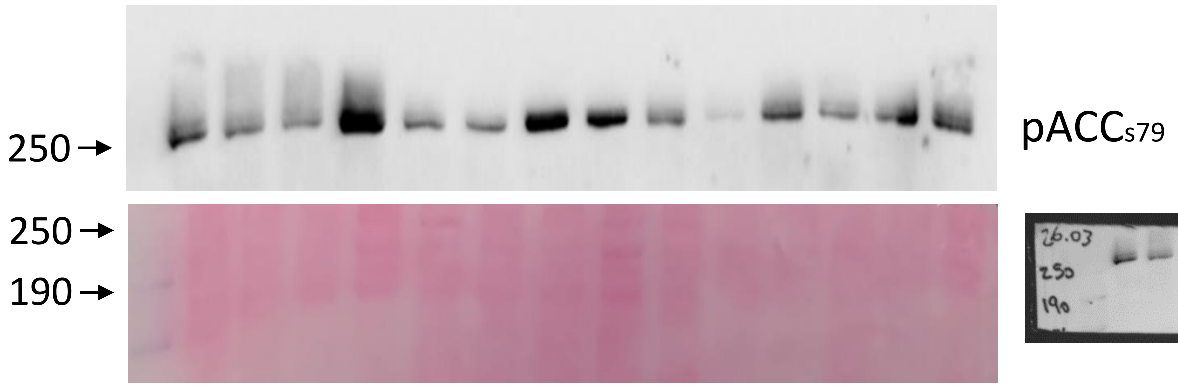

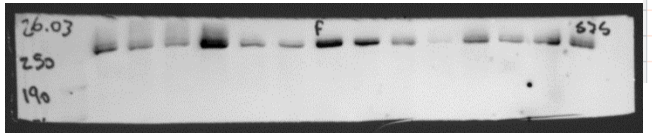

Tukey's multiple comparisons test	Mean Diff.	95.00% CI of diff.	Significant?	Summary	Adjusted P Value			
CT vs. OBS	27.92	-65.11 to 121.0	No	ns	0.7045	Q-R		
CT vs. OBexT	37.38	-55.65 to 130.4	No	ns	0.5422	Q-S		
OBS vs. OBexT	9.457	-78.25 to 97.17	No	ns	0.9545	R-S		
Test details	Mean 1	Mean 2	Mean Diff.	SE of diff.	n1	n2	q	DF
CT vs. OBS	100.0	72.08	27.92	34.45	4	5	1.146	11
CT vs. OBexT	100.0	62.62	37.38	34.45	4	5	1.535	11
OBS vs. OBexT	72.08	62.62	9.457	32.48	5	5	0.4118	11

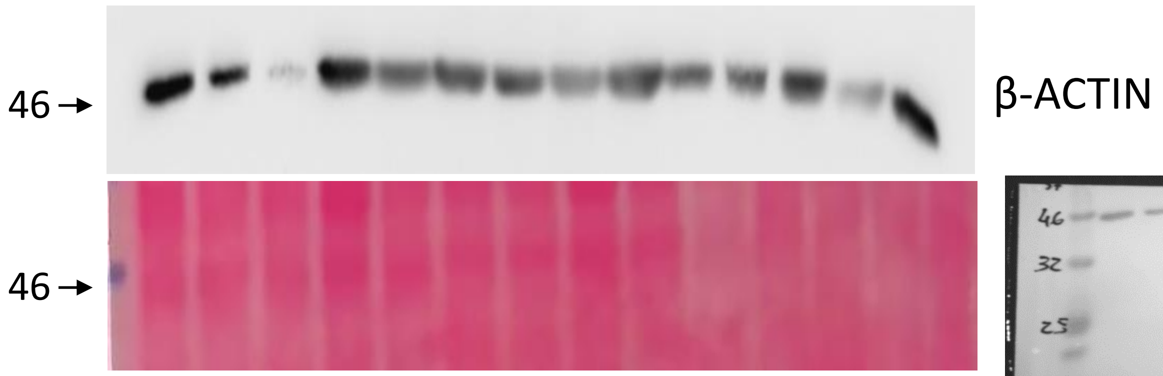

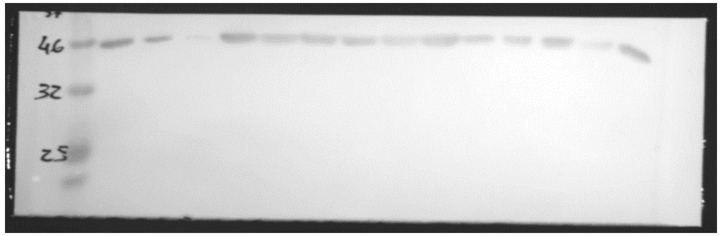

Fig. 5A and C

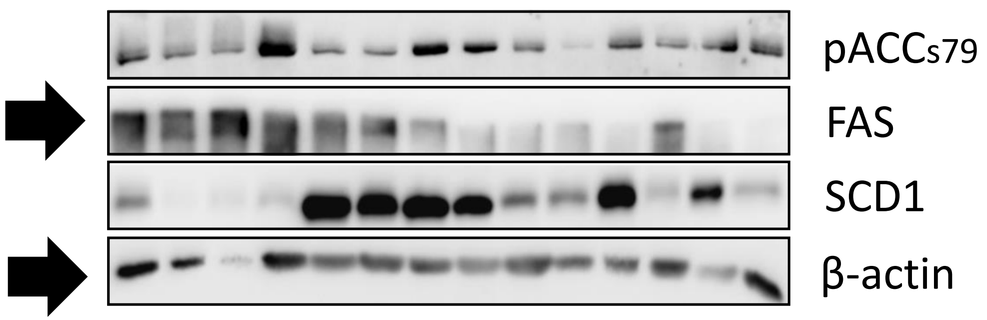

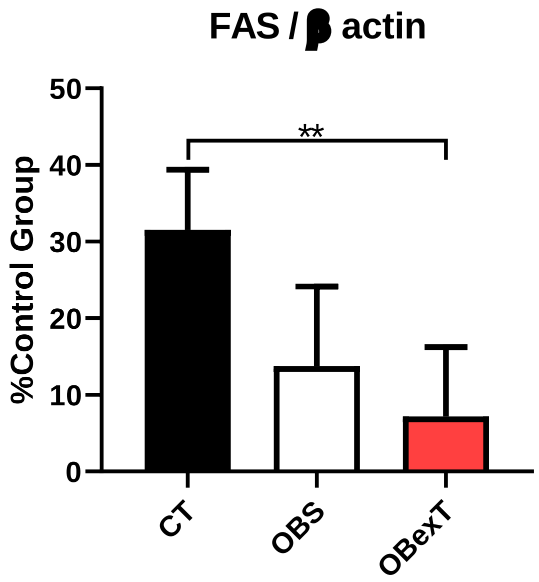

CT	OBS	OBexT
29.089	25.680	8.538
40.314	23.495	3.271
*305.311**	11.580	22.310
25.286	4.486	1.914
	3.513	0.000

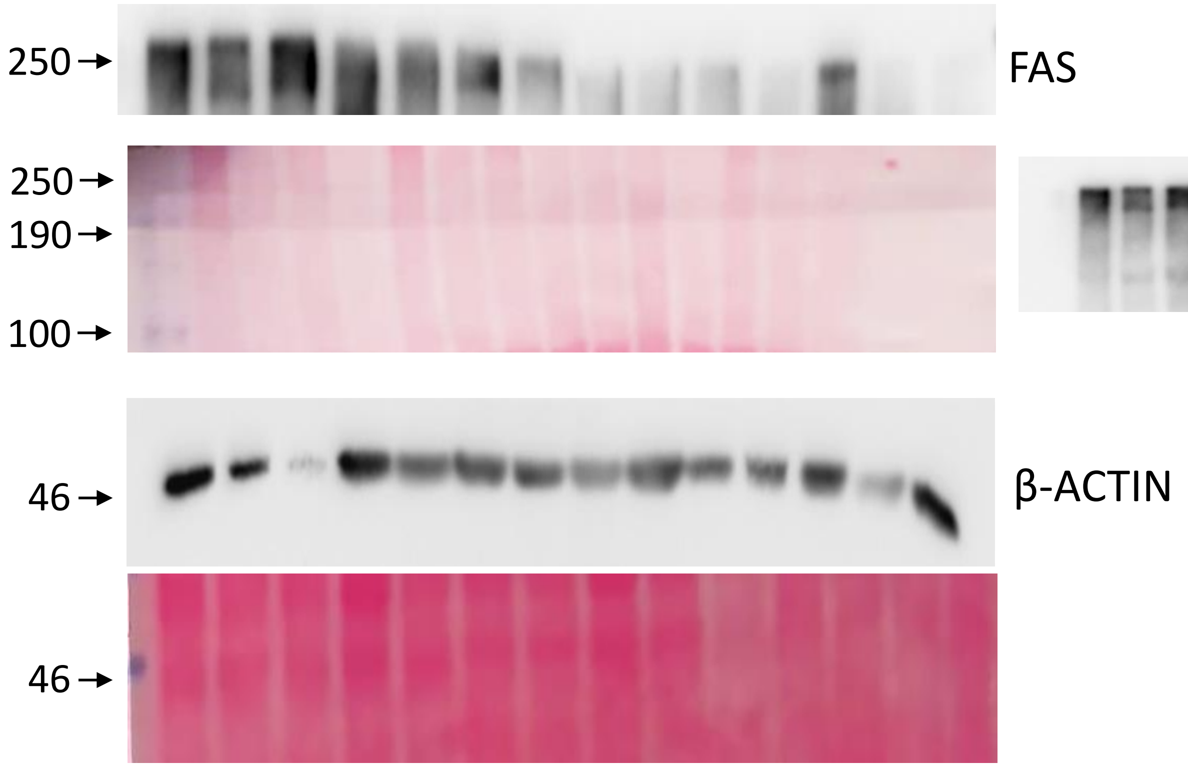

Original FAS

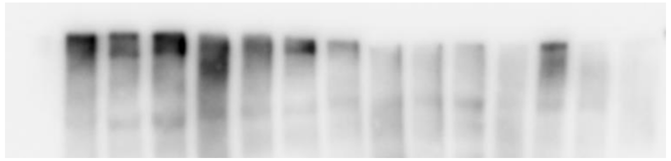

Tukey's multiple comparisons test	Mean Diff.	95.00% CI of diff.	Significant?	Summary	Adjusted P Value			
CT vs. OBS	17.81	-0.9683 to 36.59	No	ns	0.0630	U-V		
CT vs. OBexT	24.36	5.576 to 43.14	Yes	*	0.0132	U-W		
OBS vs. OBexT	6.544	-9.720 to 22.81	No	ns	0.5337	V-W		
Test details	Mean 1	Mean 2	Mean Diff.	SE of diff.	n1	n2	q	DF
CT vs. OBS	31.56	13.75	17.81	6.851	3	5	3.677	10
CT vs. OBexT	31.56	7.207	24.36	6.851	3	5	5.028	10
OBS vs. OBexT	13.75	7.207	6.544	5.933	5	5	1.560	10

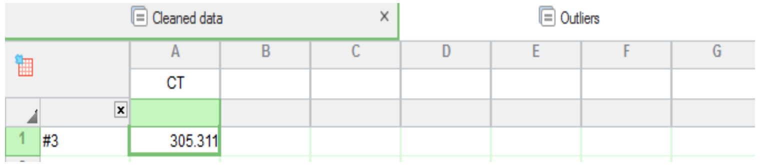

Fig. 5A and D

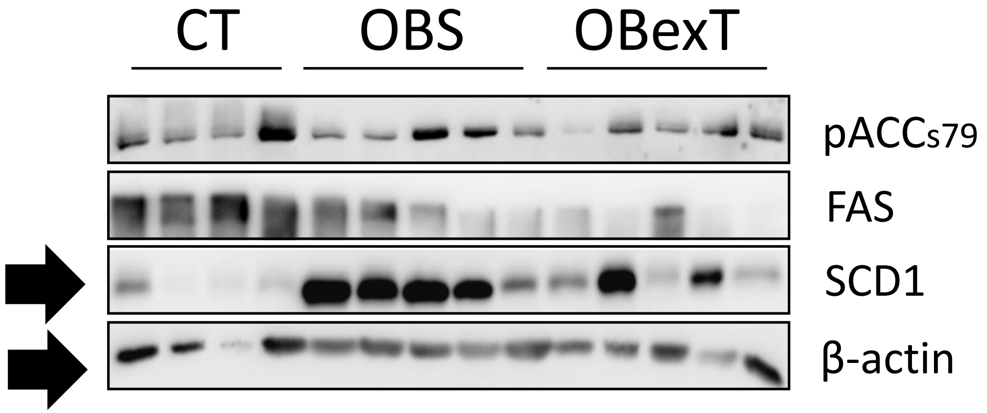

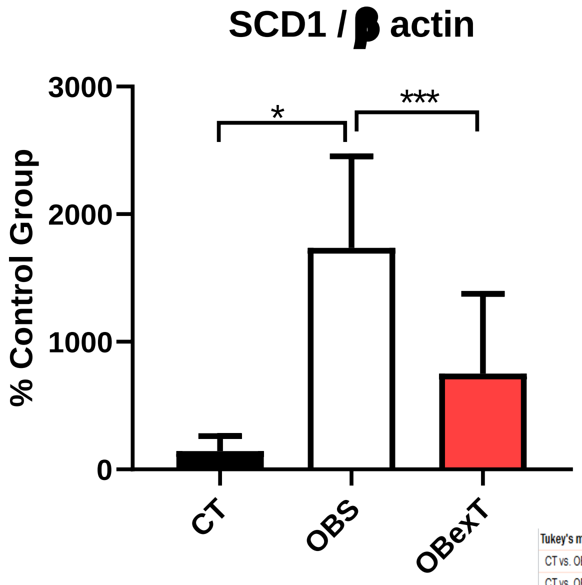

CT	OBS	OBexT
277.568	2310.906	641.678
22.580	1621.000	1251.234
200.444	2051.606	171.162
76.976	2161.602	1532.746
	540.681	162.530

Tukey's multiple comparisons test	Mean Diff.	95.00% CI of diff.	Significant?	Summary	Adjusted P Value			
CT vs. OBS	-1593	-2636 to -549.2	Yes	**	0.0044	Y-Z		
CT vs. OBexT	-607.5	-1651 to 436.1	No	ns	0.2975	Y-AA		
OBS vs. OBexT	985.3	1.408 to 1969	Yes	*	0.0497	Z-AA		
**Test details**	**Mean 1**	**Mean 2**	**Mean Diff.**	**SE of diff.**	**n1**	**n2**	**q**	**DF**
CT vs. OBS	144.4	1737	-1593	386.4	4	5	5.830	11
CT vs. OBexT	144.4	751.9	-607.5	386.4	4	5	2.223	11
OBS vs. OBexT	1737	751.9	985.3	364.3	5	5	3.825	11

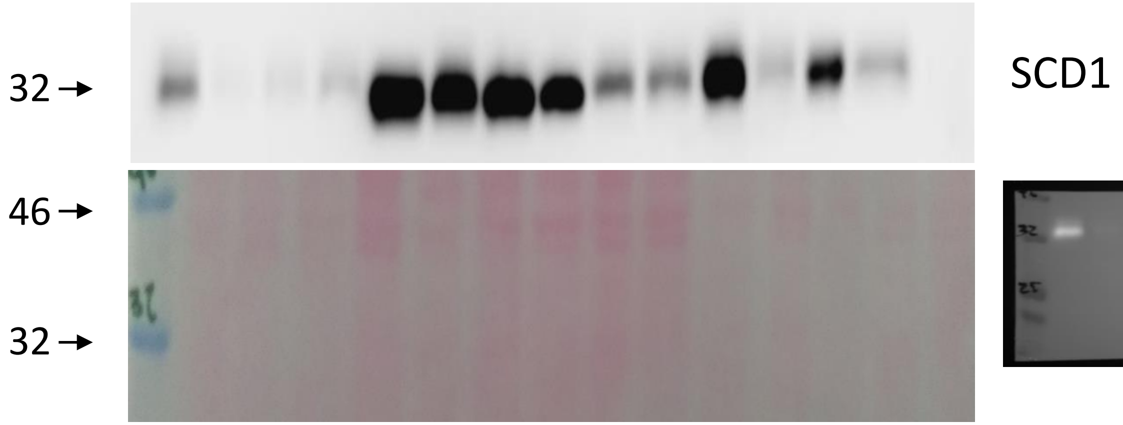

Original SCD1

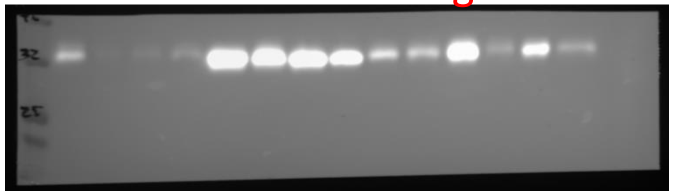

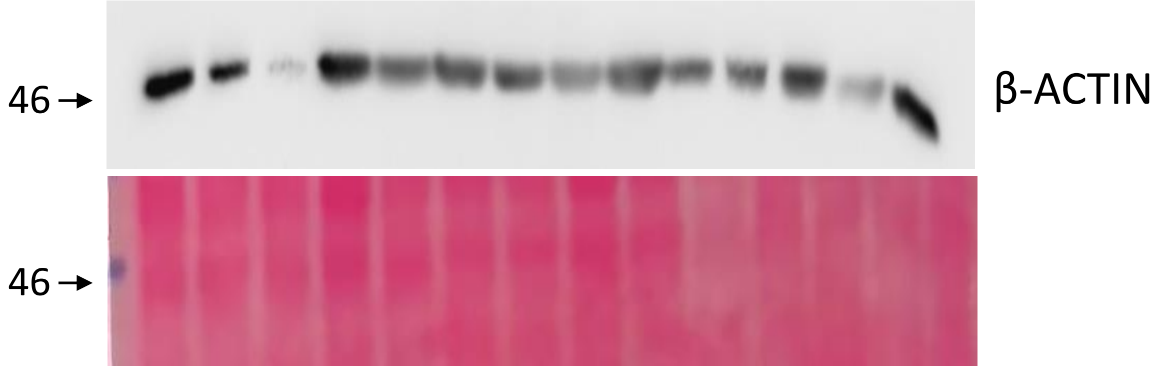

Fig. 6A and B

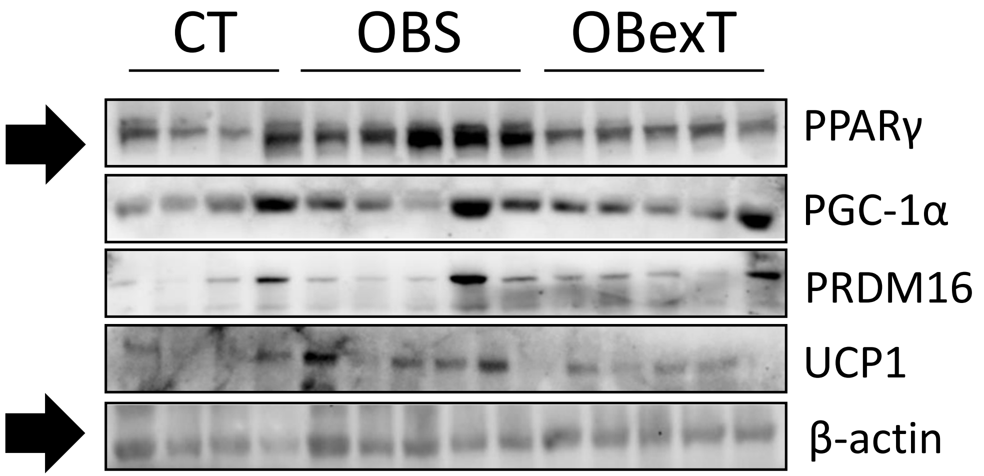

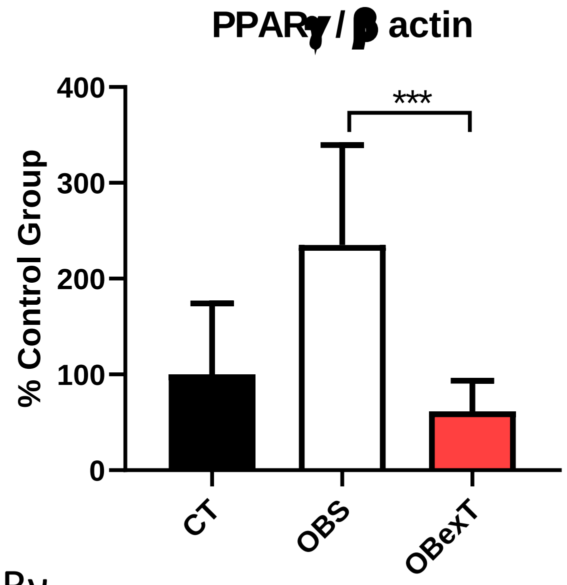

CT	OBS	OBexT
128.91390000	320.5236000	116.05030000
64.093870000	79.47672000	43.291160000
18.799300000	340.0244000	43.124690000
188.19300000	207.4567000	41.123210000
	229.0869000	63.857070000

Tukey's multiple comparisons test	Mean Diff.	95.00% CI of diff.	Significant?	Summary	Adjusted P Value			
CT vs. OBS	-135.3	-273.4 to 2.763	No	ns	0.0548	A-B		
CT vs. OBexT	38.51	-99.57 to 176.6	No	ns	0.7379	A-C		
OBS vs. OBexT	173.8	43.64 to 304.0	Yes	*	0.0106	B-C		
**Test details**	**Mean 1**	**Mean 2**	**Mean Diff.**	**SE of diff.**	**n1**	**n2**	**q**	**DF**
CT vs. OBS	100.0	235.3	-135.3	51.12	4	5	3.743	11
CT vs. OBexT	100.0	61.49	38.51	51.12	4	5	1.065	11
OBS vs. OBexT	235.3	61.49	173.8	48.20	5	5	5.100	11

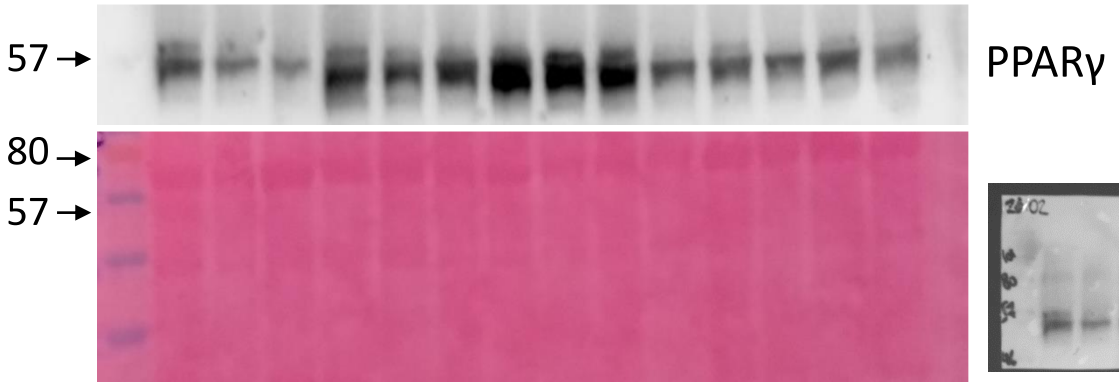

Original PPARγ

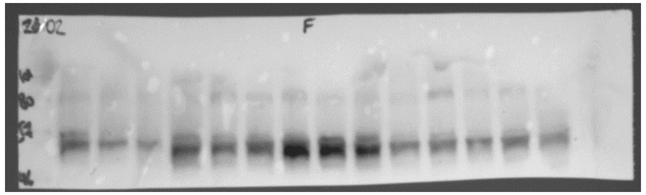

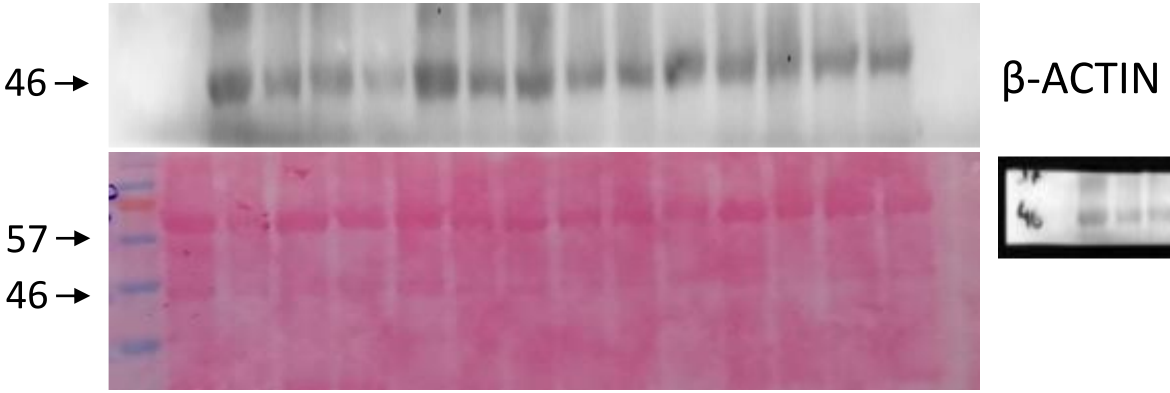

Original β-ACTIN

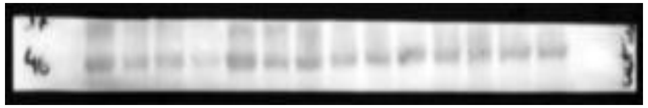

Fig. 6A and C

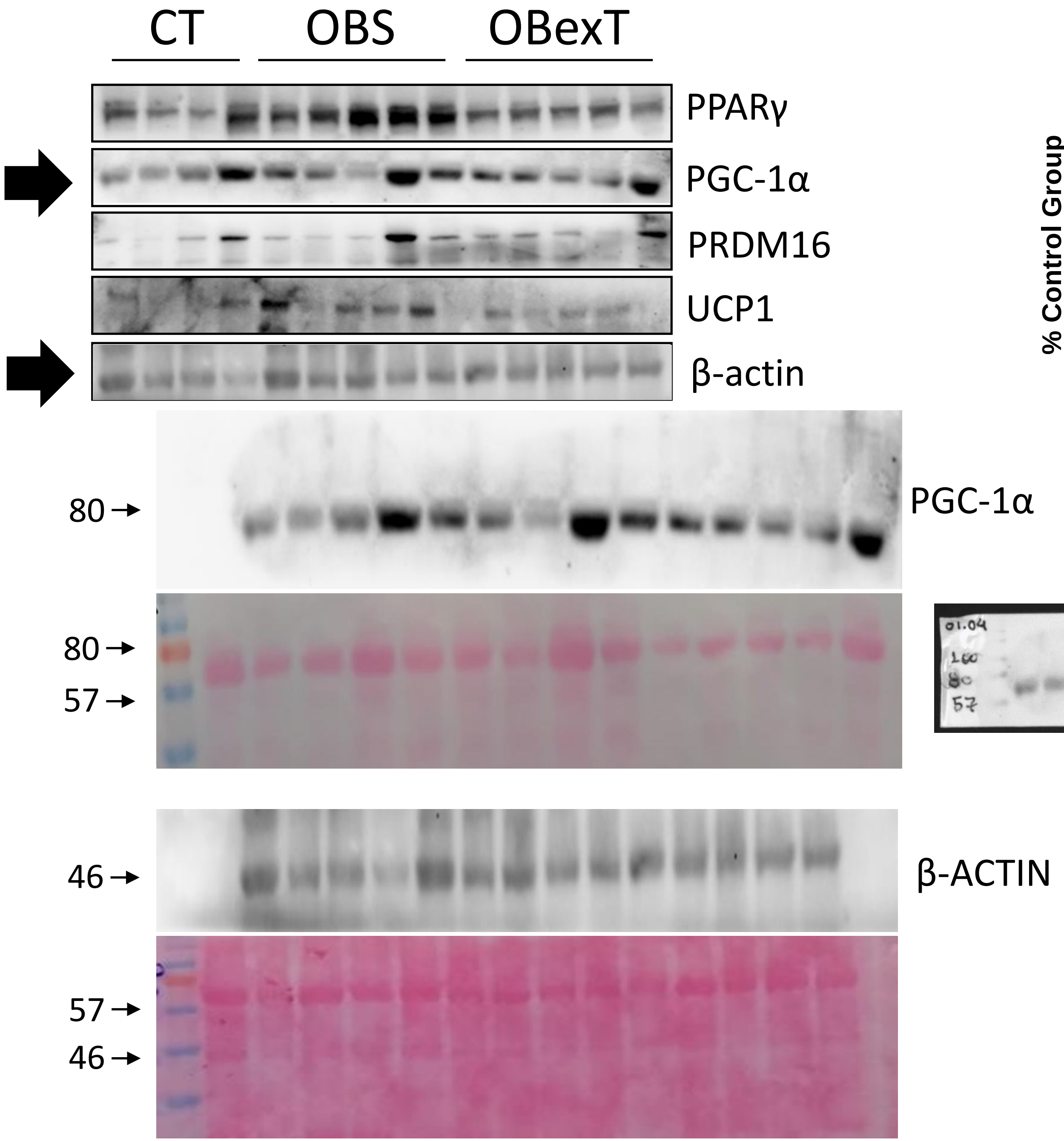

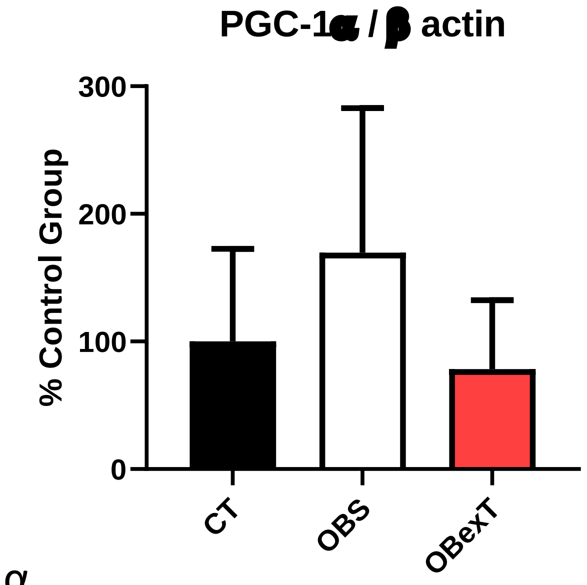

CT	OBS	OBexT
77.31078	351.2164	111.71510
75.38944	54.1691	42.79538
41.20941	108.4584	41.08689
206.09040	191.9128	38.73509
	142.9172	157.78820

Original PGC-1α

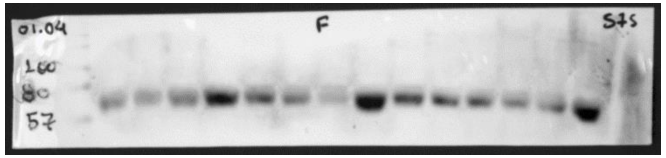

Tukey's multiple comparisons test	Mean Diff.	95.00% CI of diff.	Significant?	Summary	Adjusted P Value			
CT vs. OBS	-69.73	-223.0 to 83.55	No	ns	0.4617	E-F		
CT vs. OBexT	21.58	-131.7 to 174.9	No	ns	0.9240	E-G		
OBS vs. OBexT	91.31	-53.21 to 235.8	No	ns	0.2461	F-G		
**Test details**	**Mean 1**	**Mean 2**	**Mean Diff.**	**SE of diff.**	**n1**	**n2**	**q**	**DF**
CT vs. OBS	100.0	169.7	-69.73	56.75	4	5	1.738	11
CT vs. OBexT	100.0	78.42	21.58	56.75	4	5	0.5376	11
OBS vs. OBexT	169.7	78.42	91.31	53.51	5	5	2.413	11

Fig. 6A and D

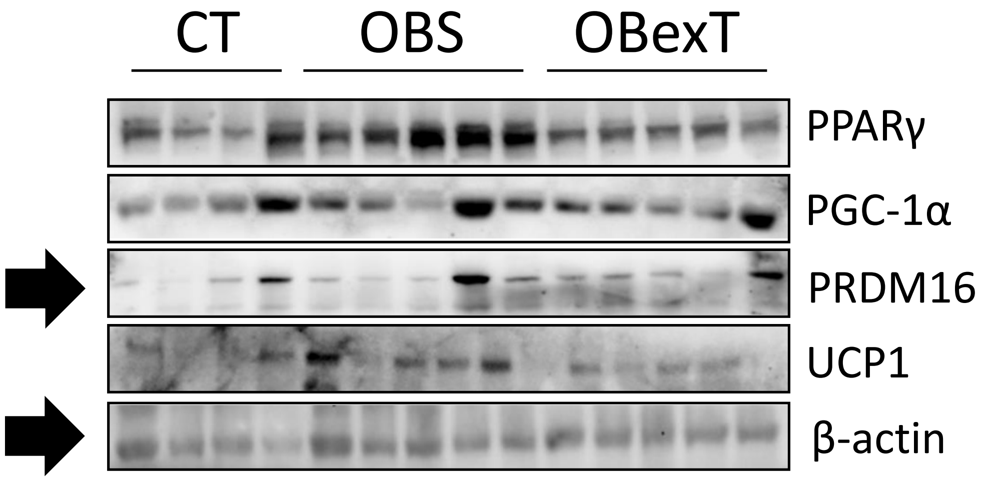

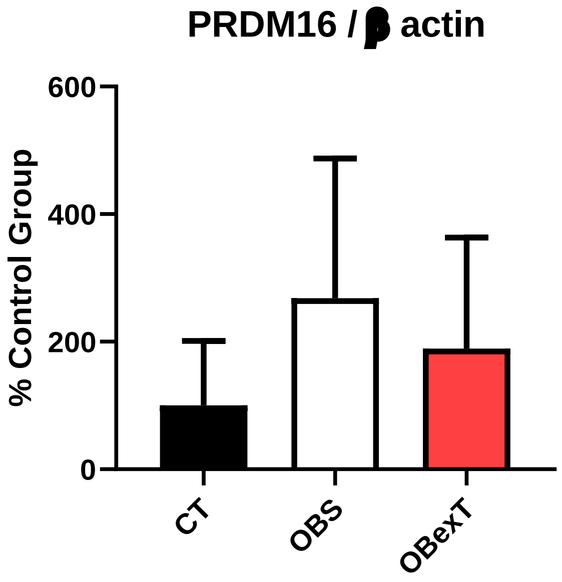

CT	OBS	OBexT
78.79396	280.07440	243.40850
26.10543	38.70540	91.24223
47.01094	110.32670	83.31519
48.08970	603.92660	56.37523
	308.26130	471.57410

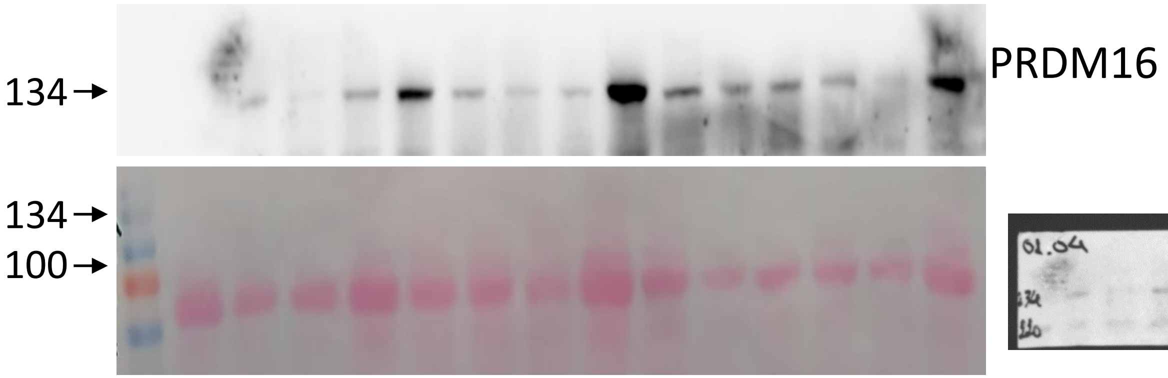

Original PRDM16

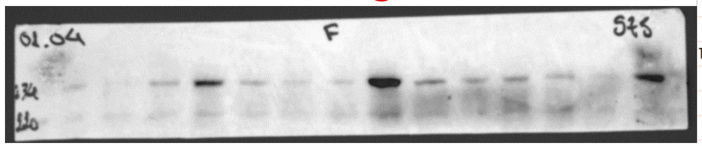

Tukey's multiple comparisons test	Mean Diff.	95.00% CI of diff.	Significant?	Summary	Adjusted P Value			
CT vs. OBS	-168.3	-488.6 to 152.1	No	ns	0.3653	I-J		
CT vs. OBexT	-89.18	-409.5 to 231.1	No	ns	0.7387	I-K		
OBS vs. OBexT	79.08	-222.9 to 381.1	No	ns	0.7643	J-K		
Test details	Mean 1	Mean 2	Mean Diff.	SE of diff.	n1	n2	q	DF
CT vs. OBS	100.0	268.3	-168.3	118.6	4	5	2.006	11
CT vs. OBexT	100.0	189.2	-89.18	118.6	4	5	1.063	11
OBS vs. OBexT	268.3	189.2	79.08	111.8	5	5	1.000	11

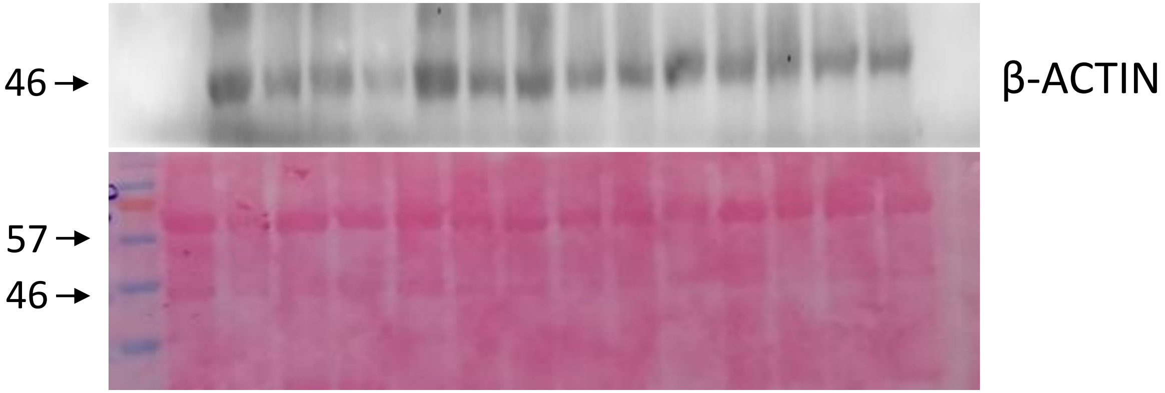

Fig. 6A and E

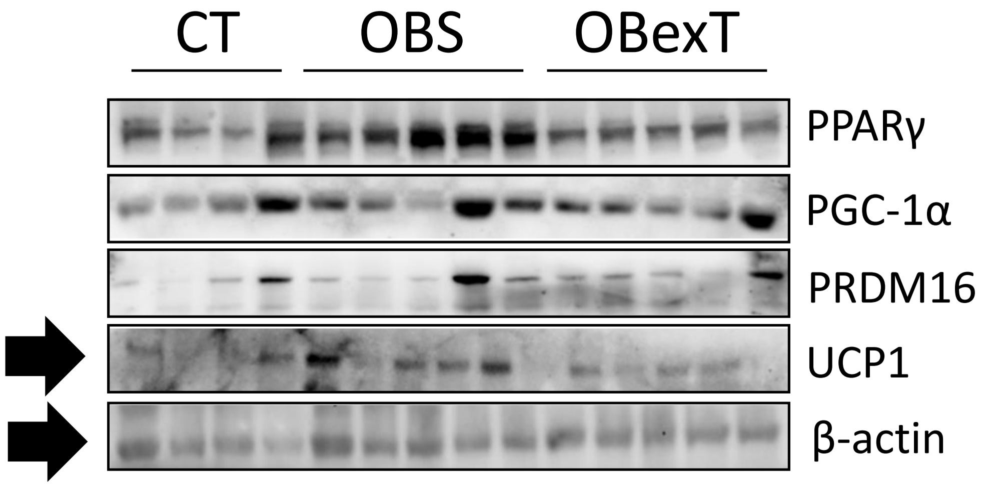

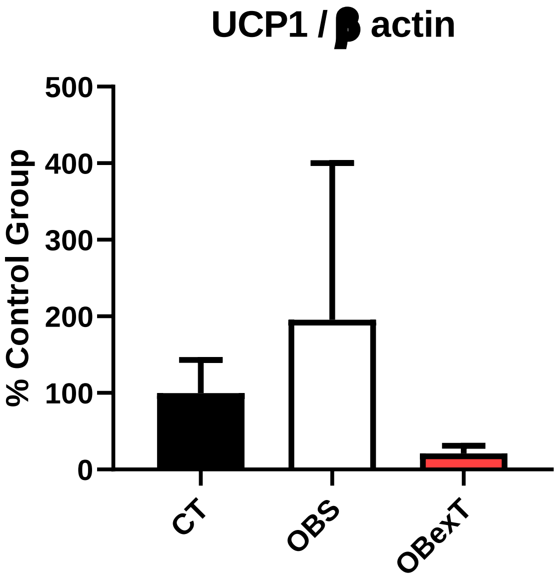

CT	OBS	OBexT
108.99350	550.49020	13.59455
102.99090	37.03126	23.13375
41.76868	165.05340	14.30663
146.24690	83.94919	17.85493
	142.35640	37.35893

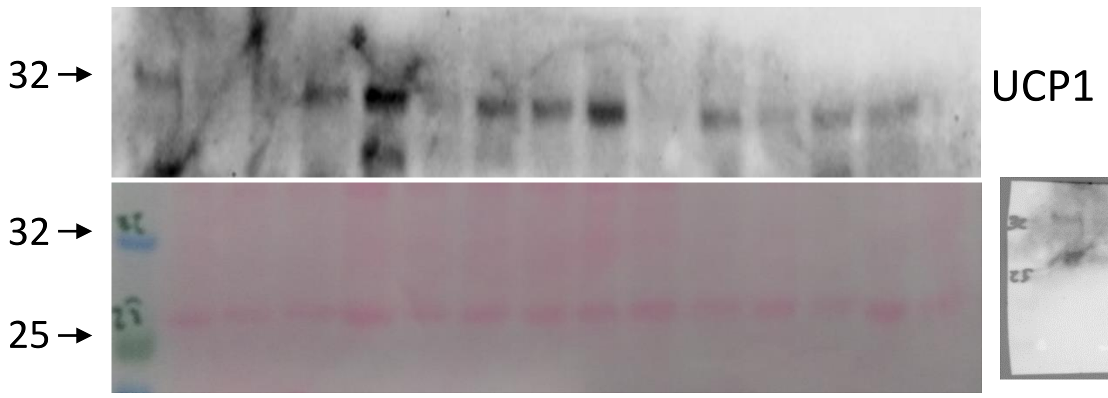

Original UCP1

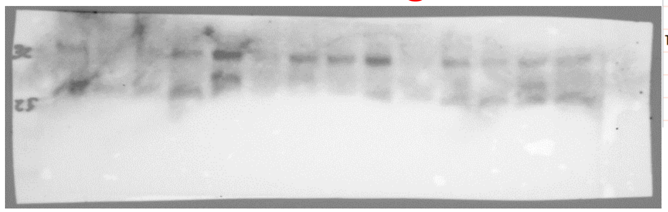

Tukey's multiple comparisons test	Mean Diff.	95.00% CI of diff.	Significant?	Summary	Adjusted P Value			
CT vs. OBS	-95.78	-323.2 to 131.7	No	ns	0.5123	M-N		
CT vs. OBexT	78.75	-148.7 to 306.2	No	ns	0.6304	M-O		
OBS vs. OBexT	174.5	-39.89 to 388.9	No	ns	0.1155	N-O		
Test details	Mean 1	Mean 2	Mean Diff.	SE of diff.	n1	n2	q	DF
CT vs. OBS	100.0	195.8	-95.78	84.21	4	5	1.609	11
CT vs. OBexT	100.0	21.25	78.75	84.21	4	5	1.323	11
OBS vs. OBexT	195.8	21.25	174.5	79.39	5	5	3.109	11

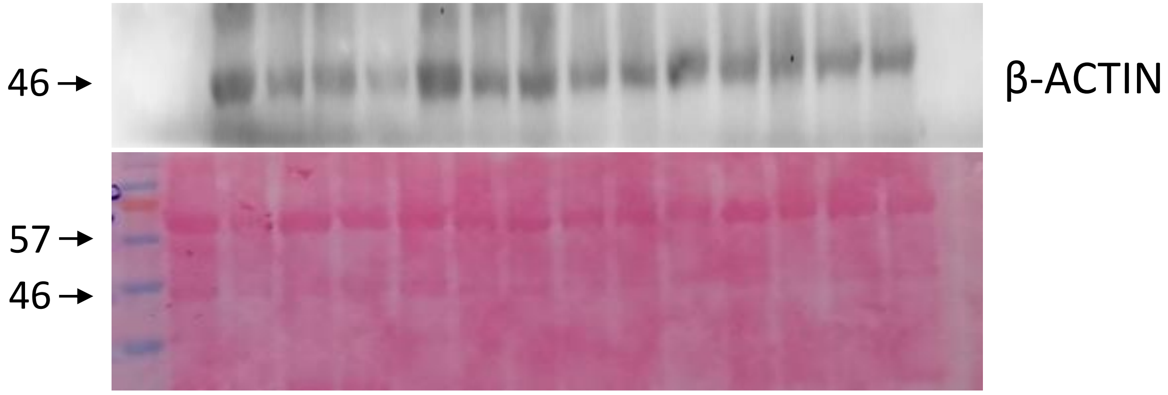

Fig. 6F

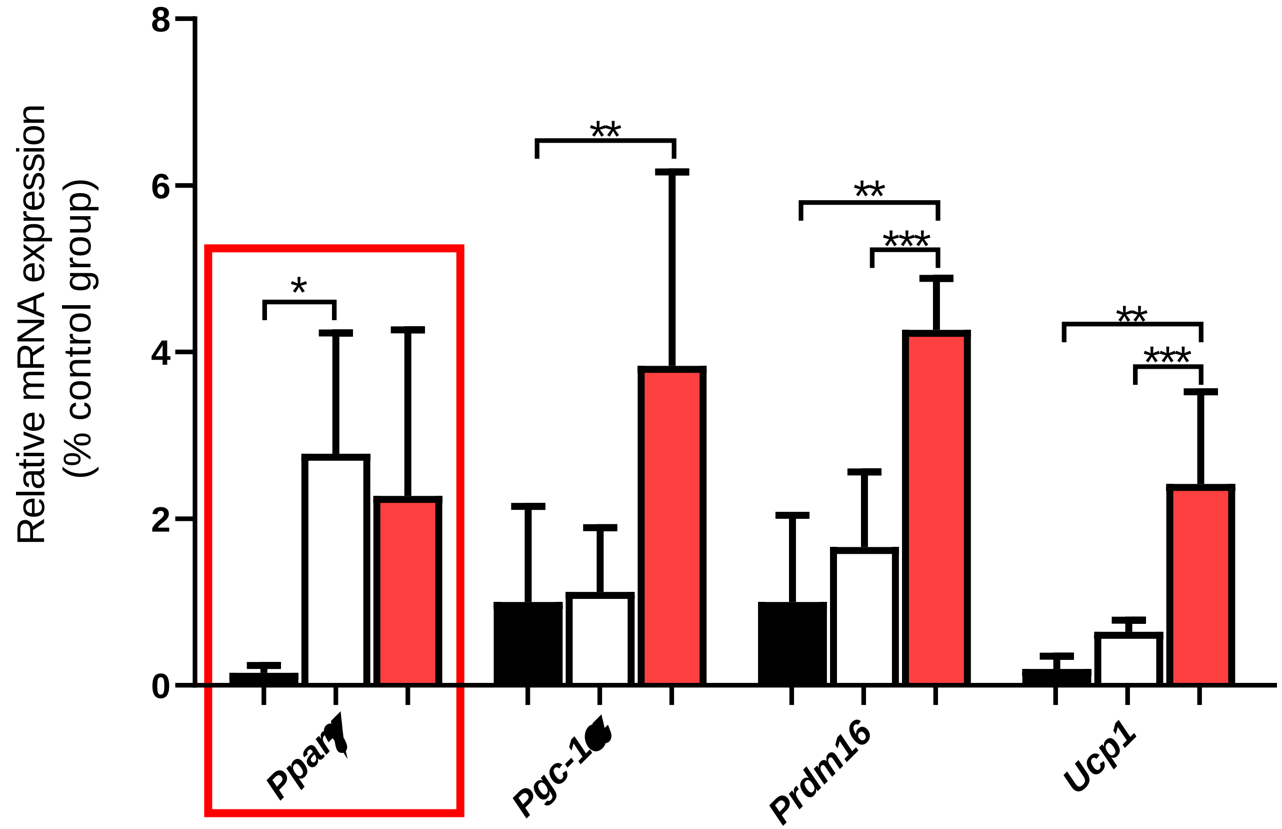

PPARγ

CT	OBS	OBexT
0.20	2.64	1.92
0.07	1.67	2.14
0.13	4.87	4.93
0.14	1.94	0.10
*6.11**		
0.07		
0.30		

Tukey's multiple comparisons test	Mean Diff.	95.00% CI of diff.	Significant?	Summary	Adjusted P Value			
CT vs. OBS	-2.628	-4.876 to -0.3804	Yes	*	0.0228	A-B		
CT vs. OBexT	-2.121	-4.369 to 0.1271	No	ns	0.0647	A-C		
OBS vs. OBexT	0.5075	-1.955 to 2.970	No	ns	0.8453	B-C		
**Test details**	**Mean 1**	**Mean 2**	**Mean Diff.**	**SE of diff.**	**n1**	**n2**	**q**	**DF**
CT vs. OBS	0.1517	2.780	-2.628	0.8323	6	4	4.466	11
CT vs. OBexT	0.1517	2.273	-2.121	0.8323	6	4	3.604	11
OBS vs. OBexT	2.780	2.273	0.5075	0.9117	4	4	0.7872	11

Cleaned data X Outliers

A	B	C	D	E
CT	OBS	OBexT		
6.110				

Fig. 6F

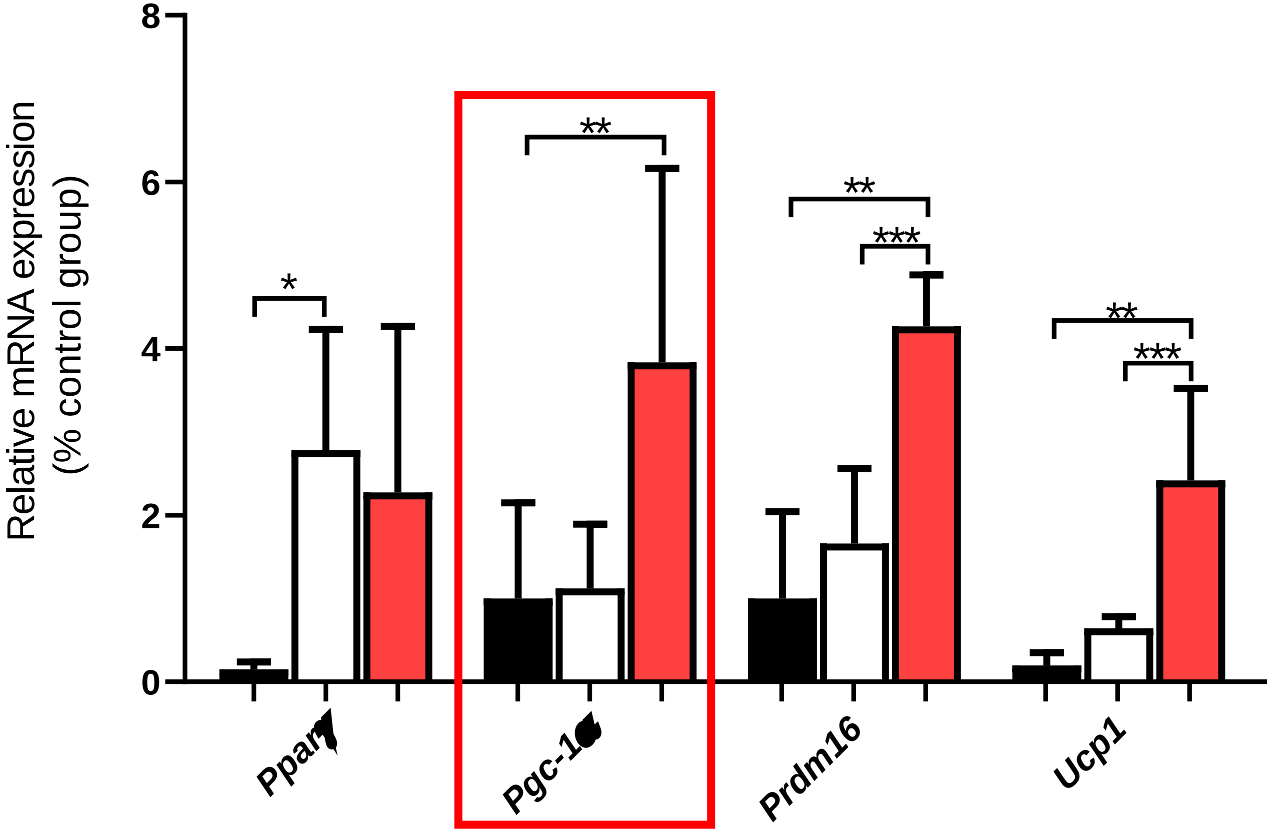

PGC-1α

CT	OBS	OBexT
0.38	0.70	0.70
0.21	1.87	5.89
3.13	1.66	3.48
2.06	0.25	5.27
0.39		
0.73		
0.10		

Tukey's multiple comparisons test	Mean Diff.	95.00% CI of diff.	Significant?	Summary	Adjusted P Value			
CT vs. OBS	-0.1200	-2.579 to 2.339	No	ns	0.9907	E-F		
CT vs. OBexT	-2.835	-5.294 to -0.3761	Yes	*	0.0242	E-G		
OBS vs. OBexT	-2.715	-5.489 to 0.05902	No	ns	0.0552	F-G		
**Test details**	**Mean 1**	**Mean 2**	**Mean Diff.**	**SE of diff.**	**n1**	**n2**	**q**	**DF**
CT vs. OBS	1.000	1.120	-0.1200	0.9217	7	4	0.1841	12
CT vs. OBexT	1.000	3.835	-2.835	0.9217	7	4	4.350	12
OBS vs. OBexT	1.120	3.835	-2.715	1.040	4	4	3.693	12

Fig. 6F

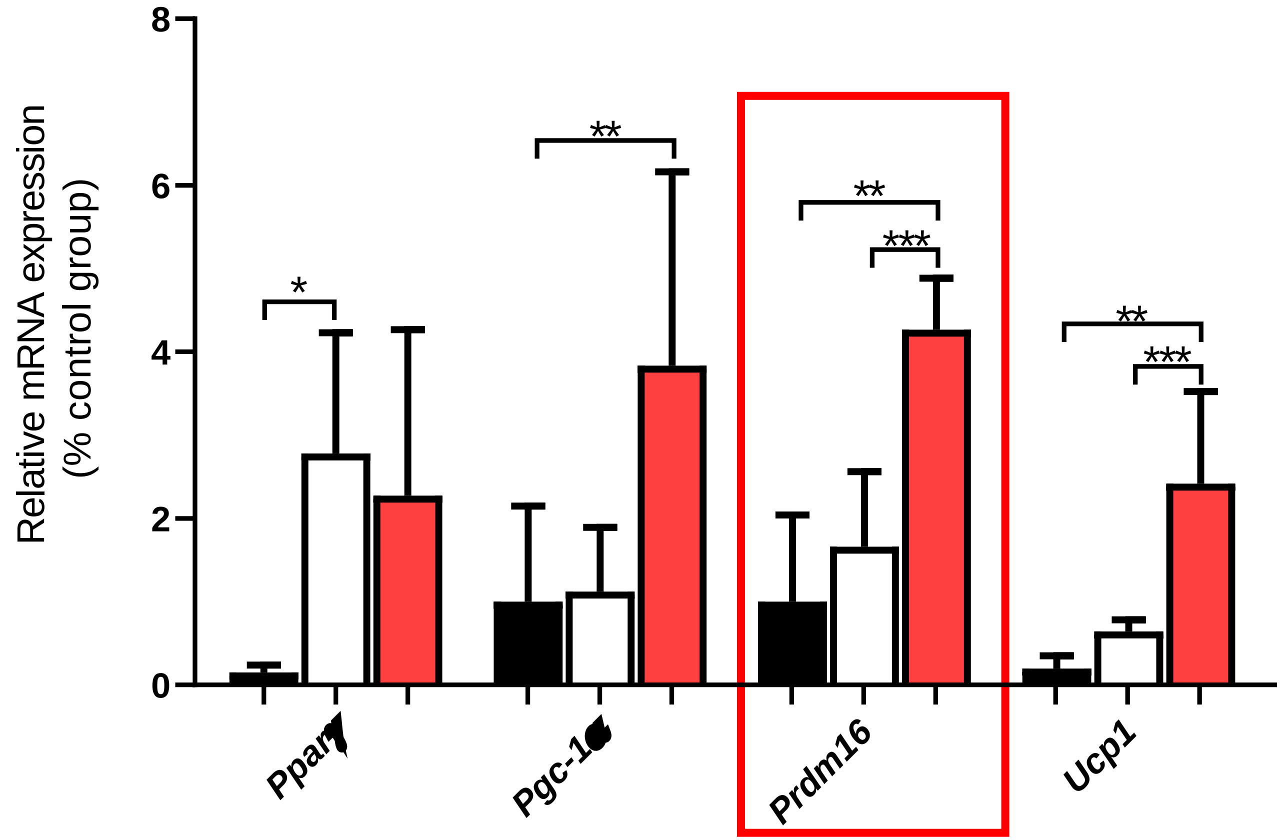

PRDM16

CT	OBS	OBexT
0.33	1.44	4.26
2.72	2.65	5.14
2.07	*11.78**	3.81
0.12	0.89	3.85
1.25		
0.28		
0.24		

Tukey's multiple comparisons test	Mean Diff.	95.00% CI of diff.	Significant?	Summary	Adjusted P Value			
CT vs. OBS	-0.6586	-2.368 to 1.051	No	ns	0.5681	I-J		
CT vs. OBexT	-3.264	-4.816 to -1.711	Yes	***	0.0004	I-K		
OBS vs. OBexT	-2.605	-4.497 to -0.7132	Yes	**	0.0087	J-K		
**Test details**	**Mean 1**	**Mean 2**	**Mean Diff.**	**SE of diff.**	**n1**	**n2**	**q**	**DF**
CT vs. OBS	1.001	1.660	-0.6586	0.6328	7	3	1.472	11
CT vs. OBexT	1.001	4.265	-3.264	0.5748	7	4	8.029	11
OBS vs. OBexT	1.660	4.265	-2.605	0.7004	3	4	5.260	11

Cleaned data × Outliers

A	B	C	D	E
OBS				
11.780				

Fig. 6F

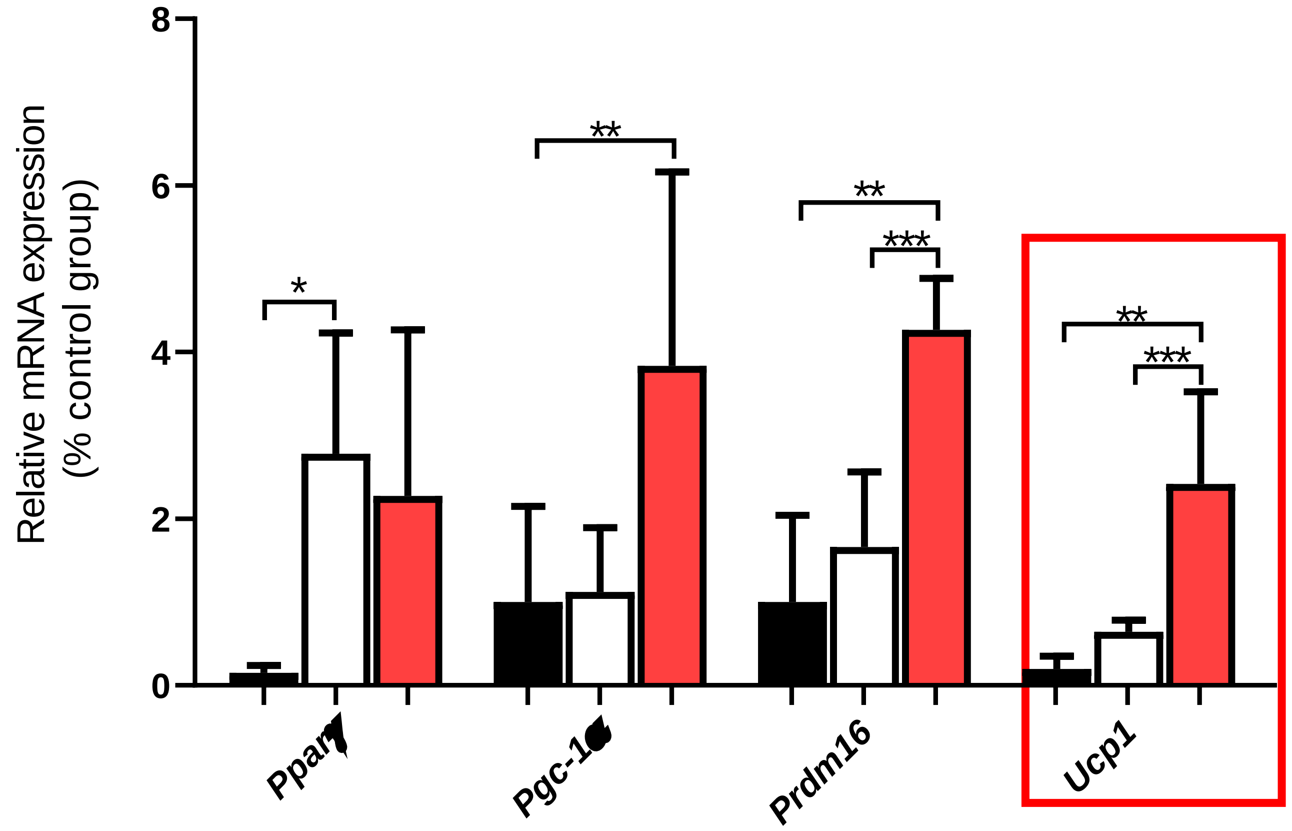

UCP1

CT	OBS	OBexT
2.71*	0.61	1.38
0.05	0.64	3.58
3.31*	0.49	2.29
0.01	0.83	46.90*
0.33		
0.28		
0.31		

Tukey's multiple comparisons test	Mean Diff.	95.00% CI of diff.	Significant?	Summary	Adjusted P Value	
CT vs. OBS	-0.4465	-1.453 to 0.5597	No	ns	0.4615	M-N
CT vs. OBexT	-2.221	-3.316 to -1.125	Yes	***	0.0008	M-O
OBS vs. OBexT	-1.774	-2.920 to -0.6286	Yes	**	0.0049	N-O

Test details	Mean 1	Mean 2	Mean Diff.	SE of diff.	n1	n2	q	DF
CT vs. OBS	0.1960	0.6425	-0.4465	0.3604	5	4	1.752	9
CT vs. OBexT	0.1960	2.417	-2.221	0.3923	5	3	8.005	9
OBS vs. OBexT	0.6425	2.417	-1.774	0.4103	4	3	6.115	9

Cleaned data × Outliers

A	B	C	D	E
CT				
2.710				

Cleaned data × Outliers

A	B	C	D	E
OBexT				
46.900				

Cleaned data × Outliers

A	B	C	D	E
CT				
3.310				

The ARRIVE Essential 10: Compliance Questionnaire

ARRIVE

National Centre for the Replacement Refinement & Reduction of Animals In Research

Use this questionnaire to evaluate how well a manuscript complies with the ARRIVE Essential 10. It can be applied to any manuscript describing comparative experiments in living animals, by assessors such as journal staff, editors, or peer reviewers.

Item		Question(s)	Answers
1	Study Design	Are all experimental and control groups clearly identified?	☒ Yes, for at least one experiment<br>☐ No
		Is the experimental unit (e.g. an animal, litter or cage of animals) clearly identified?	☒ Yes, for at least one experiment<br>☐ No
2	Sample Size	Is the exact number of experimental units in each group at the start of the study provided (e.g. in the format '*n*=')?	☒ Yes, for at least one experiment<br>☐ No
		Is the method by which the sample size was chosen explained?	☒ Yes, for at least one experiment<br>☐ No
3	Inclusion & Exclusion Criteria	Are the criteria used for including and excluding animals, experimental units, or data points provided?	☒ Yes, for at least one experiment<br>☐ No
		Are any exclusions of animals, experimental units, or data points reported, or is there a statement indicating that there were no exclusions?	☒ Yes, for at least one analysis<br>☐ No
4	Randomisation	Is the method by which experimental units were allocated to control and treatment groups described?	☐ Yes, for at least one experiment<br>☒ No
5	Blinding	Is it clear whether researchers were aware of, or blinded to, the group allocation at any stage of the experiment or data analysis?	☒ Yes, for at least one experiment<br>☐ No
6	Outcome Measures	For all experimental outcomes presented, are details provided of exactly what parameter was measured?	☒ Yes, for at least one experiment<br>☐ No
7	Statistical Methods	Is the statistical approach used to analyse each outcome detailed?	☒ Yes, for at least one analysis<br>☐ No
		Is there a description of any methods used to assess whether data met statistical assumptions?	☒ Yes, for at least one analysis<br>☐ No<br>☐ Not applicable
8	Experimental Animals	Are all species of animal used specified?	☒ Yes, for at least one experiment<br>☐ No
		Is the sex of the animals specified?	☒ Yes, for at least one experiment<br>☐ No<br>☐ Not applicable to species
		Is at least one of age, weight or developmental stage of the animals specified?	☒ Yes, for at least one experiment<br>☐ No
9	Experimental Procedures	Are both the timing and frequency with which procedures took place specified?	☒ Yes, for at least one experiment<br>☐ No
		Are details of acclimatisation periods to experimental locations provided?	☒ Yes, for at least one experiment<br>☐ No
10	Results	Are descriptive statistics for each experimental group provided, with a measure of variability (e.g. mean and SD, or median and range)?	☒ Yes, for at least one experiment<br>☐ No<br>☐ Not applicable to the type of data collected
		Is the effect size and confidence interval provided?	☒ Yes, for at least one experiment<br>☐ No<br>☐ Not applicable to the type of analysis used

Notes on questionnaire design

The ARRIVE guidelines are a useful resource for authors preparing manuscripts describing animal research, and also provide a framework to evaluate the transparency of those manuscripts. To assess reporting quality, numerous studies have in the past sought to operationalise reporting guidelines (including ARRIVE). Typically, this involves scoring a manuscript's degree of compliance with guideline items in a binary fashion (e.g. an item is either not reported or reported) [1-3], a graded fashion (e.g. not, partially, or completely reported) [4,5], or a combination of the two [6].

This questionnaire has been designed to be as concise and user-friendly as possible. The number of questions used to assess a manuscript's compliance has been kept to a minimum, and in most cases each question is designed to be answered in a binary fashion. Compliance with some Essential 10 sub-items is inherently impossible to judge in this way, instead requiring a subjective judgement on the level of detail provided. For this reason, not all sub-items are represented by a question in this questionnaire.

To facilitate binary answers, it has been necessary to identify the minimum information in a manuscript sufficient to comply with each question. The strengths of this approach include the relatively short length of the questionnaire (and the correspondingly low time burden of using it), and the avoidance of ambiguity that would arise from a graded answering system, in which an intermediate score (e.g. 'partially/insufficiently reported') could denote a number of distinct deficiencies in compliance with an item (e.g. either only part of the item was complied with, or only the reporting of some experiments in the manuscript complied with the item.)

Limitations of this approach centre on the necessity to identify the minimum information sufficient to comply with each question. In some cases, this has resulted in questions that require a guideline sub-item's criteria to have been fulfilled in the reporting of only one experiment in a manuscript. As a result, not all experiments in a manuscript may be described in a way that fulfils that criterion, despite the manuscript being considered to comply with the guidelines overall.

References

1. Hair *et al* (2020). *Res Integ Peer Rev*. doi: 10.1186/s41073-019-0069-3
2. Tihanyi *et al* (2019). *J Surg Res*. doi: 10.1016/j.jss.2018.10.038
3. Zhao *et al* (2020). *BMC Vet Res*. doi: 10.1186/s12917-020-02664-1
4. Han *et al* (2017). *Plos One*. doi: 10.1371/journal.pone.0183591
5. Chatzimanouil *et al* (2019). *J Am Soc Nephrol*. doi: 10.1681/ASN.2018050515
6. Leung *et al* (2018). *Plos One*. doi: 10.1371/journal.pone.0197882
